# Bi-order multimodal integration of single-cell data

**DOI:** 10.1186/s13059-022-02679-x

**Published:** 2022-05-09

**Authors:** Jinzhuang Dou, Shaoheng Liang, Vakul Mohanty, Qi Miao, Yuefan Huang, Qingnan Liang, Xuesen Cheng, Sangbae Kim, Jongsu Choi, Yumei Li, Li Li, May Daher, Rafet Basar, Katayoun Rezvani, Rui Chen, Ken Chen

**Affiliations:** 1 Department of Bioinformatics and Computational Biology, The University of Texas MD Anderson Cancer Center, Houston, USA; 2 Department of Molecular and Human Genetics, Baylor College of Medicine, Houston, TX 77030 USA; 3 Department of Stem Cell Transplantation and Cellular Therapy, The University of Texas MD Anderson Cancer Center, Houston, TX USA; 4 Verna and Marrs McLean Department of Biochemistry and Molecular Biology, Baylor College of Medicine, Houston, TX 77030 USA

**Keywords:** Single-cell multi-omics, Bi-order canonical correlation analysis, Cell type identity

## Abstract

Integration of single-cell multiomics profiles generated by different single-cell technologies from the same biological sample is still challenging. Previous approaches based on shared features have only provided approximate solutions. Here, we present a novel mathematical solution named bi-order canonical correlation analysis (bi-CCA), which extends the widely used CCA approach to iteratively align the rows and the columns between data matrices. Bi-CCA is generally applicable to combinations of any two single-cell modalities. Validations using co-assayed ground truth data and application to a CAR-NK study and a fetal muscle atlas demonstrate its capability in generating accurate multimodal co-embeddings and discovering cellular identity.

**Supplementary Information:**

The online version contains supplementary material available at 10.1186/s13059-022-02679-x.

## Background

Advances in high-throughput single-cell technology such as single-cell RNA-sequencing (scRNA-seq) [1] and mass cytometry [2] have enabled systematic delineation of cell types based on thousands to millions of cells sampled from developing organisms or patient biopsies [3, 4]. For example, recent application of combinatorial indexing-based technology has generated the transcriptomic and chromatin accessibility profiles of millions of cells in developing human fetus samples [5]. Rare cell types and complex cellular states, however, remain challenging to discover, which necessitates the development of multiomics technologies to simultaneously measure other cellular features, including DNA methylation [6, 7], chromatin accessibility [8–10], and spatial positions [11, 12] in the same cells. Although available single-cell multiomics technologies [10, 13–16] can profile thousands to millions of cells per experiment, the cost of the experiments is still quite high [17], and the data generated are often of lower throughput than those generated by unimodal technologies. These restrictions necessitate the development of computational approaches that can accurately integrate multiple data matrices generated by different technologies from the same biological samples to acquire an accurate characterization of cellular identity and function.

However, different technologies create data matrices of different rows and columns, which correspond to different sets of cells and different types of features. How to align cells and features simultaneously across matrices is a core computational challenge. When the two sets of cells are sampled uniformly from the same biological sample, it is safe to assume that there exists an optimal alignment of them. However, the search space, whose dimensionality is the product of the numbers of cells (or the numbers of features) in the two sets, is extremely large. To address this challenge, existing computational approaches followed two directions [18]: (1) aligning features empirically before aligning cells [19–22] and (2) obtaining separate embeddings for each modality, followed by performing unsupervised manifold alignment [23–25]. Taking integration of scRNA-seq and single cell assay for transposase accessible chromatin sequencing (scATAC-seq) as an example, the first category of methods require constructing a “gene activity matrix” from scATAC-seq data by counting DNA reads aligned near and within each gene [26]. A successful alignment requires considering both basic proximal regulatory elements and distal regulatory relationship established via other regulatory elements such as enhancers, which are often critical to decipher cell identities [8]. However, current approaches either completely rely on proximal regulatory elements, or infer distal elements from only scATAC-seq data (e.g., Cicero [26]) without integrating with gene expression data. It also substantially simplifies (or loses) multifactorial relations between transcription factors (TF) and target genes [27]. Based on pre-aligned features generated by such empirical rules, Seurat integration (referred to as “Seurat” here after; not to be confused with the weighted nearest neighbor (WNN) approach introduced in Seurat v4 for clustering co-assayed data) applies canonical correlation analysis (CCA) and mutual nearest neighbors (MNNs) to identify cells anchoring the two data matrices [20]; LIGER uses an integrative non-negative matrix factorization (iNMF) to delineate shared and dataset-specific features [22]. Coupled NMF shares similar concept with LIGER [28]; Harmony projects cells onto a shared embedding using principle components analysis (PCA) and removes batch effects iteratively [21]. All these programs suffer from the aforementioned limitations and thereby cannot yield a comprehensive, bi-order gene regulatory network, particularly when chromatin changes are asynchronous from RNA transcriptions in cells undergoing state transitions [29]. The second category of methods such as MATCHER, MMD-MA, UnionCom, SCOT, and Pamona [24, 25, 30–32] do not require prior feature alignment. However, they only use intramodal pairwise cell-cell distance information and discard intermodal, trans-acting feature interaction. Thus, they may misalign cell types of similar abundance instead of similar biology, especially rare cell types.

In this study, we develop a novel method called bi-CCA (bi-order canonical correlation analysis) and associated computational tool called bindSC. Bi-CCA learns the optimal alignment among rows and columns (i.e., both cell correspondence and feature interactions) from two data matrices generated by two different experiments. The alignment matrix derived from bi-CCA can thereby be utilized to derive in silico multiomics profiles from aligned cells, which can be used as input to downstream regulatory network inference.

We first assess our method on multimodality integration tasks using benchmarking datasets obtained directly from multiomics technologies, including a novel mouse retinal bipolar cell dataset created by the 10x Genomics Multiome ATAC+RNA kit. Unlike existing integration methods using shared features only, bi-CCA utilizes the full feature information and enables accurate alignment of bipolar cell subtypes between RNA and ATAC data. It also enables discovery of novel cell-type-delineating gene-protein links via integration of RNA and mass cytometry data. We next apply bindSC to two challenging integration tasks. It detects an active immune cell population in the CAR-NK cell products via integration of RNA and mass cytometry data; it resolves mislabeled fetal muscle cells via integration of RNA and ATAC profiles. Bi-CCA is implemented as an open-source R package bindSC available at https://github.com/KChen-lab/bindSC.

## Results

### Bi-order integration of multi-omics data

**Fig. 1:**
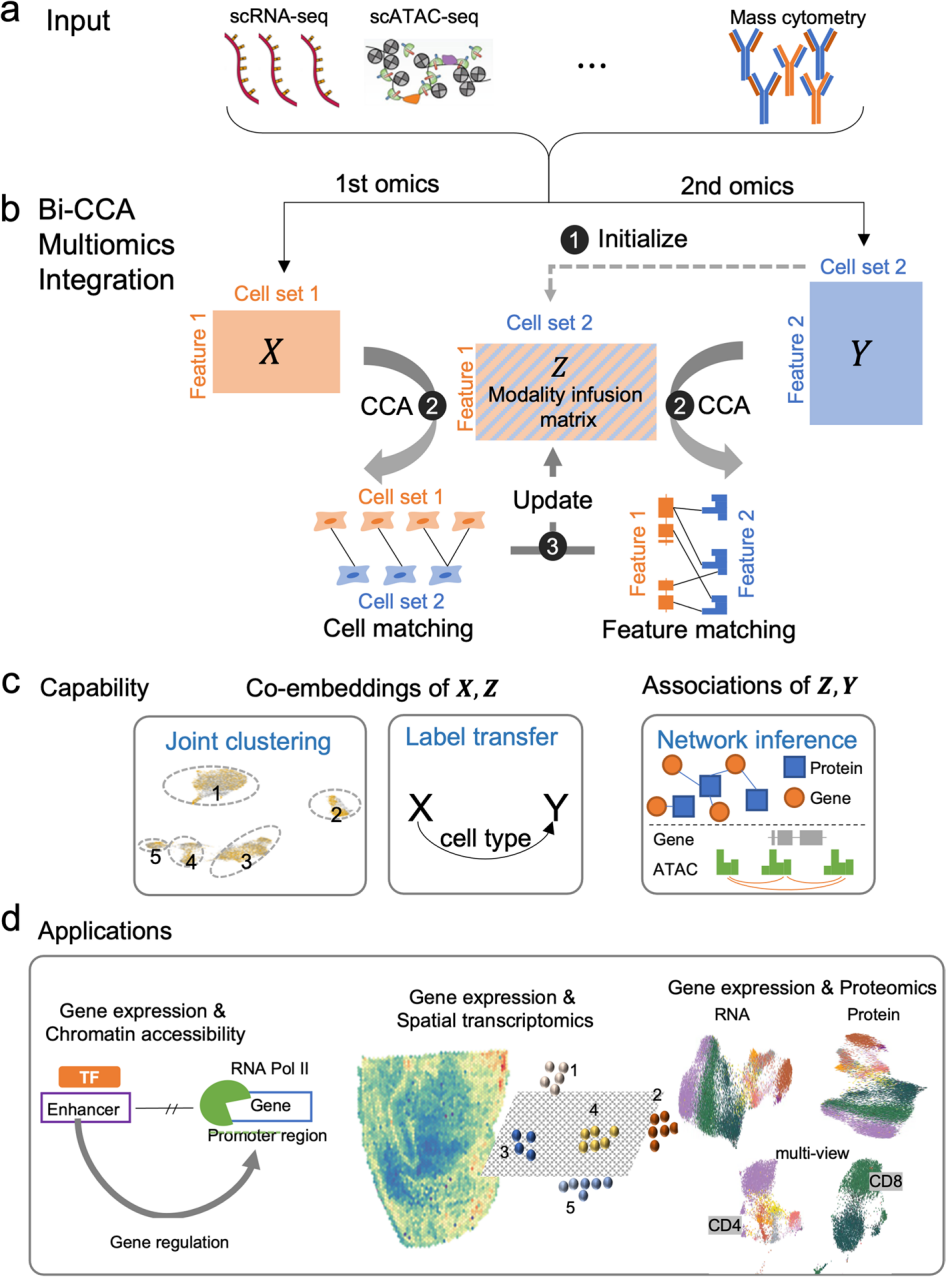
Overview of bindSC. **a** Inputs supported by bindSC. BindSC can integrate two single-cell assays such as transcriptomes, epigenomes, spatial transcriptomes, and proteomes. **b** Bi-order integration of two modalities (***X*** and ***Y***) with unpaired cells and unmatched features using the bi-CCA approach. In the data matrices, each row represents a gene/locus, and each column represents a cell. **Step 1**: initializing a modality fusion matrix ***Z*** linking the two modalities (Methods). **Step 2**: matching both cells and features across modalities using CCA. **Step 3**: updating ***Z*** using the obtained cell-cell and feature-feature matching results. Steps 2 and 3 are performed iteratively to optimize ***Z***. **c** Based on canonical correlation vectors (CCVs) in the derived latent space, bindSC can (1) jointly cluster cells in both modalities to define cell types and (2) transfer labels from one modality to another modality. Association of ***Z*** and *Y* measured in the same cell enables to infer gene-protein and peak-gene regulatory networks. **d** The integrated multiomics feature profiles enable us to (1) link genes to regulatory elements, (2) map RNA expressions to spatial locations, and (3) delineate cells by both RNA and protein signatures

Bi-CCA takes as input two single-cell data matrices (**X** and **Y**) generated uniformly from the same cell population by two different technologies (Fig. 1a and Additional file 1: Fig. S1). In most single-cell multi-omics integration tasks, neither the alignment between the cells in **X** and those in **Y**, nor the alignment between the features in **X** and those in **Y** is known (Additional file 2: Supplementary Note 1). To address this challenge, bi-CCA introduces a modality fusion matrix **Z** to link **X** and **Y** (Fig. 1b). The modality fusion matrix has the same rows as does **X** and the same columns as does **Y**. To facilitate the optimization of **Z**, it is initialized based on prior knowledge linking the two modalities. Taking integration of scRNA-seq and scATAC-seq as an example, the modality fusion matrix can be initialized to the “gene activity matrix” estimated by other programs such as Seurat v3.0. Bi-CCA then iteratively updates **Z** to find an optimal solution which maximizes the correlation between **X** and **Z** and between **Y** and **Z** in the latent space simultaneously. Details about this iterative procedure can be found in Methods. In silico simulation experiments using splatter [33] indicate that bi-CCA can robustly align cells and discover meaningful feature interactions from noisy experimental data (Additional file 2: Supplementary Note 2 and Additional file 1: Fig. S2).

Bi-CCA outputs canonical correlation vectors (CCVs), which project cells from two datasets onto a shared latent space (hereafter “co-embedding”). Joint clustering, label transfer and network inference can be done in the latent space (Fig. 1c). Moreover, the final modality fusion **Z** and **Y** can generate a consensus multiomic profile for cells from **Y** directly, thus enable (1) characterizing gene and chromatin-accessibility relations from aligned scRNA-seq and scATAC-seq data, (2) associating transcriptomic profiles with proteomic profiles from aligned scRNA-seq and CyTOF data, (3) associating transcriptomic profiles with spatial locations from aligned scRNA-seq and spatial transcriptomic data, and so on (Fig. 1d).

### Integration of single-cell RNA-seq and single-cell ATAC-seq data

To examine the utility of bindSC on integrating scRNA-seq and scATAC-seq data, we generated coassayed snRNA-seq and snATAC-seq data using the 10x Genomics Multiome ATAC+RNA kit from an adult mouse retina sample. Mouse retina is heterogeneous, composed of multiple neuronal and non-neuronal cell types [6, 34, 35]. Among them, bipolar cells (BC), which connect photoreceptors (cones and rods) to inner retina, are traditionally dissected into rare subtypes of subtle functional and morphological differences. While high-resolution single-cell transcriptomic profiles of BCs are available [34, 36–38], little is known about the corresponding single-cell chromatin landscapes. Although it is now possible to directly generate multiome data, there are often restrictions on cost, feasibility, and data quality. Therefore, integrating single-cell ATAC and RNA profiles obtained independently from the same retina sample may provide an exciting opportunity to comprehensively characterize these rare cell subtypes and discover transcription factors (TFs) important in establishing or maintaining the cell identities [39–41].

**Fig. 2:**
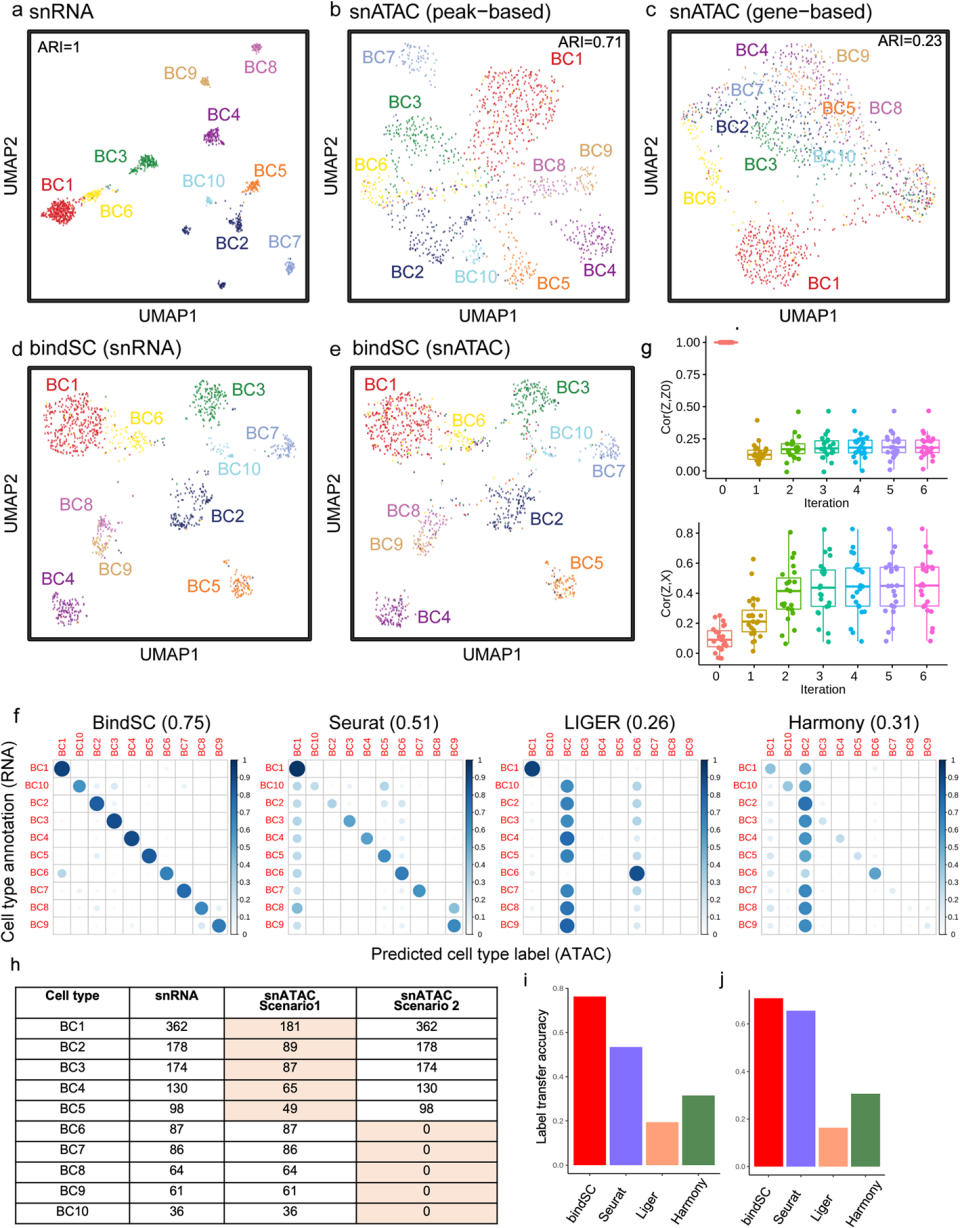
Integrating snRNA-seq and snATAC-seq data from bipolar cells in a mouse retinal sample. **a**–**c** The UMAP views of the snRNA-seq profiles (**a**), the snATAC chromatin accessibility peak profiles (**b**), and the compressed gene-based chromatin accessibility profiles (**c**) of 1276 mouse retinal bipolar cells. The cells are colored by cell types annotated based on RNA expression levels (ground truth, Additional file 1: Fig. S3). The Adjusted Rand Index (ARI) values are labeled in each panel. Given we use RNA annotation as the gold standard, the ARI for RNA clustering in (**a**) is 1. **d**, **e** UMAPs generated from the bindSC-integrated snRNA and snATAC co-embeddings. Plotted respectively are cells in the snRNA-seq data (**d**) and those in the snATAC-seq data (**e**). **f** Consistency between cell types computationally inferred from ATAC profiles by bindSC, Seurat v3.0, LIGER, and Harmony, respectively, with the ground truth. Darkness of the dots corresponds to degree of consistency while size of the dots the fraction of cells per row. Overall accuracies are shown in the subtitle after the method names. **g** Pearson correlation coefficients between the imputed and the initial gene score (top panel) and the ground truth RNA profiles (bottom panel) over bindSC iterations. The result at iteration 0 corresponds to the traditional CCA method. Each dot corresponds to the accuracy of one known marker gene (The full gene list is shown in Additional file 1: Fig. S3). **h** Downsampling schemes to generate imbalanced datasets between snRNA-seq and snATAC-seq data. Each value in the table denotes cell number available. We generated two imbalanced data integration scenarios: (1) downsampling 50% of the five major cell types (BC1-5) while keeping all the cells from the five minor cell types (BC6-10) and (2) removing the five minor cell types (BC6-10) in the snATAC-seq data. Label transfer accuracy achieved by various methods on these imbalanced datasets are shown for scenario 1 (**i**) and scenario 2 (**j**)

After performing standard quality control, we obtained 1276 BC nuclei of high-quality matched ATAC+RNA profiles, which serve as an objective ground truth for quantifying the success of in silico integration. We first examined the RNA profile. Ten clear clusters were identified and annotated unambiguously as BC1-10 (Fig. 2a and Additional file 1: Fig. S3). Thus, this RNA-based cell type annotation was used as a ground truth in the subsequent analysis. We then examined the ATAC profiles and found that cells in the same cell-types were largely clustered together (ARI = 0.71) although were not as distinctive. When we reduced the ATAC data to gene resolution based on proximity to nearest genes, the cell types became harder to delineate (ARI = 0.23; Fig. 2c), indicating that the gene activity transformation loses information.

To evaluate bindSC and three other commonly used methods (Seurat v3.0, LIGER, Harmony) in the task of integrating two independent single-cell dataset, we treated the snRNA and snATAC data as if they were obtained from two different set of cells and tested the ability of these methods in recovering the known pairing. A successful method should project the cells of the same type into the same region in the integration space. As shown in the co-embedding UMAPs (Fig. 2d, e), bindSC successfully achieved that. In the UMAPs generated from the co-embeddings, both the RNA (Fig. 2d) and the ATAC (Fig. 2e) data achieved relatively tight clustering and distributed correspondingly by cell types. We compared cell-typing accuracy of each method (generated in the respective co-embeddings) with the ground truth. We found that bindSC achieved relatively accurate results (Fig. 2f). In comparison, Seurat v3.0 tended to misalign all cell types to BC1 and had difficulties separating BC8 and BC9. LIGER and Harmony have worse accuracy. These were due partly to the fact that these methods started with gene-based ATAC profiles, which already lost useful information (Fig. 2c).

Because bindSC works with the full ATAC profile, it has the power to better establish the relationship between the RNA and the ATAC features, including potentially distal relationships. To elucidate this point, we calculated the correlation between imputed RNA profiles (i.e., the fusion matrix **Z**) and the observed RNA profiles. As expected, RNA profiles imputed from gene-based ATAC profiles (at iteration 0) was weakly correlated with the observed RNA profiles (Pearson’s *R* = 0.1). After 3 iterations, the *R* value increased to 0.5; meanwhile, the value between imputed and the initial profile decreased to as low as 0.2, indicating the power of associating full peak profiles to genes in a de novo fashion, rather than utilizing reduced profiles (Fig. 2g).

To further examine bindSC’s performance on scenarios where cell populations have imbalanced abundance between two modalities, we generated two datasets: (1) removing 50% of cells in the top five major cell types (i.e., BC1, BC2, BC3, BC4, BC5) in the snATAC data while keeping the snRNA data intact and (2) removing the top five minor cell types (i.e., BC6, BC7, BC8, BC9, BC10) in the snATAC data while keeping the snRNA-seq data intact (Fig. 2h). The label transfer accuracy of bindSC was similar with that from the full paired profiles, indicating bindSC alignment is robust on imbalanced datasets. Again, bindSC had the best performance among all methods in these two scenarios (Fig. 2i–j; Additional file 1: Fig. S4d-e).

We also performed evaluation of several manifold-based methods (SCOT, UnionCom, Pamona, and MMD-MA). They tend to swap entire cell types (Additional file 1: Fig. S4a-b), especially for subtypes with similar abundances, such as BC6 and BC7 (both ~7% abundance; see Additional file 1: Fig. S4a) for SCOT. The mappings, though mathematically plausible, are not biologically sound.

We further examined the 16,944 de novo peak-gene links inferred by bindSC. They can be grouped into 25 clusters. Some of these links were distinct to cell types, while others were shared by multiple cell types (Additional file 1: Fig. S5), indicating potentially a hierarchical regulatory architecture resulting from staged cell lineage differentiation. Specific distal regulatory relations were found in those links, such as *Nfib* interacting with peaks up to 1Mb away and *Car8* interacting with peaks up to 250kb away [37] (Additional file 1: Fig. S6). The integration also enhanced the analysis of correlation between the RNA expression levels of transcription factors (TFs) and their activities inferred from DNA-binding motif enrichment analysis of the ATAC-seq profile (Methods; Additional file 1: Fig. S7).

Overall, our study demonstrated the power of bindSC in generating more accurate in silico multiomics profiles than other existing methods, and the potential in better delineating cell types and associated regulatory signatures.

#### Integration of single-cell RNA and epitope expression data

Complex interplay exists between mRNAs and proteins [42]. Single-cell proteomic methods such as mass cytometry (CyTOF) [2, 43] measure abundance of a small set of (often 10–50) surface proteins (epitopes) and provide functional quantification of various cell populations. Integrating single-cell RNA and protein data from the same sample can potentially achieve higher resolution characterization and enable discovery of novel cellular states and associated regulatory signatures. This task is challenging because the mRNA and protein expression levels derived from the same genes are not well correlated, due to complex post-transcriptional modifications and technological limitations [44]. CITE-seq [45] performs joint profiling of epitope and mRNA levels in the same cells and can be used to evaluate the results of in silico integration.

**Fig. 3:**
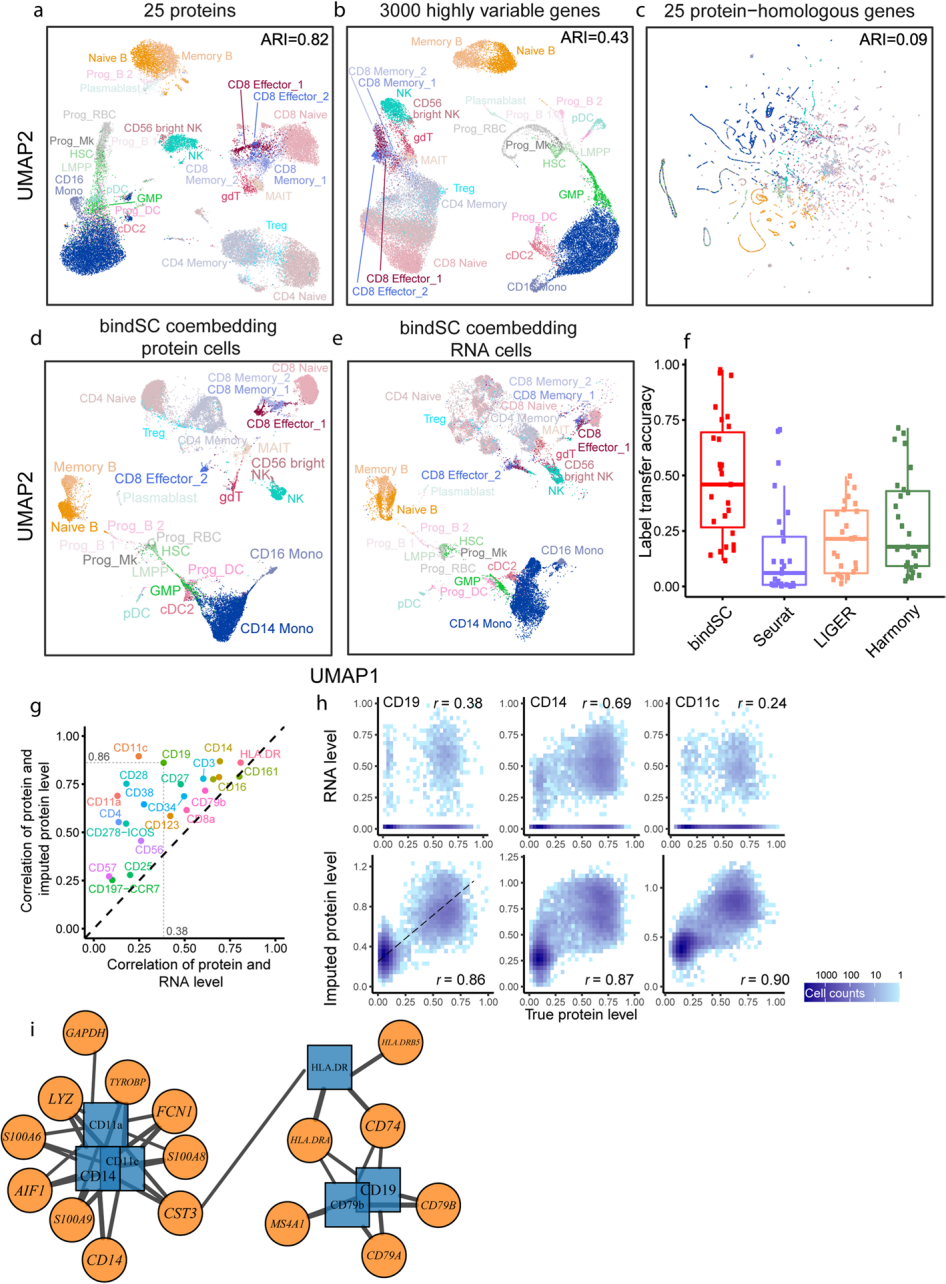
Integrating single-cell RNA with protein data produced by a CITE-seq assay. **a**–**c** UMAPs of 30,672 human bone marrow cells based on abundance of the 25 surface proteins (**a**), RNA expression levels of 3000 highly variable gene (**b**), and RNA expression levels of the 25 protein-coding genes (**c**). Labels and dots are colored synchronously by cell type information from the original study. The ARI values are labeled in each panel. **d**, **e** UMAPs of the protein (**d**) and the RNA (**e**) expression data in the co-embedding generated by bindSC. Each dot in the boxplot denotes one cell type. **f** Label transfer accuracy of bindSC, Seurat v3.0, LIGER, and Harmony. Each dot in the boxplot denotes one cell type. **g** Improvement in accuracy of imputed protein level. Each dot represents a protein. *X*-axis is the Pearson correlation between the ground truth protein level and the RNA level of its coding gene. *Y*-axis is the Pearson correlation between the ground truth protein level and bindSC imputed protein level. **h** Comparison of the epitope abundance of CD19, CD14, and CD11c (*x*-axes) with the RNA expression levels of their coding genes (i.e., *CD19*, *CD14*, and *ITGAX*; *y*-axes; first row) and with the bindSC imputed protein levels (*y*-axes; second row). **i** Gene-protein network inferred from Pearson correlation between genes and bindSC inferred protein levels. A cutoff of 0.55 is used and top five highly correlated genes of each protein are kept

We used a CITE-seq dataset consisting of 30,672 human bone marrow cells with a panel of 25 proteins [20]. Unsupervised clustering of the RNA profiles revealed cell types largely consistent with those in the protein profiles, except for some noticeable differences (Fig. 3a, b). CD8+ and CD4+ T cells were partly blended together in the RNA data (ARI = 0.43) but separated clearly in the protein data (ARI = 0.82). On the other hand, conventional dendritic cells (cDC2) were separated from other clusters in the RNA profiles but were intermixed with other cell types in the protein profile. In contrast, the gene expression levels of the 25 RNAs encoding the 25 proteins lacked delineating power and could not yield meaningful classification (ARI = 0.09; Fig. 3c). We randomized the orders of the cells in the RNA matrix and the protein matrix, then tested the ability of each method in generating meaningful co-embeddings and recovering the correct pairing. Seurat v3.0, LIGER and Harmony, which work with only data matrix of 25 homologous features, failed to produce meaningful co-embeddings (Additional file 1: Fig. S8a): the cells from the protein data were well clustered, but those from the RNA data were not meaningfully clustered.

We then tested bindSC on this task. The matrix **X** was set as the protein matrix, **Y** the RNA matrix of 3000 highly variable genes, and **Z** the RNA matrix containing only the 25 protein-homologous genes. Remarkably, the majority of the cells from the two modalities became well aligned in the co-embedding (Fig. 3d, e). Notably, the bulk of CD4+ and CD8+ T cells mixing together in the RNA data became well separated in the co-embedding. We calculated the label transfer accuracy (Methods) between the protein and the RNA cells deriving from the same original cells in the co-embedding. The overall label transfer accuracy for bindSC was significantly higher than those obtained by Seurat, LIGER, and Harmony (Fig. 3f). Overall, the protein levels imputed by bindSC from the entire set of RNAs (i.e., the modality fusion matrix **Z**) showed consistently higher correlation with the measured epitope levels than the homologous RNA expression levels, indicating meaningful inference of post-transcriptional regulation (Fig. 3g). For example, protein levels for CD19, CD14, and CD11c, markers overexpressing on B cells, monocytes, and DCs, are not highly correlated with the observed RNA expression levels in the same cells (Fig. 3h), however, had much higher correlation with the levels imputed by bindSC from the whole set of RNA expressions. The imputed profile has high correlation with the true protein levels (Pearson’s *R* = 0.6) and low correlation with the initial gene scores (Pearson’s *R* < 0.3) (Additional file 1: Fig. S8d), again indicating the power of associating two modalities de novo. We then used the modality fusion matrix **Z** to infer a gene-protein correlation network (Fig. 3i and Additional file 1: Fig. S9, Methods), in which we see canonical RNA-protein interaction modules centering around CD14 (*CD14*) and CD79b (*CD79B*), respectively. Other proteins such as CD19 (*CD74*, *MS4A1*, etc.) and CD11a/CD11c (*LYZ* etc.) have stronger correlation with the RNAs of their upstream or downstream genes, rather than the RNAs of their own coding genes. This result demonstrates the power of bindSC in discovering biologically meaningful regulatory relations and pathways through scRNA-seq and mass cytometry data integration.

#### Integration of scRNA-seq with CyTOF data revealing activated CAR-NK cells

To further understand the utility of bindSC, we applied it to integrate scRNA-seq and CyTOF data generated from an immunotherapy study. Chimeric antigen receptor (CAR)-transduced natural killer (NK) cells have demonstrated promising efficacy and safety in killing cells in CD19-positive lymphoid tumors [46]. To understand why certain NK cells are more effective than others, we compared the molecular profiles of three groups of NK cells: (1) wildtype non-transduced (NT-NK), (2) transduced with CD19CAR, and (3) transduced with interleukin-15 (IL15).

**Fig. 4:**
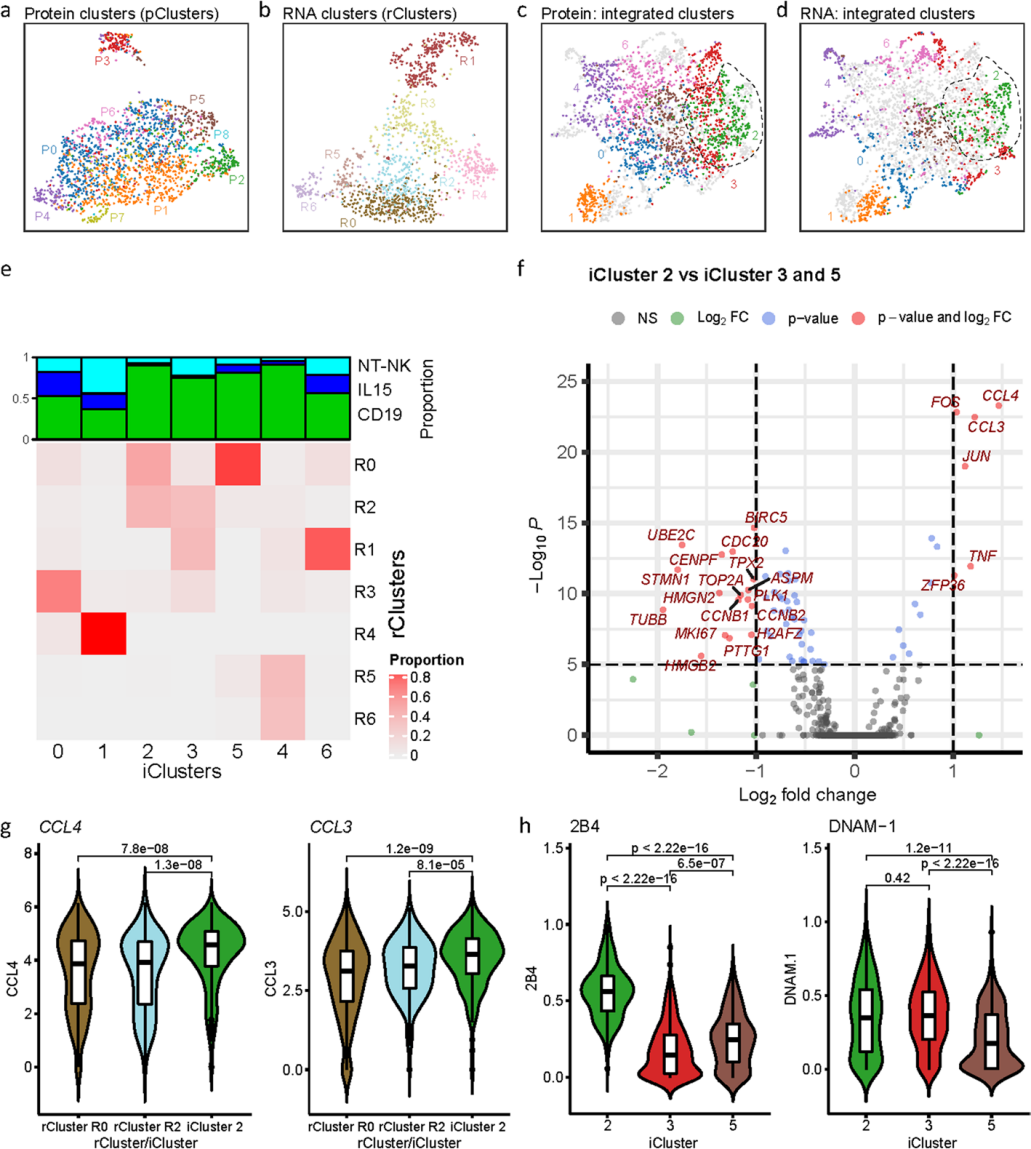
Integration of CyTOF and scRNA data of CD19-CAR NK, IL15 NK, and NT-NK cells. **a**, **b** Cells from CD19-CAR NK, IL15 NK, and NT-NK products, clustered independently by CyTOF (**a**) and scRNA (**b**) data. There is no correspondence between protein clusters (pClusters) and RNA clusters (rClusters). **c**, **d** Integrated clusters (iClusters) after running bindSC on the CyTOF and the scRNA data. CyTOF (**a**) and scRNA-seq (**b**) are emphasized, respectively, for better visualization. Cells in the non-emphasized modality are shown in light gray. iCluster 2 is circled out by dashed lines. **e** Correspondence of iClusters and rClusters. The colors denote the proportion of iCluster in each protein cluster, normalized by each column. The top annotation shows frequencies of three cell groups (cyan: NT-NK, blue: IL15, green: CD19) in each iCluster. **f** Differentially expressed (Wilcoxon test) genes between iCluster 2 and iClusters 3 and 4. Highlighted genes are known NK cell activation (upregulated). **g** Gene expression levels of *CCL4* and *CCL3* in iCluster 2 and rClusters R0 and R2. The *p* values shown are from the Wilcoxon test. **h** Protein expression levels of *2B4* and *DNAM-1* in iCluster 2, 3, and 5. The *p* values shown are from the Wilcoxon test

We obtained scRNA-seq data (1341 cells × 33,538 genes) and CyTOF data (2000 cells × 29 proteins) from the three groups. Clustering the CyTOF and scRNA-seq data by themselves revealed nine and seven clusters (called rClusters and pClusters hereafter), respectively (Fig. 4a, b). After performing bindSC integration, seven integrated clusters (iClusters) were revealed (Fig. 4c, d). Notably, portions of the rClusters R0 and R2, deriving from a subset of CD19CAR NK cells, were reassigned to iCluster 2 (Fig. 4e). Differential expression analysis shows that scRNA-seq cells assigning to iCluster 2 express significantly higher level of inflammation marker *TNF*, cytokine genes *CCL4* and *CCL3*, and TF genes including *JUN* and *FOS*, all indicating activation [47] (Fig. 4g and Additional file 1: Fig. S10). Meanwhile, CyTOF cells assigning to iCluster 2 showed significantly higher levels of *2B4* and *DNAM-1* expressions (Fig. 4h), also indicating activation [48]. Importantly, this subset of cells can be identified from neither the scRNA-seq clusters (Fig. 4g), nor the CyTOF clusters alone (Additional file 1: Fig. S11). Thus, integrating scRNA-seq and CyTOF data using bindSC led to the discovery of a subset of highly activated CD19CAR NK cells. This finding may help quantify the therapeutic value of a CAR-NK cell project and reveal mechanisms that can be further leveraged to improve the efficacy of the treatment.

#### Integration of sci-ATAC-seq and sci-RNA-seq data revealing true identities of rare fetal cells

**Fig. 5:**
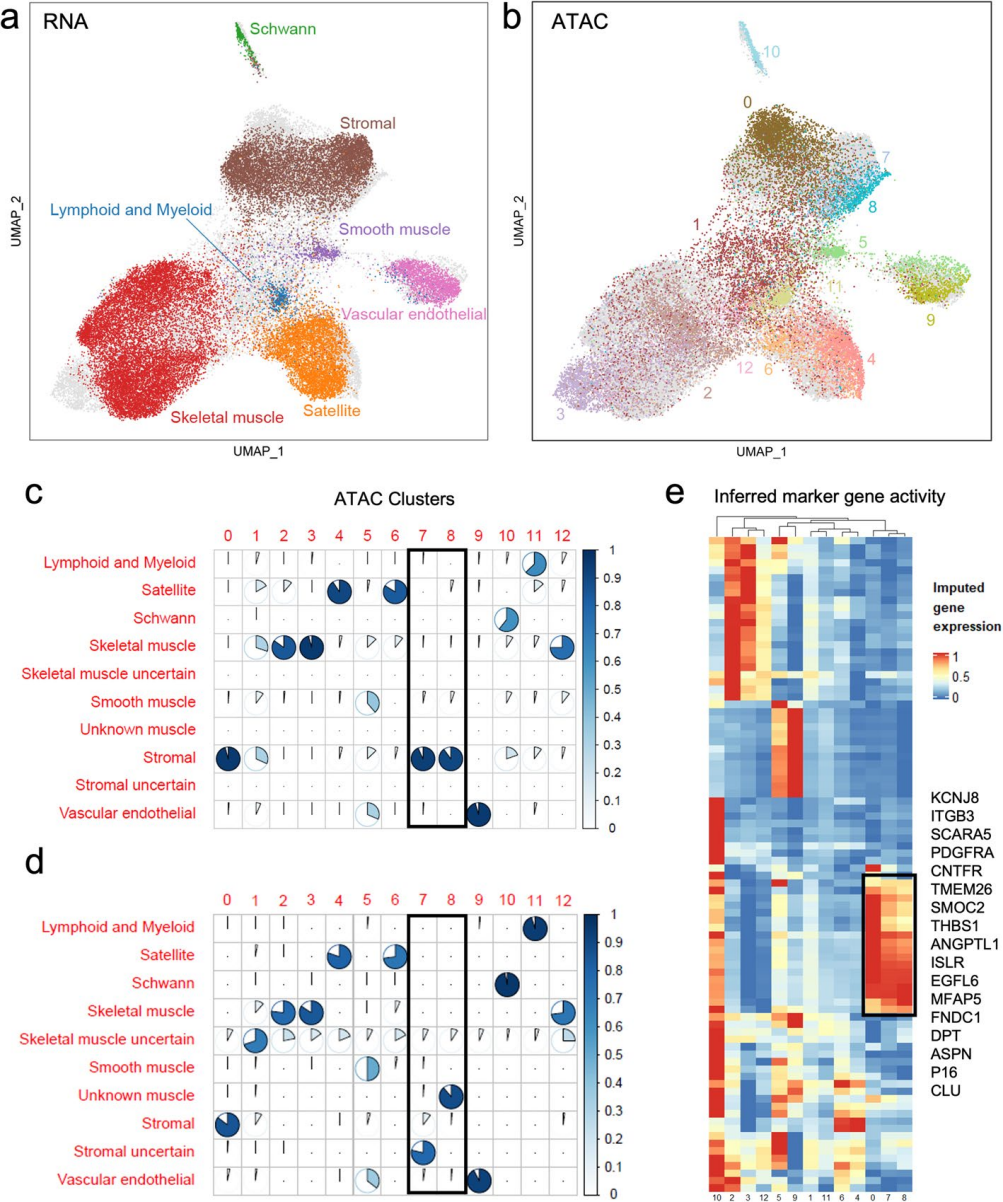
Results for fetal muscle sci-RNA-seq and sci-ATAC-seq data integration. **a**, **b** UMAPs generated from bindSC co-embedding of the sci-RNA (**a**) and the sci-ATAC (**b**) data. Dots and labels are colored synchronously by cell type. Gray dots represent cells from the other omics (i.e., ATAC cells in **a** and RNA cells in **b**). **c**, **d** Cell types identified respectively using bindSC (**c**) and based on gene activity scores in the original publication (**d**). Clusters 7 and 8, which are classified differently by bindSC, are highlighted by black boxes. **e** DEGs for each cluster. DEGs specific for clusters 0, 7, and 8 are highlighted by a black box

Bi-CCA alignment may also help identify rare cell populations that are hard to identify in one modality. Recent study used sci-ATAC-seq3 technology to generate the chromatin accessibility profile of ~800,000 human fetal cell atlas from 15 organs [5]. The types of cells in the sci-ATAC-seq data can be annotated by matching clusters with those in the sci-RNA-seq data (Additional file 1: Fig. S12a-b). However, this approach requires good alignment between sci-RNA-seq and sci-ATAC-seq clusters, which is challenging to acquire for rare cell types of limited number of cells. Thus, additional manual review and examination of marker gene expressions are likely required to ensure accurate annotation result. For example, the fetal muscle cell ATAC dataset, consisting of 27,181 cells, has a cluster of cells (3.55% abundance) labeled as unknown (Additional file 1: Fig. S12b), using the above annotation strategy based on gene activity score (ATAC peaks collapsed to genes based on genomic proximity) matrix in the original study. After integrating the sci-ATAC-seq and the sci-RNA-seq data using bindSC, we obtained joint ATAC and RNA profiles (Fig. 5a, b), in which clusters 7 and 8 were annotated as stromal cells (Fig. 5c), different from the previously reported ones (Fig. 5d). We then performed pathway enrichment analysis based on the differentially expressed genes (DEGs) in this cluster (Fig. 5e) and found that these genes are significantly associated with immune (*p* = 0.003), vascular (*p* = 0.012), placenta (*p* = 0.010), and adipose (*p* = 0.005), indicating that these clusters are highly likely stroma cells surrounding muscle cells. The DEGs are also enriched in biological processes related to extracellular matrix organization (*p* < 10^−4^), regulation of exocytosis (*p* < 10^−4^) and platelet degranulation (*p* < 10^−4^). In comparison, gene activity scores only indicated moderate similarity between clusters 0, 7, and 8, but failed to cluster them together in unsupervised hierarchical clustering (Additional file 1: Fig. S12c-d).

To examine bindSC’s scalability in large-scale datasets, we created ten benchmark datasets with cells number ranging from 22,552 to 834,424 by resampling cells in the fetal muscle atlas (Additional file 1: Fig. S12e). The block size was set to 1000 for bindSC in each dataset. We obtained the elapsed run time and maximum memory for all the benchmarks using one thread (with a 28-core Intel Skylake CPU@2.6GHz). As expected, bindSC runtime appeared linear to the number of cells, ranging from 4 min for analyzing 23,000 cells to 184 min for 800,000 cells. The maximum memory usage was <10GB in all the datasets, regardless of cell numbers.

## Discussion

Despite the ground-breaking advances in single-cell technologies including multiomics technologies, there always exists a need to computationally integrate multiple data matrices of different modalities sampled from the same biological population to derive a more comprehensive characterization of cellular identities and functions.

Our method bi-CCA and an associated tool bindSC have addressed this important analytical challenge without compromising biological complexity in the data. In our experiments, bindSC successfully integrated data obtained from a wide variety of vastly different technologies covering transcriptomes, epigenomes, and proteomes and clearly outperformed existing tools such as Seurat v3.0, LIGER and Harmony, when being evaluated objectively using true single-cell multiomics data derived from the same cells with broad parameter settings (Additional file 1: Figs. S4i and S8e). In particular, Seurat v3.0, LIGER, and Harmony are essentially first-order solutions that can be applied to only rows or columns. Our approach can further improve integration performance by leveraging distal regulatory relations [8] (Fig. 2g; Additional file 1: Figs. S8d and S13), as exemplified in the interaction between *Nfib* and a 1Mbps upstream putative enhancer site discovered by bindSC. Other scATAC-seq analysis pipelines such as MAESTRO [19], Cicero [26], and ArchR [49] can consider distal interactions, but only via co-accessibility patterns within scATAC-seq profiles. However, gene activity scores generated by them did not improve integration results in our benchmarking experiments (Additional file 2: Supplementary Note 5 and Additional file 1: Fig. S16). This highlights the challenge of performing de novo gene regulation network inference from scATAC-seq data. On the other hand, bindSC outperforms cell-cell similarity-based methods including MMD-MA, UnionCom, SCOT, and Pamona, which align cells based on manifolds only and do not explicitly model feature interactions (Additional file 1: Figs. S2, S4 and S8). Collectively, the ability to obtain de novo alignment between both cells and features enables simultaneous discovery of novel cell populations and associated multimodal features.

Similarly, bindSC was able to meaningfully associate expression levels of mRNAs with those of surface proteins, a very challenging task due to complexity in post-transcriptional modification. The resulting co-embedding offered deeper biological insights than embeddings derived from single modalities or by using existing integrative approaches. For example, RNA-protein relationships specific to monocytes and B cells were found de novo by integrating RNA and protein expression data obtained from bone marrow samples. A hyperactive subset of CAR-NK cells was found by integrating scRNA-seq data with CyTOF data.

In addition, bindSC can potentially be applied to integrate single-cell sequencing data with spatially resolved molecular profiling data, such as 10x scRNA-seq with multiplexed error-robust fluorescence in situ hybridization data (MERFISH), in which feature dimensions are different between two modalities. The generic framework of bi-CCA also makes it possible to align multiple datasets acquired from more than 2 modalities, for example, aligning scATAC-seq data with scRNA-seq data and subsequently with spatial transcriptomics data. Although further experimentation is clearly required, the clean definition of CCA may warrant relatively straightforward interpretation of the complex integration results.

Bi-CCA made two assumptions: (1) the two sets of cells are sampled uniformly from the same biological sample and (2) the features of the two datasets are linearly correlated. These two assumptions are met under many scenarios of current investigations, however, could be violated when there are insufficient number of cells obtained from a rapidly developing cell population. In addition, although we did not observe obviously mismatched clusters because most datasets we studied are derived from biological samples of limited heterogeneity, it is possible to observe modality-specific clusters that cannot be well aligned by bi-CCA. That means the two modalities may not have evenly represented molecular heterogeneity in the sample, violating the second assumption. Identifying and interpreting those data will require in-depth analyses from both biological and technical perspectives. Consequently, the accuracy of the co-embedding could vary, depending on sampling density and complexity of the population. We measured accuracy with respect to data complexity in the simulation experiments (Additional file 1: Fig. S2); however, accuracy on a real dataset could be complex to gauge a priori and will require case by case investigation in the context of a specific study, followed by necessary experimental validation. Nonetheless, in this study, we clearly proved based on objective ground truth data that bi-CCA substantially avoided biases introduced by existing methods and that bindSC is a robust implementation that can be applied to derive meaningful results on most recent datasets containing thousands to tens of thousands of cells (Additional file 3: Table S1).

BindSC is efficiently implemented in R with a low memory footprint and fast convergence speed, e.g., <15 iterations, 10 min (Additional file 1: Figs. S4c, S8c and S15). The major computational cost for bindSC is from calculating cell/feature co-embedding coordinates using singular value decomposition (SVD) (Methods). It typically requires *O*(*MNL*) floating-point operations to construct *MN* cell-cell distance matrix as input to SVD decomposition, where *M* and *N* are cell numbers of the two modalities and *L* is the number of overlapped features. To address this computational challenge, bindSC implements the “divide-and-conquer eigenvalue algorithm”. The divide part first splits cells into different blocks specified by users, which can be solved in parallel with lower memory usage (Additional file 1: Fig. S1b). The conquer part then merges results from each block recursively. Therefore, the maximal memory usage of bindSC is independent of the total cell number (Additional file 1: Fig. S12e).

## Conclusions

Taken together, we believe that bindSC is likely the first tool that has achieved de novo bi-order integration of data matrices generated by different technologies and can be applied in broad settings. In the single-cell domain, bindSC can clearly be applied to align cells and features simultaneously, which are important for ongoing investigations in the Human Cell Atlas [50], the NIH HubMap [51], the Human Tumor Cell Network [52], and on remodeling of tumor microenvironment [53]. Further, bindSC can potentially be applied to other domains, such as integrating patient sample mRNA profiles with cell-line drug-sensitivity data [54].

## Methods

### BindSC workflow

BindSC workflow for creating in silico single-cell multi-omics embeddings consists of four steps:

1. Individual dataset preprocessing including variable feature selection and cell clustering
2. Initializing feature matching across modalities (i.e., constructing modality fusion matrix)
3. Identifying cell correspondence using the bi-cca algorithm
4. Jointly clustering cells between two modalities in the co-embedding latent space and constructing multi-omics profiles for various downstream analysis.

We formulate our method for the case of two modalities. Let ***X ∈*** *ℝ*^*M* × *K*^ be a single-cell dataset of features *g*_1_, *g*_1_, ⋯, *g*_*M*_ by cells *c*_1_, *c*_1_, ⋯, *c*_*K*_ and ***Y ∈*** *ℝ*^*N* × *L*^ be a single-cell dataset of feature *p*_1_, *p*_2_, ⋯, *p*_*N*_ by cells *d*_1_, *d*_1_, ⋯, *d*_*L*_. *M* and *N* are the numbers of features (e.g., gene expression, chromatin accessibility, protein abundance level) in the two datasets, and *K* and *L* are the numbers of cells. Using integrating genes with ATAC peaks as an example, *g*_1_, ⋯, *g*_*M*_ represent the gene expression levels and *p*_1_, …, *p*_*N*_ represent the ATAC peaks, with *M* ≤ *N*.

It is worth noting that mathematically, ***Z*** may be defined in two ways depending on which modality is used as ***X***. However, because ***Z*** is the predicted features in ***X*** for cells in ***Y***, the process is usually only meaningful in one way. Biologically, it is meaningful to predict gene expression from ATAC peaks (i.e., ***X***: RNA, ***Y*:** ATAC, ***Z***: ATAC cells × genes), or proteins from the RNA expression profile (i.e., ***X***: protein, ***Y***: RNA, ***Z***: RNA cells × proteins), but not the other way around. In addition, it is more stable to project more features to fewer, which is consistent with the above notions.

The important component of each step is described as follows.

#### Individual modality preprocessing

For each modality, we follow their standard processing pipelines, which usually include variable feature selection and unsupervised cell clustering. The cluster information derived from all modalities is used for downstream parameter optimization.

#### Initializing feature matching across modalities

Because features in the two datasets are generally different, bindSC requires an additional modality fusion matrix ***Z*** ∈*ℝ*^*M* × *L*^ to bridge ***X*** and ***Y***. The modality fusion matrix ***Z*** can be considered as the projection of ***Y*** to the feature space of ***X***. Taking the integration of scRNA-seq and scATAC-seq as an example, ***Z*** can be derived from scATAC-seq profiles by summing reads in gene bodies [20, 22, 26] and is commonly referred to as a gene score matrix. In bi-CCA, ***Z*** is updated iteratively. In the following text, the initial value of ***Z*** is denoted by ***Z***^(**0**)^. In addition, for scRNA-seq and scATAC-seq data, ***Z***^(**0**)^ can be inferred differently using the regulatory potential (RP) model in MAESTRO [19], or the co-accessibility model in Cicero (Additional file 1: Fig. S16). Users can select proper ***Z***^(**0**)^ based on the three metrics of integration mentioned in Parameter optimization below.

#### Bi-order canonical correlation analysis (Bi-CCA)

The key algorithm implemented in bindSC is bi-CCA, the concept of which extends traditional CCA [20, 27, 55] to both rows and columns to enable capturing of correlated variables in cells and features simultaneously. Bi-CCA introduces two cell-level projection matrices ***U ∈*** *ℝ*^*K* × *E*^, ***S ∈*** *ℝ*^*L* × *E*^ such that the correlations between indices ***XU*** and ***ZS*** are maximized, and two feature-level projection matrices ***T ∈*** *ℝ*^*M* × *E*^, ***V ∈*** *ℝ*^*N* × *E*^ such that the correlations between indices ***Z***^′^***T*** and ***Y***^′^***V*** are maximized. *E* is the dimensionality of the latent space, which is empirically set to the number of principle components (PCs) as in other analyses. The general optimization framework can be formulated as follws:

1 $$\underset{\boldsymbol{U},\boldsymbol{S},\boldsymbol{T},\boldsymbol{V},\boldsymbol{Z}}{\mathrm{argmax}}\boldsymbol{tr}\left\{{\left(\boldsymbol{XU}\right)}^{\prime}\boldsymbol{ZS}+{\left({\boldsymbol{Z}}^{\prime}\boldsymbol{T}\right)}^{\prime }\ {\boldsymbol{Y}}^{\prime}\boldsymbol{V}\right\}$$

subject to ***U***^′^***U*** = ***I***, ***S***^′^***S*** = ***I***, ***T***^′^***T*** = ***I***, ***V***^′^***V*** = ***I***.

If the modality fusion matrix ***Z*** was known, the objective (1) would be divided into two disjoint traditional canonical correlation analysis (CCA) problems. The left term identifies cells of similar (aligned) features, while the right term identifies features shared by the (aligned) cells.

Given that Eq. (1) is a multi-objective optimization problem, we design the following weighted optimization to balance the importance of each modality.

2 $${\displaystyle \begin{array}{c}\underset{\boldsymbol{U},\boldsymbol{S},\boldsymbol{T},\boldsymbol{V},\boldsymbol{Z}}{\mathrm{argmax}}\boldsymbol{tr}\left\{\frac{1-\uplambda}{n_l}{\left(\boldsymbol{XU}\right)}^{\prime}\boldsymbol{ZS}+\frac{\uplambda}{n_{\boldsymbol{r}}}{\left({\boldsymbol{Z}}^{\prime}\boldsymbol{T}\right)}^{\prime }\ {\boldsymbol{Y}}^{\prime}\boldsymbol{V}\right\},\end{array}}$$

in which ${n}_l={\left\Vert \boldsymbol{X}{\boldsymbol{U}}^{\left(\mathbf{0}\right)}{\boldsymbol{S}}^{\left(\mathbf{0}\right)\prime}\right\Vert}_F^2$ and ${n}_r={\left\Vert {\boldsymbol{T}}^{\left(\mathbf{0}\right)}{\boldsymbol{V}}^{\left(\mathbf{0}\right)}\prime \boldsymbol{Y}\right\Vert}_F^2$ represent scale factors for two objectives ***U***^(**0**)^**,*S***^(**0**)^ are CCVs of (***X***, ***Z***^(**0**)^) and ***T***^(**0**)^**,*V***^(**0**)^ are CCVs of (***Y***, ***Z***^(**0**)^). The scale factor λ is introduced to balance the importance of each modality and it ranges from 0 to 1. Equivalently, λ balances the relative importance of cells and features because one modality corresponds to cells and the other features. We also add a penalty term 0 ≤ α < 1, which measures the contribution of the initial ***Z***^(**0**)^ on final integration. The final objective function is thus:

3 $${\displaystyle \begin{array}{c}\underset{\boldsymbol{U},\boldsymbol{S},\boldsymbol{T},\boldsymbol{V},\boldsymbol{Z}}{\mathrm{argmax}}\ \left(1-\upalpha \right)\boldsymbol{tr}\left\{\frac{1-\uplambda}{n_l}{\left(\boldsymbol{XU}\right)}^{\prime}\boldsymbol{ZS}+{\frac{\uplambda}{n_{\boldsymbol{r}}}}^{\prime }{\left(\boldsymbol{ZT}\right)}^{\prime }\ {\boldsymbol{Y}}^{\prime}\boldsymbol{V}\right\}-\upalpha {\left\Vert \boldsymbol{Z}-{\boldsymbol{Z}}^{\left(\mathbf{0}\right)}\right\Vert}_F^2,\end{array}}$$

subject to ***U***^′^***U*** = ***I***, ***S***^′^***S*** = ***I***, ***T***^′^***T*** = ***I***, ***V***^′^***V*** = ***I***, $${\left\Vert \boldsymbol{Z}\right\Vert}_F^2=1$$

To solve Eq. (3), we also standardize ***Z***^(**0**)^ to let ${\boldsymbol{Z}}^{\left(\mathbf{0}\right)}:= {\boldsymbol{Z}}^{\left(\mathbf{0}\right)}/{\left\Vert {\boldsymbol{Z}}^{\left(\mathbf{0}\right)}\right\Vert}_F^2$, and initialized with ***Z ≔ Z***^(**0**)^. The standard singular value decomposition (SVD) can be implemented to obtain the canonical correlation vectors (CCVs) (Algorithm 1) at cell levels to approximate CCVs for the left term:

4 $${\displaystyle \begin{array}{c}\left(\boldsymbol{U},\boldsymbol{S}\right):= \underset{\boldsymbol{U},\boldsymbol{S}}{\mathrm{argmax}}\boldsymbol{tr}\left\{{\boldsymbol{U}}^{\prime }{\boldsymbol{X}}^{\prime}\boldsymbol{ZS}\right\}\mathrm{subject}\ \mathrm{to}\ {\boldsymbol{U}}^{\prime}\boldsymbol{U}=\boldsymbol{I},{\boldsymbol{S}}^{\prime}\boldsymbol{S}=\boldsymbol{I},\end{array}}$$

and for the right term:

5 $$\left(\boldsymbol{T},\boldsymbol{V}\right):= \underset{\boldsymbol{T},\boldsymbol{V}}{\mathrm{argmax}}\ \boldsymbol{tr}\left\{{\left(\boldsymbol{ZT}\right)}^{\prime }\ {\boldsymbol{Y}}^{\prime}\boldsymbol{V}\right\}=\underset{\boldsymbol{T},\boldsymbol{V}}{\mathrm{argmax}}\ \boldsymbol{tr}\left\{{\boldsymbol{T}}^{\prime}\boldsymbol{Z}{\boldsymbol{Y}}^{\prime}\boldsymbol{V}\right\}\ \mathrm{subject}\ \mathrm{to}\ {\boldsymbol{T}}^{\prime}\boldsymbol{T}=\boldsymbol{I},{\boldsymbol{V}}^{\prime}\boldsymbol{V}=\boldsymbol{I}.$$

Once CCV pairs (***U***, ***S***) and (***T***, ***V***) are obtained, the modality fusion matrix ***Z*** can be updated as follows:

6 $$\left(\boldsymbol{Z}\right):= \left(1-\upalpha \right)\left\{\frac{1-\uplambda}{n_l}\boldsymbol{XU}{\boldsymbol{S}}^{\prime }+\frac{\uplambda}{n_{\boldsymbol{r}}}\boldsymbol{T}{\boldsymbol{V}}^{\prime}\boldsymbol{Y}\right\}+{\upalpha \boldsymbol{Z}}^{\left(\mathbf{0}\right)}.$$

In Eq. (6), the updated modality fusion matrix ***Z*** is composed of three parts: (1) ***Z*** reconstructed from the first modality, (2) ***Z*** reconstructed from the second modality, and (3) the initial modality fusion matrix ***Z***^(**0**)^. Given ${n}_l\to {\left\Vert \boldsymbol{XU}{\boldsymbol{S}}^{\prime}\right\Vert}_F^2$**,**${n}_r\to {\left\Vert \boldsymbol{T}{\boldsymbol{V}}^{\prime}\boldsymbol{Y}\right\Vert}_F^2$, ${\left\Vert {\boldsymbol{Z}}^{\left(\mathbf{0}\right)}\right\Vert}_F^2=1$, the contributions of the three terms on updated matrix ***Z*** are (1 − *λ*)(1 − *α*), (1 − *α*) and *α*, respectively. Next, we set

7 $$\left(\boldsymbol{Z}\right):= \boldsymbol{Z}/{\left\Vert \boldsymbol{Z}\right\Vert}_F^2.$$

The update process (4) ~ (7) are repeated until it reaches convergence. Because each of the subproblems is convex with respect to the block variables being optimized, the algorithm is guaranteed to converge to a fixed point (local minimum).

#### Fast integration in the low-dimension space

The feature dimensionality of the matrix ***Y*** is usually more than 100,000 for single-cell epigenetic profiles, which will take longer time/larger memory for integration. In addition, the single-cell epigenetic profiles are usually sparse and noisy. Therefore, we present a modified version of bi-CCA, which takes low-dimension profiles rather than original matrices on integrating high-dimension datasets. We first perform CCA on matrix pair (***X***, ***Z***^(**0**)^) to derive the low-dimension embeddings as (***P***_***X***_, ***P***_***z*0**_) (Algorithm 1) and then perform dimension reduction on original matrix ***Y*** to derive the low-dimension embeddings as ***P***_***Y***_. Then, the updated matrix ***P***_***z***_ could be solved based on the following equation:

8 $$\underset{\boldsymbol{U},\boldsymbol{S},\boldsymbol{T},\boldsymbol{V},\boldsymbol{Z}}{\mathrm{argmax}}\ \left(1-\upalpha \right)\boldsymbol{tr}\left\{\frac{1-\uplambda}{n_l}\Big({\left({\boldsymbol{P}}_{\boldsymbol{X}}\boldsymbol{U}\right)}^{\prime }{\boldsymbol{P}}_{\boldsymbol{z}}\ \boldsymbol{S}+\frac{\uplambda}{n_r}\ {\boldsymbol{T}}^{\prime }{\boldsymbol{P}}_{\boldsymbol{z}}\ {\boldsymbol{P}}_{\boldsymbol{Y}}^{\prime}\boldsymbol{V}\right\}-\upalpha {\left\Vert {\boldsymbol{P}}_{\boldsymbol{z}}-{\boldsymbol{P}}_{\boldsymbol{z}\mathbf{0}}\right\Vert}_F^2,$$

subject to ${\boldsymbol{U}}^{\prime}\boldsymbol{U}=\boldsymbol{I},{\boldsymbol{S}}^{\prime}\boldsymbol{S}=\boldsymbol{I},{\boldsymbol{T}}^{\prime}\boldsymbol{T}=\boldsymbol{I},{\boldsymbol{V}}^{\prime}\boldsymbol{V}=\boldsymbol{I},{\left\Vert {\boldsymbol{P}}_{\boldsymbol{z}}\right\Vert}_F^2=1$,in which ${n}_l={\left\Vert {\boldsymbol{P}}_{\boldsymbol{X}}\boldsymbol{U}{\boldsymbol{S}}^{\prime}\right\Vert}_F^2$ and ${n}_r={\left\Vert \boldsymbol{T}{\boldsymbol{V}}^{\prime }{\boldsymbol{P}}_{\boldsymbol{Y}}\right\Vert}_F^2$ represent scale factors for two objectives, and ***P***_***z*0**_ is normalized as ${\boldsymbol{P}}_{\boldsymbol{z}\mathbf{0}}:= {\boldsymbol{P}}_{\boldsymbol{z}\mathbf{0}}/{\left\Vert {\boldsymbol{P}}_{\boldsymbol{z}\mathbf{0}}\right\Vert}_F^2$. After performing the similar iteration process as described in equation 6, we calculate the imputed ***Z*** as follows:

9 $$\left(\boldsymbol{Z}\right):= \left(1-\upalpha \right)\left\{\frac{1-\uplambda}{n_l}\boldsymbol{XU}\ {\boldsymbol{S}}^{\prime }+\frac{\uplambda}{n_{\boldsymbol{r}}}\boldsymbol{T}{\boldsymbol{V}}^{\prime}\boldsymbol{Y}\right\}+\upalpha {\boldsymbol{Z}}^{\left(\mathbf{0}\right)}.$$

### Jointly clustering cells across datasets in shared latent space and constructing joint multiomics profiles

Equation (4) projects cells of two datasets into a correlated *E*-dimensional space with cell coordinates ***U =*** (***u***_1_, ***u***_2_, …, ***u***_*K*_) and ***S =*** (***s***_1_, ***s***_2_, …, ***s***_*L*_), respectively. L2-normalization is performed to remove global differences in scale, therefore

10 $${\displaystyle \begin{array}{c}{\hat{\boldsymbol{u}}}_i={\boldsymbol{u}}_i/{\left\Vert {\boldsymbol{u}}_i\right\Vert}_2,i=1,2,\dots, K,\\ {}{\hat{\boldsymbol{s}}}_i={\boldsymbol{s}}_i/{\left\Vert {\boldsymbol{s}}_i\right\Vert}_2,i=1,2,\dots, L.\end{array}}$$

The shared nearest neighbor (SNN) graph is constructed by calculating the *l*-nearest neighbors (20 by default) based on the Euclidean distance in the L2-normlized space. The fraction of shared nearest neighbors between the cell and its neighbors is used as the weights of the SNN graph. Leiden algorithm [56] is used to group cells into interconnected clusters (termed meta-cluster) based on constructed SNN graph with a resolution parameter set by users (default 0.5).

We can understand the molecular-level interaction among modalities by associating the modality fusion matrix ***Z ∈*** *ℝ*^*M* × *L*^ with ***Y*** directly, which are measured in the matched cell population.

### Label transfer between modalities

Co-embeddings ***U***, ***V*** are used to conduct cell type label transfer. A support vector machine (SVM) model (*svm* function in R package e1071) was trained with cell coordinates ***U*** and their corresponding cell type from the first modality as input. A normalized cell-type score (ranges from 0 to 1 and sums up to 1) for each cell is returned. Cells are classified as the type achieving the highest score.

To assess the accuracy of label transfer on cell types, we first build a confusion matrix ***C*** with element *C*_*i*, *j*_ representing the number of cells of type *i* predicted as type *j*. The matrix is then normalized by rows (so that each row sums up to 1). The cell type label transfer accuracy is the percentage of correct prediction. We average label transfer accuracy across all cell types to obtain an overall accuracy.

## Algorithm 1. Calculating CCVs using SVD

Take a subproblem from Eq. (4) as an example, the goal of this module is to find projection matrix ***U ∈*** *ℝ*^*K* × *E*^ and ***S ∈*** *ℝ*^*L* × *E*^ such that the correlations between two indices ***XU*** and ***ZS*** are maximized.

11 $$\underset{\boldsymbol{U},\boldsymbol{S}}{\mathrm{argmax}}\ \boldsymbol{tr}\left({\boldsymbol{U}}^{\prime }\ {\boldsymbol{X}}^{\prime}\boldsymbol{ZS}\right)\ \mathrm{subject}\ \mathrm{to}\kern0.50em {\boldsymbol{U}}^{\prime}\boldsymbol{U}=\boldsymbol{I},{\boldsymbol{S}}^{\prime}\boldsymbol{S}=\boldsymbol{I}.$$

We define ${\Sigma}_{{\boldsymbol{X}}^{\prime}\boldsymbol{Z}}:= {\boldsymbol{X}}^{\prime}\boldsymbol{Z}$. By letting ***U ∈*** *ℝ*^*K* × *E*^ and ***S*** ∈ *ℝ*^*K* × *E*^ be the matrices of the first *E* left- and right singular vectors of ${\Sigma}_{{\boldsymbol{X}}^{\prime}\boldsymbol{Z}}$, the optimum in Eq. (11) is solved with a direct analogy of Eq. (6). *E* represents the number of singular vectors in the latent space, a user-definable parameter that can be further optimized (detailed in Parameter optimization).

## Algorithm 2. Updating modality fusion matrix ***Z***

This algorithm is used to solve ***Z*** in Eq. (3), assuming that CCV pairs (***U***, ***S***) and (***T***, ***V***) are obtained. We denote the objective function as

12 $$f\left(\boldsymbol{Z}\right)=\left(1-\upalpha \right)\boldsymbol{tr}\left\{\frac{1-\uplambda}{n_l}{\left(\boldsymbol{XU}\right)}^{\prime}\boldsymbol{ZS}+\frac{\uplambda}{n_{\boldsymbol{r}}}{\left({\boldsymbol{Z}}^{\prime}\boldsymbol{T}\right)}^{\prime }\ {\boldsymbol{Y}}^{\prime}\boldsymbol{V}\right\}-\upalpha {\left\Vert \boldsymbol{Z}-{\boldsymbol{Z}}^{\left(\mathbf{0}\right)}\right\Vert}_F^2.$$

Therefore,

13 $$\nabla f\left(\boldsymbol{Z}\right)=\left(1-\upalpha \right)\left\{\frac{1-\uplambda}{n_l}\boldsymbol{XU}\ {\boldsymbol{S}}^{\prime }+\frac{\uplambda}{n_{\boldsymbol{r}}}\boldsymbol{T}{\boldsymbol{V}}^{\prime}\boldsymbol{Y}\right\}-\upalpha \left(\boldsymbol{Z}-{\boldsymbol{Z}}^{\left(\mathbf{0}\right)}\right)$$

Equation (12) is maximized when ∇*f*(***Z***) = 0. Therefore, we can update ***Z*** as

14 $$\left(\boldsymbol{Z}\right):= \left(1-\upalpha \right)\left\{\frac{1-\uplambda}{n_l}\boldsymbol{XU}\ {\boldsymbol{S}}^{\prime }+\frac{\uplambda}{n_{\boldsymbol{r}}}\boldsymbol{T}{\boldsymbol{V}}^{\prime}\boldsymbol{Y}\right\}+{\upalpha \boldsymbol{Z}}^{\left(\mathbf{0}\right)}.$$

### Parameter optimization

There are three key hyperparameters when running bindSC for integration. The dimensionality *E* in the latent space, the couple coefficient *α* representing the weight of the initial modality fusion matrix ***Z***^(**0**)^, and the factor λ to balance the contribution of each modality. Similar to previous integration methods, *E* is very important on cell type classification. As a general suggestion, we recommend starting *E* at the minimal number of principle components (PCs) used in performing single modality clustering. Selection of λ allows us to adjust the size of modality-specific effects to reflect the divergence of the datasets being analyzed, and selection of couple coefficient *α* depends on whether the initial ***Z***^(**0**)^ can represent the “true” gene score of ***Y***. To aid the selection of λ, *α*, we devise two metrics to measure integration performance on accuracy (no mixing of cell type) and alignment (mixing of datasets) as defined below. Their applications to the data are shown in Additional file 2: Supplementary Notes 3-5 and Additional file 1: Fig. S14. These metrics do not rely on cell type labels or cell-cell correspondence and thus can be applied to new unlabeled data.

#### Silhouette score

To measure integration accuracy, we use the Silhouette score. Cluster for each cell is defined using the cell type labels assigned from single dataset clustering. The Silhouette score assesses the separation of cell types, where a high score suggests that cells of the same cell type are close together and far from cells of a different type. The Silhouette score *s*(*i*) for each cell is calculated as following. Let *a*(*i*) be the average distance of cell *i* to all other cells within *i*’s cluster and *b*(*i*) the average distance of *i* to all cells in the nearest cluster, to which cell *i* does not belong. Cell-cell distance is computed in the L2-normalized co-embeddings (Eq. 10). *s*(*i*) can be computed as follows:

15 $${\displaystyle \begin{array}{c}\ s(i)=\left\{\begin{array}{c}1-\frac{a(i)}{b(i)}\ if\ a(i)<b(i)\\ {}0\ if\ a(i)=b(i)\\ {}\frac{b(i)}{a(i)}-1\ if\ a(i)>b(i)\end{array}\right..\end{array}}$$

We average values across all cells to obtain an overall silhouette score for integration task.

#### Alignment mixing score

To measure integration mixing level, we use an alignment mixing score similar to those of previous studies [57]. We first build a 20-nearest neighbor graph for each cell from L2-normalized co-embeddings (Eq. 10). For cell *i*, assuming proportions of cells from two modalities are *p*_1*i*_ and *p*_2*i*_, respectively, the alignment mixing score is calculated as

16 $${\displaystyle \begin{array}{c}\ H(i)=-{p}_{1i}{\log}_2{p}_{1i}-{p}_{2i}{\log}_2{p}_{2i}\end{array}}$$

This corresponds to a mixing metric per cell, and we average values across all cells to obtain an overall mixing metric.

We run bindSC by ranging *a* from 0 to 1 (with step size 0.1) and λ from 0 to 1 (with step size 0.1). Silhouette score and alignment mixing score are calculated for each scenario. We select appropriate parameters that generally has best performance in Silhouette score and alignment mixing score. On any dataset, the optimal *a* can be determined using the *BiCCA_para_opt* functions in bindSC. Parameter values used in this study can be seen in Additional file 3: Table S1.

### Performance and benchmarking

In our evaluation, in addition to Silhouette score and alignment mixing score, we also consider anchoring distance for evaluation datasets from multi-omics technologies, in which each cell has paired profiles. For cell *i* from the first data, we calculate its distance (Euclidean distance) with all cells in the second data as ***D***_*i*_, and its distance with cell *i* in the second data as *d*_*i*_. The anchoring distance for cell *i* is calculated as 2*d*_*i*_/ *max* (***D***_*i*_). We then average anchoring distance across all cells to obtain an overall anchor distance metric. The anchoring distance of cell *i* is 0 when it is anchored correctly.

### Preparation of the mouse retina 10x Genomics Multiome ATAC+RNA data

One mouse retina was dissociated by papain-based enzymatic digestion as described previously [58] with slight modifications. Briefly, 45 U of activated papain solution (with 1.2 mg L-cysteine (Sigma) and 1200U of DNase I (Affymetrix) in 5ml of HBSS buffer) was added to the tissue and incubated at 37 °C for 20 min to release live cells. Post-incubation, papain solution was replaced and deactivated with ovomucoid solution (15 mg ovomucoid (Worthington biochemical) and 15 mg BSA (Thermo Fisher Scientific) in 10 ml of MEM (Thermo Fisher Scientific)). The remaining tissue clumps were further triturated in the ovomucoid solution and filtered through a 20-nm nylon mesh. After centrifugation at 300g 10min at 4C, the singe cells were resuspended PBS with 0.04% BSA and checked for viability and cell count. About 1 million cells were pelleted and resuspend in chilled lysis buffer (10x Genomics), incubate for 2 min on ice while monitored under microscope. One milliliter of chilled wash buffer (10x Genomics) was added, and the sample was spun down at 500g 5min at 4C and washed before resuspended in diluted nuclei buffer (10x Genomics). Nuclei concentration was determined using countess and proceed with transposition according to manufacturer’s recommendation (10x Genomics). After incubation for 1h at 37C, the transposed nuclei were combined with barcoded gel beads, RT mix, and partition oil on chromium to generate gel beads in emulsion (GEMs). Single-cell ATACseq library and 3’RNAseq library were subsequently generated following recommended protocol from 10x Genomics. Libraries were quantified and loaded on Novaseq 6000 and run with the following parameter: 151, 8, 8, and 151bp. Data was analyzed using bcl2fastq (to generate fastq files) and CellRanger pipeline (10x Genomics). Among 9383 detected high-quality nuclei, 1276 are gated as BCs by known markers for further analysis.

### Preparation of human bone marrow cell dataset

We examined the performance of bindSC in integrating the single-cell RNA and protein data derived from human bone marrow tissue. This dataset was generated using the CITE-seq technology [45], which included 30,672 cells that have joint profiles of RNA and a panel of 25 antibodies. We extracted the RNA expression of the coding genes for the 25 proteins profile from the RNA data and kept cells that have total expression count > 2.

The final protein matrix includes 28,609 cells with 25 protein abundance levels. The gene expression matrix includes 28,609 cells with 3000 genes. The protein-homologous RNA matrix includes 28,609 cells with the RNA levels of the 25 genes encoding the 25 proteins. To measure anchoring accuracy for each cell type, we used the third metric, anchoring distance, which measures the distance of protein and gene expression for each cell in co-embeddings.

### Preparation of CAR-NK dataset

The retroviral vectors encoding iC9.CAR19.CD28-zeta-2A-IL-15 and firefly luciferase (FFLuc) were generated as previously described [59]. Transient retroviral supernatant was produced, collected, and used for transduction of NK cells. CD56+ NK cells were isolated from cord blood units which were provided by MDACC Cord Blood Bank. Cord blood-derived NK cells were stimulated and transduced as previously described [60].

We used the paired scRNA-seq sequencing and mass cytometry (CyTOF) to characterize the NK cells that are (1) transduced with CD19CAR, (2) transduced with interleukin-15 (IL15), and (3) wildtype non-transduced (NT). Briefly, scRNA-seq data was pre-processed using the default pipeline Cell Ranger recommended by 10x Genomics. Mass cytometry data was saved in FCS files by a CyTOF instrument (Helios). We also excluded cells that were CD3+ to focus on NK cells only. Data from 3 groups were merged together using the R package cytofkit [61] on a set of 33 surface protein markers. Transformation using arcsinh with a cofactor of 5 were performed to facilitate comparison between samples. For each surface marker, the maximum intensity observed over the 99.5th percentile across all samples was excluded to avoid high-intensity outliers. Data from all samples were divided by these maximum values. As a result, intensity values for each marker ranged from 0 to 1. Finally, we obtained scRNA-seq data matrix having (1341 cells × 33,538 genes) and CyTOF data matrix (59,510 cells × 29 proteins) from the three groups. For bindSC integration, we downsampled 2000 cells from CyTOF data to avoid the integration bias driven by imbalanced cell numbers.

### Analysis of the fetal muscle dataset

The fetal muscle sci-RNA-seq dataset was downloaded from https://descartes.brotmanbaty.org/bbi/human-gene-expression-during-development/, and the fetal muscle sci-ATAC-seq dataset was downloaded from https://descartes.brotmanbaty.org/bbi/human-chromatin-during-development/.

We obtained sci-RNA-seq data (47,537 cells by 63,561 genes) and sci-ATAC-seq data (27,181 cells by 1,084,870 peaks). For quality control, we further removed cells with less than 100 genes expressed and genes that exist in less than 500 cells from the sci-RNA-seq data. We also removed cells with less than 1000 peaks expressed and peaks that exist in less than 500 cells from the sci-ATAC-seq data. The final RNA matrix includes 30,872 cells by 5000 highly variable genes and the ATAC matrix includes 22,552 cells by 43,889 peaks.

To validate cell type assignment for cells from sci-ATAC-seq data, we performed gene set enrichment analysis on differentially expressed genes and differentially chromatin accessible genes using Enrichr [62] (https://maayanlab.cloud/Enrichr/). We obtained GO biological processes pathways and Janseen Cell Type Topology with at least 4 genes and adjusted *p* value < 0.1.

### Calculating the correlation between imputed molecular profiles and the ground-truth

The modality fusion matrix ***Z*** in bindSC can be considered as the imputed profiles of cells from ***Y*** on the first modality. Given mouse retina bipolar dataset and human bone marrow dataset are from co-assayed profiles, the Pearson correlation between updated ***Z*** and ***X*** (they share the same dimension) can reflect the accuracy of bindSC integration. The overall Pearson correlation was calculated by treating ***X*** and ***Z*** as vectors. The cell-type level Pearson correlation was calculated by using entries of ***X*** and ***Z*** from a specific cell type.

### Motif-based TF activity estimation

To estimate transcription factor activity from scATAC-seq data, we used default settings in chromVAR [63] package. This approach quantifies accessibility variation across single cells by aggregating accessible regions containing a specific TF motif. It calculated motif-based TF activity by comparing the observed accessibility of all the peaks containing a TF motif to a background set of peaks normalizing against known technical confounders.

### Building and visualizing protein-gene networks

For human bone marrow dataset measured with CITE-seq technology, we calculated the Pearson correlation of each pair of protein and gene based on updated ***Z*** (from bindSC) and ***Y***. A cutoff of 0.55 is used to filter the relations. For visualization purpose, we further keep no more than five genes for each individual protein.

## Supplementary Information

**Additional file 1: Fig. S1.** Implementation of bindSC for large datasets. **Fig. S2.** Benchmarking bindSC performance on simulation datasets. **Fig. S3.** Classification of BC subtypes. **Fig. S4.** Comparison of bindSC with other tools on the mouse retina BC cells data. **Fig. S5.** Peak-gene links inferred from bipolar cells clustered by subtypes. **Fig. S6.** Gene-peak visualization of BC cell type marker genes. **Fig. S7.** Motif-based Transcription factors (TFs) analysis of bipolar cells (BCs) based on bindSC integration. **Fig. S8.** Comparison of bindSC with other tools on the bone marrow data. **Fig. S9.** Downstream analysis of CITE-seq data based on bindSC’s integration. **Fig. S10.** Gene expression level of JUN, FOS, and TNF for integrated clusters from bindSC. **Fig. S11.** Protein marker expression level for integrated clusters from bindSC. **Fig. S12.** Downstream analysis of human fetal atlas using bindSC. **Fig. S13.** Improvement of gene activity matrix Z after bindSC alignment. **Fig. S14.** Effect of bindSC parameter *α* and *λ* based on integration metrics. **Fig. S15.** Change of objective function cost over the iteration time. **Fig. S16.** Comparison of integration using initial modality fusion matrix calculated by different models on retina data.

**Additional file 2: Supplementary Note 1.** Previous studies on multi-omics integration. **Supplementary Note 2.** Simulation Study. **Supplementary Note 3.** Effect of parameters on integration results. **Supplementary Note 4.** The iteration process of bindSC. **Supplementary Note 5.** Effect of initial fusion matrix on integration results.

**Additional file 3: Table S1.** Parameter settings used in benchmarking experiments.

**Additional file 4.** Review history.

## Data Availability

BindSC is implemented as an open-source R package available at https://github.com/KChen-lab/bindSC [64], and the latest release is hosted by Zenodo [65] under the GNU General Public License v3.0. The human bone marrow dataset was generated using the CITE-seq technology, which was downloaded from Seurat website https://satijalab.org/seurat/v4.0/weighted_nearest_neighbor_analysis.html. The fetal muscle sci-RNA-seq dataset was downloaded from Descartes database https://descartes.brotmanbaty.org/bbi/human-gene-expression-during-development/ [66], and the fetal muscle sci-ATAC-seq dataset was downloaded from https://descartes.brotmanbaty.org/bbi/human-chromatin-during-development/ [5]. The mouse retina 10x Genomics ATAC+RNA data is available in Gene Expression Omnibus (GEO) with accession number GSE201402 [67]. The scRNA-seq data in CAR-NK dataset is available in GEO with accession number GSE190976 [68], and the protein data is available in Flow Repository with ID FR-FCM-Z59C [69].

## References

[1] Macosko EZ, Basu A, Satija R, Nemesh J, Shekhar K, Goldman M, Tirosh I, Bialas AR, Kamitaki N, Martersteck EM (2015). Highly parallel genome-wide expression profiling of individual cells using nanoliter droplets. Cell.

[2] Spitzer MH, Nolan GP (2016). Mass cytometry: single cells, many features. Cell.

[3] Ma A, McDermaid A, Xu J, Chang Y, Ma Q (2020). Integrative methods and practical challenges for single-cell multi-omics. Trends Biotechnol.

[4] Teichmann S, Efremova M (2020). Method of the year 2019: single-cell multimodal omics. Nat Methods.

[5] Domcke S, Hill AJ, Daza RM, Cao J, O’Day DR, Pliner HA, Aldinger KA, Pokholok D, Zhang F, Milbank JH (2020). A human cell atlas of fetal chromatin accessibility. Science.

[6] Luo C, Keown CL, Kurihara L, Zhou J, He Y, Li J, Castanon R, Lucero J, Nery JR, Sandoval JP (2017). Single-cell methylomes identify neuronal subtypes and regulatory elements in mammalian cortex. Science.

[7] Mulqueen RM, Pokholok D, Norberg SJ, Torkenczy KA, Fields AJ, Sun D, Sinnamon JR, Shendure J, Trapnell C, O'Roak BJ (2018). Highly scalable generation of DNA methylation profiles in single cells. Nat Biotechnol.

[8] Cao J, Cusanovich DA, Ramani V, Aghamirzaie D, Pliner HA, Hill AJ, Daza RM, McFaline-Figueroa JL, Packer JS, Christiansen L (2018). Joint profiling of chromatin accessibility and gene expression in thousands of single cells. Science.

[9] Cusanovich DA, Hill AJ, Aghamirzaie D, Daza RM, Pliner HA, Berletch JB, Filippova GN, Huang X, Christiansen L, DeWitt WS (2018). A single-cell atlas of in vivo mammalian chromatin accessibility. Cell.

[10] Lake BB, Chen S, Sos BC, Fan J, Kaeser GE, Yung YC, Duong TE, Gao D, Chun J, Kharchenko PV (2018). Integrative single-cell analysis of transcriptional and epigenetic states in the human adult brain. Nat Biotechnol.

[11] Moffitt JR, Bambah-Mukku D, Eichhorn SW, Vaughn E, Shekhar K, Perez JD, Rubinstein ND, Hao J, Regev A, Dulac C (2018). Molecular, spatial, and functional single-cell profiling of the hypothalamic preoptic region. Science.

[12] Wang X, Allen WE, Wright MA, Sylwestrak EL, Samusik N, Vesuna S, Evans K, Liu C, Ramakrishnan C, Liu J (2018). Three-dimensional intact-tissue sequencing of single-cell transcriptional states. Science.

[13] Dey SS, Kester L, Spanjaard B, Bienko M, Van Oudenaarden A (2015). Integrated genome and transcriptome sequencing of the same cell. Nat Biotechnol.

[14] Macaulay IC, Haerty W, Kumar P, Li YI, Hu TX, Teng MJ, Goolam M, Saurat N, Coupland P, Shirley LM (2015). G&T-seq: parallel sequencing of single-cell genomes and transcriptomes. Nat Methods.

[15] Argelaguet R, Clark SJ, Mohammed H, Stapel LC, Krueger C, Kapourani C-A, Imaz-Rosshandler I, Lohoff T, Xiang Y, Hanna CW (2019). Multi-omics profiling of mouse gastrulation at single-cell resolution. Nature.

[16] Ma S, Zhang B, LaFave LM, Earl AS, Chiang Z, Hu Y, Ding J, Brack A, Kartha VK, Tay T (2020). Chromatin potential identified by shared single-cell profiling of RNA and chromatin. Cell.

[17] Zhu C, Preissl S, Ren B (2020). Single-cell multimodal omics: the power of many. Nat Methods.

[18] Tran HTN, Ang KS, Chevrier M, Zhang X, Lee NYS, Goh M, Chen J (2020). A benchmark of batch-effect correction methods for single-cell RNA sequencing data. Genome Biol.

[19] Wang C, Sun D, Huang X, Wan C, Li Z, Han Y, Qin Q, Fan J, Qiu X, Xie Y (2020). Integrative analyses of single-cell transcriptome and regulome using MAESTRO. Genome Biol.

[20] Stuart T, Butler A, Hoffman P, Hafemeister C, Papalexi E, Mauck WM, Hao Y, Stoeckius M, Smibert P, Satija R (2019). Comprehensive integration of single-cell data. Cell.

[21] Korsunsky I, Millard N, Fan J, Slowikowski K, Zhang F, Wei K, et al. Fast, sensitive and accurate integration of single-cell data with Harmony. Nat Methods. 2019;16(12):1289–96. 10.1038/s41592-019-0619-0 PMC6884693 31740819

[22] Rosenberg AB, Roco CM, Muscat RA, Kuchina A, Sample P, Yao Z, Graybuck LT, Peeler DJ, Mukherjee S, Chen W (2018). Single-cell profiling of the developing mouse brain and spinal cord with split-pool barcoding. Science.

[23] Singh R, Demetci P, Bonora G, Ramani V, Lee C, Fang H, et al. Unsupervised manifold alignment for single-cell multi-omics data. In Proceedings of the 11th ACM International Conference on Bioinformatics, Computational Biology and Health Informatics. 2020. p. 1–10. 10.1145/3388440.3412410 PMC8095090 33954299

[24] Cao K, Bai X, Hong Y, Wan L. Unsupervised topological alignment for single-cell multi-omics integration. Bioinformatics. 2020;36(Supplement_1):i48–i56. 10.1093/bioinformatics/btaa443 PMC7355262 32657382

[25] Welch JD, Hartemink AJ, Prins JF (2017). MATCHER: manifold alignment reveals correspondence between single cell transcriptome and epigenome dynamics. Genome Biol.

[26] Pliner HA, Packer JS, McFaline-Figueroa JL, Cusanovich DA, Daza RM, Aghamirzaie D, Srivatsan S, Qiu X, Jackson D, Minkina A (2018). Cicero predicts cis-regulatory DNA interactions from single-cell chromatin accessibility data. Mol Cell.

[27] Lieberman-Aiden E, Van Berkum NL, Williams L, Imakaev M, Ragoczy T, Telling A, Amit I, Lajoie BR, Sabo PJ, Dorschner MO (2009). Comprehensive mapping of long-range interactions reveals folding principles of the human genome. Science.

[28] Duren Z, Chen X, Zamanighomi M, Zeng W, Satpathy AT, Chang HY, Wang Y, Wong WH (2018). Integrative analysis of single-cell genomics data by coupled nonnegative matrix factorizations. Proc Natl Acad Sci.

[29] Lara-Astiaso D, Weiner A, Lorenzo-Vivas E, Zaretsky I, Jaitin DA, David E, Keren-Shaul H, Mildner A, Winter D, Jung S (2014). Chromatin state dynamics during blood formation. Science.

[30] Liu J, Huang Y, Singh R, Vert J-P, Noble WS. Jointly embedding multiple single-cell omics measurements. BioRxiv. 2019:644310. 10.4230/LIPIcs.WABI.2019.10 PMC8496402 34632462

[31] Cao K, Hong Y, Wan L. Manifold alignment for heterogeneous single-cell multi-omics data integration using Pamona. Bioinformatics. 2022;38(1):211–9. 10.1093/bioinformatics/btab594 PMC8696097 34398192

[32] Demetci P, Santorella R, Sandstede B, Noble WS, Singh R. Gromov-Wasserstein optimal transport to align single-cell multi-omics data. BioRxiv. 2020.

[33] Zappia L, Phipson B, Oshlack A (2017). Splatter: simulation of single-cell RNA sequencing data. Genome Biol.

[34] Liang Q, Dharmat R, Owen L, Shakoor A, Li Y, Kim S, Vitale A, Kim I, Morgan D, Liang S (2019). Single-nuclei RNA-seq on human retinal tissue provides improved transcriptome profiling. Nat Commun.

[35] Masland RH (2012). The neuronal organization of the retina. Neuron.

[36] Menon M, Mohammadi S, Davila-Velderrain J, Goods BA, Cadwell TD, Xing Y, Stemmer-Rachamimov A, Shalek AK, Love JC, Kellis M (2019). Single-cell transcriptomic atlas of the human retina identifies cell types associated with age-related macular degeneration. Nat Commun.

[37] Clark BS, Stein-O’Brien GL, Shiau F, Cannon GH, Davis-Marcisak E, Sherman T, Santiago CP, Hoang TV, Rajaii F, James-Esposito RE (2019). Single-cell RNA-seq analysis of retinal development identifies NFI factors as regulating mitotic exit and late-born cell specification. Neuron.

[38] Shekhar K, Lapan SW, Whitney IE, Tran NM, Macosko EZ, Kowalczyk M, Adiconis X, Levin JZ, Nemesh J, Goldman M (2016). Comprehensive classification of retinal bipolar neurons by single-cell transcriptomics. Cell.

[39] Brunet I, Weinl C, Piper M, Trembleau A, Volovitch M, Harris W, Prochiantz A, Holt C (2005). The transcription factor Engrailed-2 guides retinal axons. Nature.

[40] Nishida A, Furukawa A, Koike C, Tano Y, Aizawa S, Matsuo I, Furukawa T (2003). Otx2 homeobox gene controls retinal photoreceptor cell fate and pineal gland development. Nat Neurosci.

[41] Marquardt T, Ashery-Padan R, Andrejewski N, Scardigli R, Guillemot F, Gruss P (2001). Pax6 is required for the multipotent state of retinal progenitor cells. Cell.

[42] Ramanathan M, Porter DF, Khavari PA (2019). Methods to study RNA–protein interactions. Nat Methods.

[43] Krishnaswamy S, Spitzer MH, Mingueneau M, Bendall SC, Litvin O, Stone E, Pe’er D, Nolan GP (2014). Conditional density-based analysis of T cell signaling in single-cell data. Science.

[44] Efremova M, Teichmann S (2020). Computational methods for single-cell omics across modalities. Nat Methods.

[45] Stoeckius M, Hafemeister C, Stephenson W, Houck-Loomis B, Chattopadhyay PK, Swerdlow H, Satija R, Smibert P (2017). Simultaneous epitope and transcriptome measurement in single cells. Nat Methods.

[46] Liu E, Marin D, Banerjee P, Macapinlac HA, Thompson P, Basar R, Nassif Kerbauy L, Overman B, Thall P, Kaplan M (2020). Use of CAR-transduced natural killer cells in CD19-positive lymphoid tumors. N Engl J Med.

[47] Robertson MJ (2002). Role of chemokines in the biology of natural killer cells. J Leukoc Biol.

[48] Garni-Wagner BA, Purohit A, Mathew PA, Bennett M, Kumar V (1993). A novel function-associated molecule related to non-MHC-restricted cytotoxicity mediated by activated natural killer cells and T cells. J Immunol.

[49] Granja JM, Corces MR, Pierce SE, Bagdatli ST, Choudhry H, Chang H, et al. ArchR is a scalable software package for integrative single-cell chromatin accessibility analysis. Nat Genet. 2021;53(3):403–11. 10.1038/s41588-021-00790-6 PMC8012210 33633365

[50] Rozenblatt-Rosen O, Stubbington MJ, Regev A, Teichmann SA (2017). The Human Cell Atlas: from vision to reality. Nat News.

[51] Consortium H (2019). The human body at cellular resolution: the NIH Human Biomolecular Atlas Program. Nature.

[52] Rozenblatt-Rosen O, Regev A, Oberdoerffer P, Nawy T, Hupalowska A, Rood JE, Ashenberg O, Cerami E, Coffey RJ, Demir E (2020). The Human Tumor Atlas Network: charting tumor transitions across space and time at single-cell resolution. Cell.

[53] Sharma A, Seow JJW, Dutertre C-A, Pai R, Blériot C, Mishra A, Wong RMM, Singh GSN, Sudhagar S, Khalilnezhad S (2020). Onco-fetal reprogramming of endothelial cells drives immunosuppressive macrophages in hepatocellular carcinoma. Cell.

[54] Warren A, Jones A, Shibue T, Hahn WC, Boehm JS, Vazquez F, et al. Global computational alignment of tumor and cell line transcriptional profiles. Nature Commun. 2021;12(1):1–12. 10.1038/s41467-020-20294-x PMC7782593 33397959

[55] Hardoon DR, Szedmak S, Shawe-Taylor J (2004). Canonical correlation analysis: an overview with application to learning methods. Neural Comput.

[56] Traag VA, Waltman L, van Eck NJ (2019). From Louvain to Leiden: guaranteeing well-connected communities. Sci Rep.

[57] Welch JD, Kozareva V, Ferreira A, Vanderburg C, Martin C, Macosko EZ (2019). Single-cell multi-omic integration compares and contrasts features of brain cell identity. Cell.

[58] Siegert S, Cabuy E, Scherf BG, Kohler H, Panda S, Le Y-Z, Fehling HJ, Gaidatzis D, Stadler MB, Roska B (2012). Transcriptional code and disease map for adult retinal cell types. Nat Neurosci.

[59] Hoyos V, Savoldo B, Quintarelli C, Mahendravada A, Zhang M, Vera J, Heslop HE, Rooney CM, Brenner MK, Dotti G (2010). Engineering CD19-specific T lymphocytes with interleukin-15 and a suicide gene to enhance their anti-lymphoma/leukemia effects and safety. Leukemia.

[60] Liu E, Tong Y, Dotti G, Shaim H, Savoldo B, Mukherjee M, Orange J, Wan X, Lu X, Reynolds A (2018). Cord blood NK cells engineered to express IL-15 and a CD19-targeted CAR show long-term persistence and potent antitumor activity. Leukemia.

[61] Chen H, Lau MC, Wong MT, Newell EW, Poidinger M, Chen J (2016). Cytofkit: a bioconductor package for an integrated mass cytometry data analysis pipeline. PLoS Comput Biol.

[62] Kuleshov MV, Jones MR, Rouillard AD, Fernandez NF, Duan Q, Wang Z, Koplev S, Jenkins SL, Jagodnik KM, Lachmann A (2016). Enrichr: a comprehensive gene set enrichment analysis web server 2016 update. Nucleic Acids Res.

[63] Schep AN, Wu B, Buenrostro JD, Greenleaf WJ (2017). chromVAR: inferring transcription-factor-associated accessibility from single-cell epigenomic data. Nat Methods.

[64] Dou J, Liang S, Chen K. biCCA: bi-order multimodal integration of single-cell data: Github; 2022. https://github.com/KChen-lab/bindSC.git 10.1186/s13059-022-02679-x PMC9082907 35534898

[65] Dou J, Liang S, Chen K. biCCA: bi-order multimodal integration of single-cell data: Zenodo; 2022. 10.5281/zenodo.6448220. 10.1186/s13059-022-02679-x PMC9082907 35534898

[66] Cao J, O’Day DR, Pliner HA, Kingsley PD, Deng M, Daza RM, Zager MA, Aldinger KA, Blecher-Gonen R, Zhang F (2020). A human cell atlas of fetal gene expression. Science.

[67] Dou J, Liang S, Chen K, Chen R. biCCA: bi-order multimodal integration of single cell data: Gene Expression Omnibus; 2022. https://www.ncbi.nlm.nih.gov/geo/query/acc.cgi?acc=GSE201402 10.1186/s13059-022-02679-x PMC9082907 35534898

[68] Li L, Vakul M, Dou J, Huang Y, Chen K, Rezvani K (2022). Gene expression omnibus.

[69] Dou J, Liang S, Rezvani K, Chen K. biCCA: bi-order multimodal integration of single-cell data: FLOW Repository; 2022. http://flowrepository.org/id/FR-FCM-Z59C 10.1186/s13059-022-02679-x PMC9082907 35534898

