# Supplementary material for: Bi-order multimodal integration of single-cell data
Source: Genome Biol. 2022 May 9;23:112. doi: 10.1186/s13059-022-02679-x (PMC9082907; doi:10.1186/s13059-022-02679-x)
Supplement: Supplementary file 1 — Additional file 1: Fig. S1. Implementation of bindSC for large datasets. Fig. S2. Benchmarking bindSC performance on simulation datasets. Fig. S3. Classification of BC subtypes. Fig. S4. Comparison of bindSC with other tools on the mouse retina BC cells data. Fig. S5. Peak-gene links inferred from bipolar cells clustered by subtypes. Fig. S6. Gene-peak visualization of BC cell type marker genes. Fig. S7. Motif-based Transcription factors (TFs) analysis of bipolar cells (BCs) based on bindSC integration. Fig. S8. Comparison of bindSC with other tools on the bone marrow data. Fig. S9. Downstream analysis of CITE-seq data based on bindSC’s integration. Fig. S10. Gene expression level of JUN, FOS, and TNF for integrated clusters from bindSC. Fig. S11. Protein marker expression level for integrated clusters from bindSC. Fig. S12. Downstream analysis of human fetal atlas using bindSC. Fig. S13. Improvement of gene activity matrix Z after bindSC alignment. Fig. S14. Effect of bindSC parameter α and λ based on integration metrics. Fig. S15. Change of objective function cost over the iteration time. Fig. S16. Comparison of integration using initial modality fusion matrix calculated by different models on retina data. [file 13059_2022_2679_MOESM1_ESM.pdf]

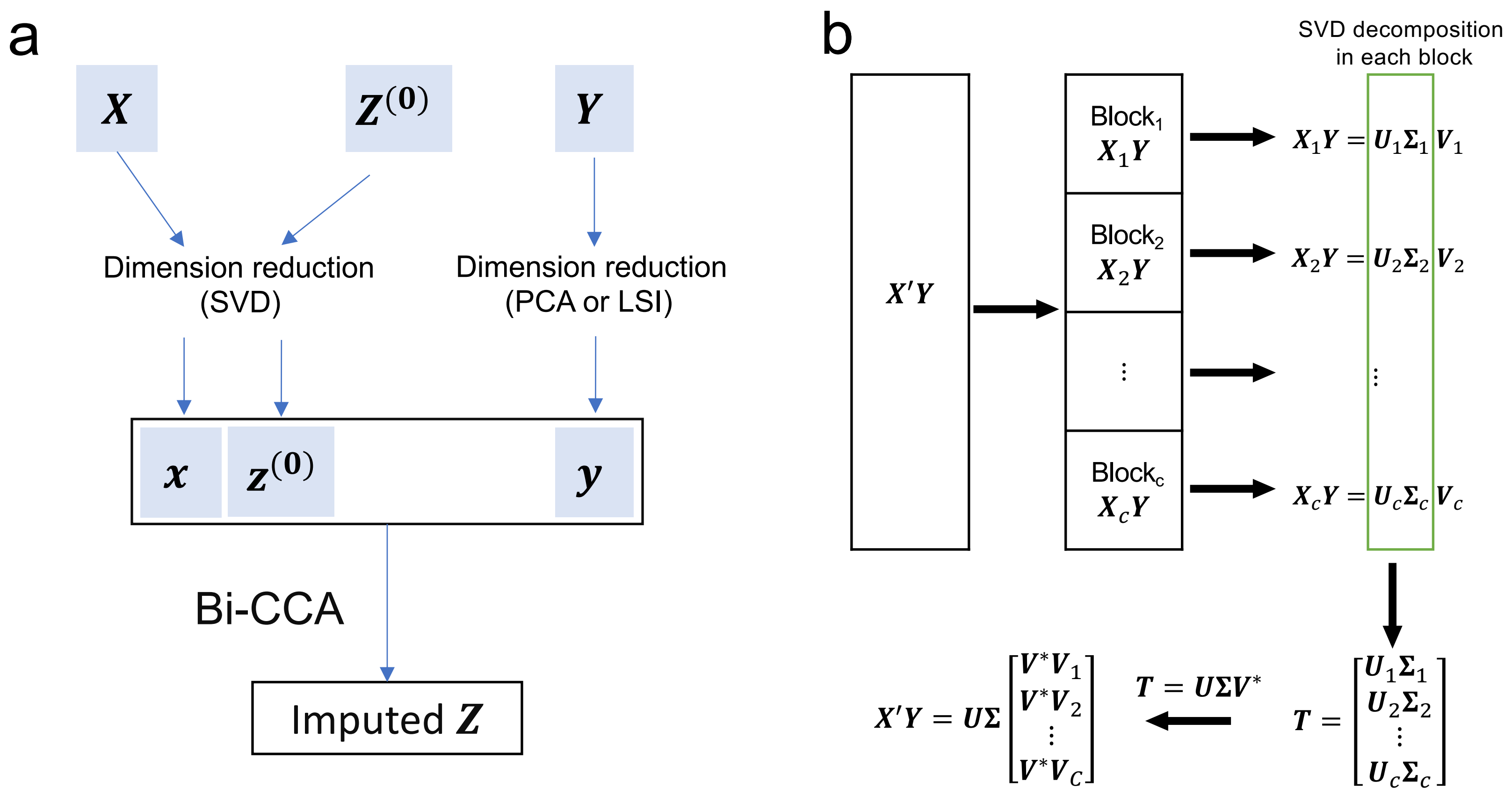

Fig. S1: Implementation of bindSC for large datasets.

(a) The major computational cost of bindSC is from the SVD decomposition on high-dimensional dataset. To address this issue, bindSC can utilize low-dimension representations rather than original full datasets which will reduce the computational cost when the feature dimension is high. The singular value decomposition (SVD) is performed on paired matrices (**X**, **Z**$^{(0)}$). The principal component analysis (PCA) or latent sematic indexing (LSI) is performed on **Y**. Bi-CCA can take the low dimension representations of **X**, **Z**$^{(0)}$, and **Y** as input and modality infusion matrix ***Z*** can be obtained from Equation (**9**).

(b) Implementation of divide-and-conquer SVD in bi-CCA for extra-large matrices. It splits cell into different blocks, performs SVD in each block, and merges the results from each chunk into the final decomposition.

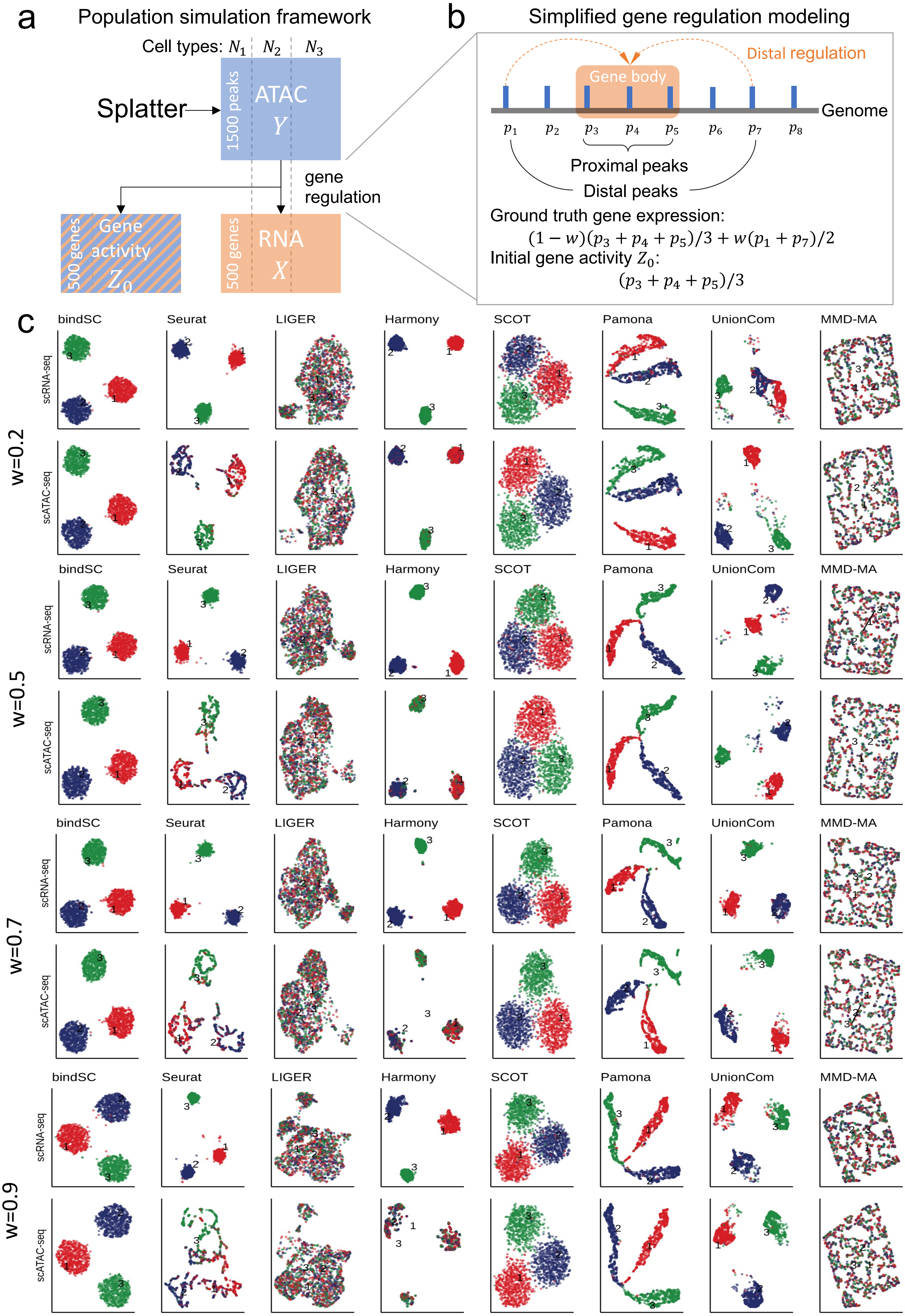
a
Population simulation framework
Cell types: N_1 N_2 N_3
Splatter
1500 peaks
ATAC
Y
gene
regulation
500 genes
Gene
activity
Z_0
500 genes
RNA
X
b
Simplified gene regulation modeling
Distal regulation
Gene body
Genome
p_1 p_2 p_3 p_4 p_5 p_6 p_7 p_8
Proximal peaks
Distal peaks
Ground truth gene expression:
$(1-w)(p_3+p_4+p_5)/3+w(p_1+p_7)/2$
Initial gene activity Z_0:
$(p_3+p_4+p_5)/3$
c
bindSC
Seurat
LIGER
Harmony
SCOT
Pamona
UnionCom
MMD-MA
scRNA-seq
scATAC-seq
w=0.2
w=0.5
w=0.7
w=0.9

(cont'd)

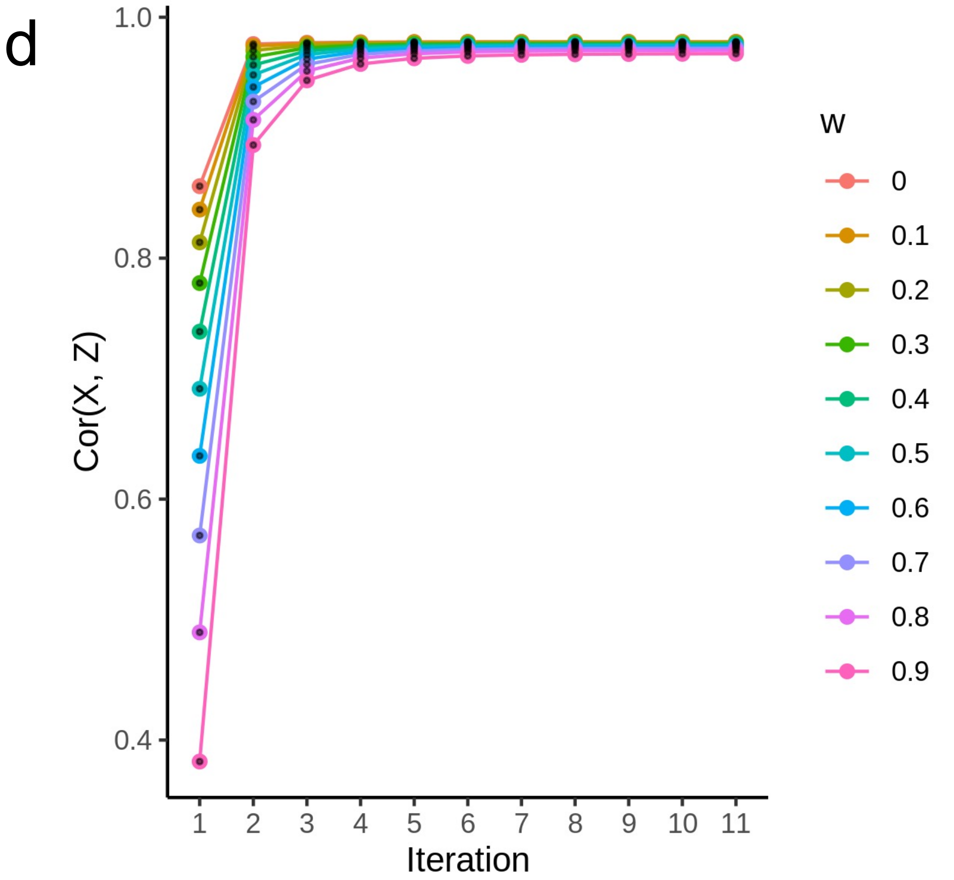

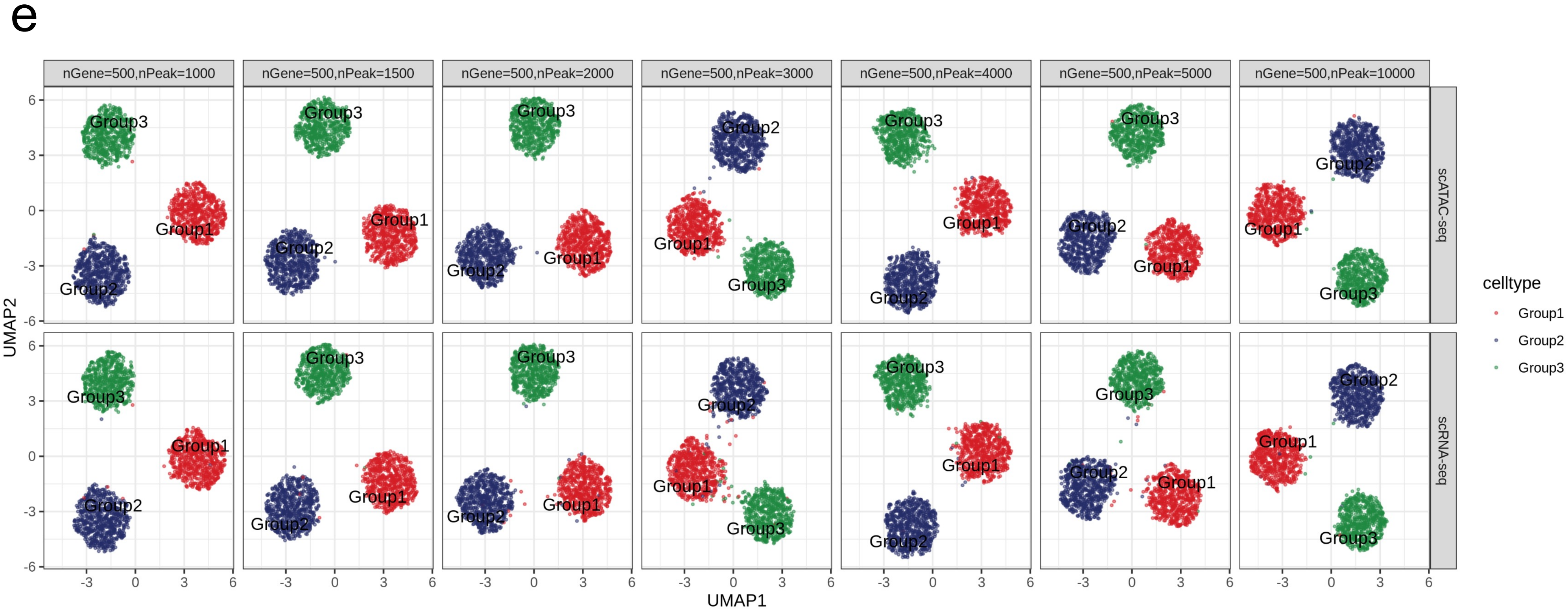

Fig. S2: Benchmarking bindSC performance on simulation datasets.

(a) Simulation framework using Splatter, based on 3 types of cells with 500 genes, 1500 peaks. Both RNA levels and gene activities are calculated from the ATAC peak levels as detailed in (b).

(b) Simulation of gene regulation. The expression of a gene is regulated by proximal peaks p_3, p_4 and p_5 in gene body and distal peaks p_1 and p_8 out of the gene body. The weight for distal peaks is w. The gene activity score is the unweighted mean of the proximal peaks.

(c) UMAP co-embeddings generated by all the methods for $w = 0.2$, 0.5, 0.7, and 0.9, plotted separated for scRNA and scATAC data (color: cell types)

(d) Pearson correlation (y-axis) of the updated gene score matrix Z and the gold standard X at each bindSC iteration (x-axis). Results are shown for the first 11 iterations only. Each line denotes the results from a specified w. The correlation was calculated by treating X and Z as vectors.

(e) scATAC-seq (top) and scRNA-seq (bottom) co-embedding results of bindSC on simulated data with 500 genes and 1,000 to 10,000 peaks.

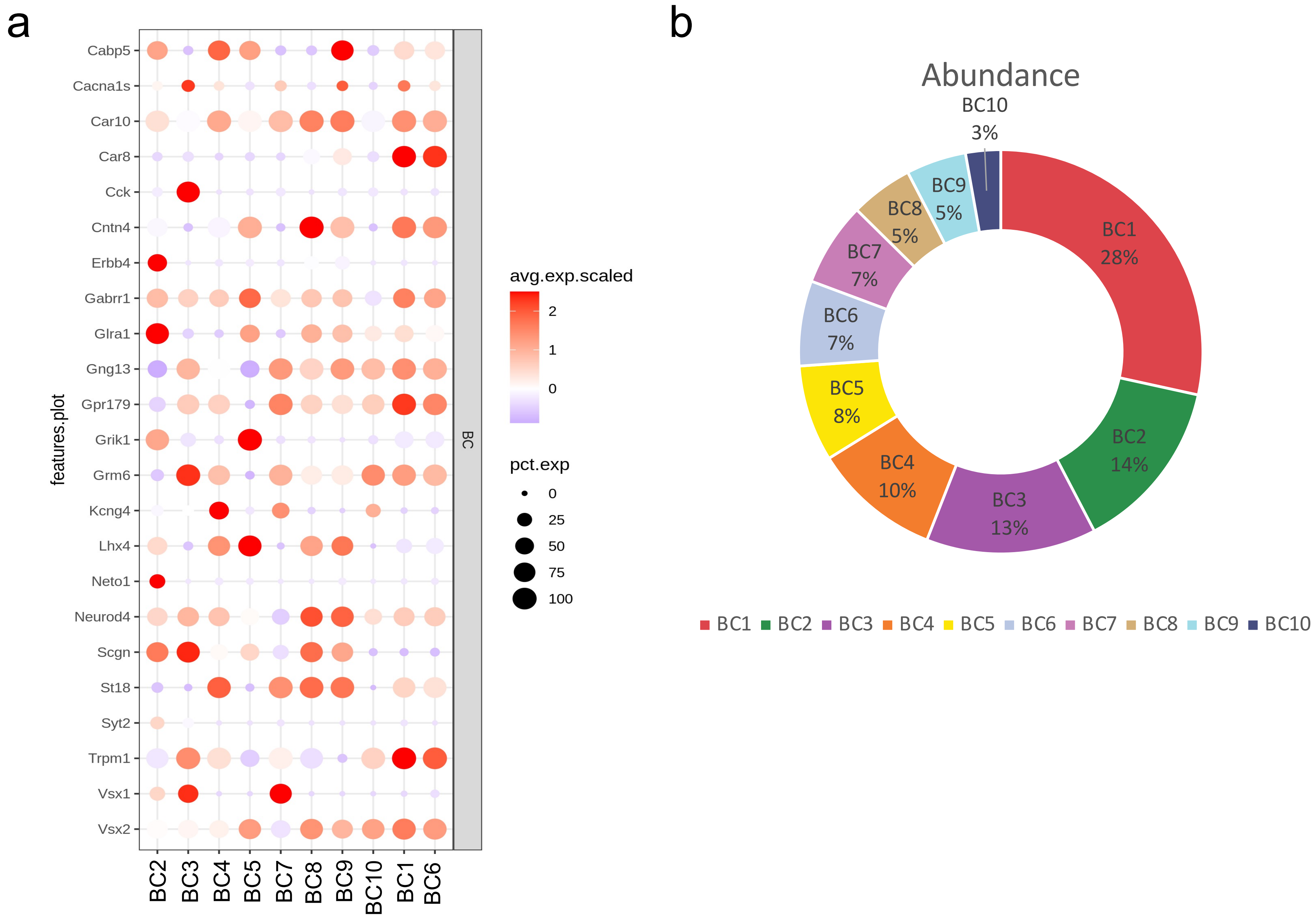

Fig. S3. Classification of BC subtypes

(a) Expression level of known marker genes related to retina cell types on 10 BC subtypes.

(b) Relative abundance of BC subtypes.

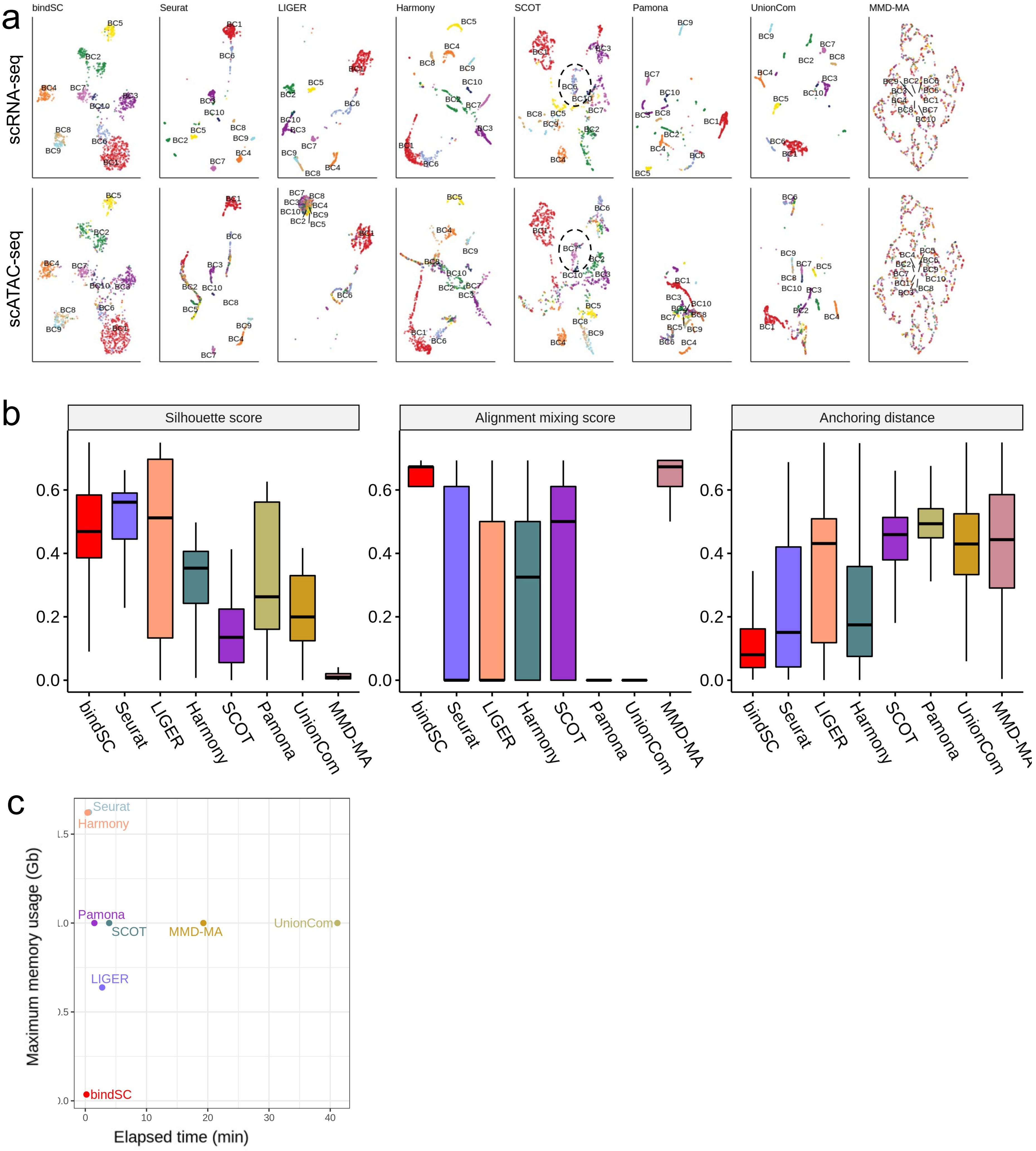

Fig. S4 Comparison of bindSC with other tools on the mouse retina BC cells data

(a) UMAPs with cells colored by cell types annotated based on snRNA-seq data. The top panel shows results for sn-RNA and bottom panel for sn-ATAC.

(**b**) Comparison based on silhouette score (left), alignment mixing score (middle) and anchoring distance (right).

(**c**) Comparison of memory and time consumption on a 28-core Intel Skylake CPU@2.6GHz. MMD-MA, Pamona, and SCOT are run with multi-threading settings.

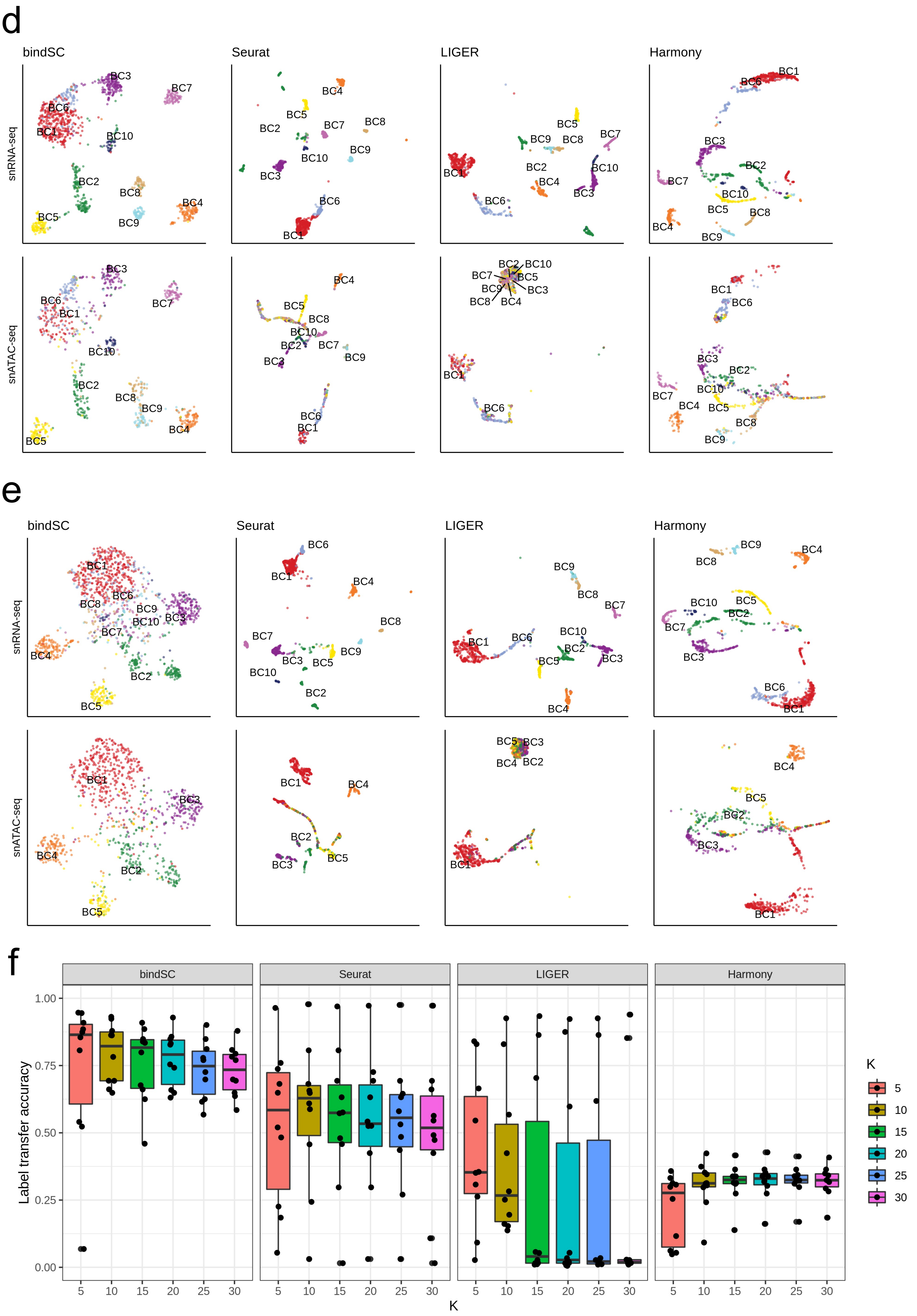

Fig. S4 Comparison of bindSC with other tools on the mouse retina BC cells data (continued).
(**d**) UMAP co-embeddings generated by four methods on imbalanced dataset of scenario 1. The top panel shows results for snRNA-seq and the bottom panel for snATAC-seq.
(**e**) UMAP co-embeddings generated by four methods on imbalanced dataset of scenario 2. The top panel shows results for snRNA-seq and the bottom panel for snATAC-seq.
(**f**) Label transfer accuracy for varying the latent space *k*. For mouse retina data, we range *k* from 5 to 30. In each boxplot, each point denotes one cell type. When running bindSC, we set α=0.5 and λ=0.5.

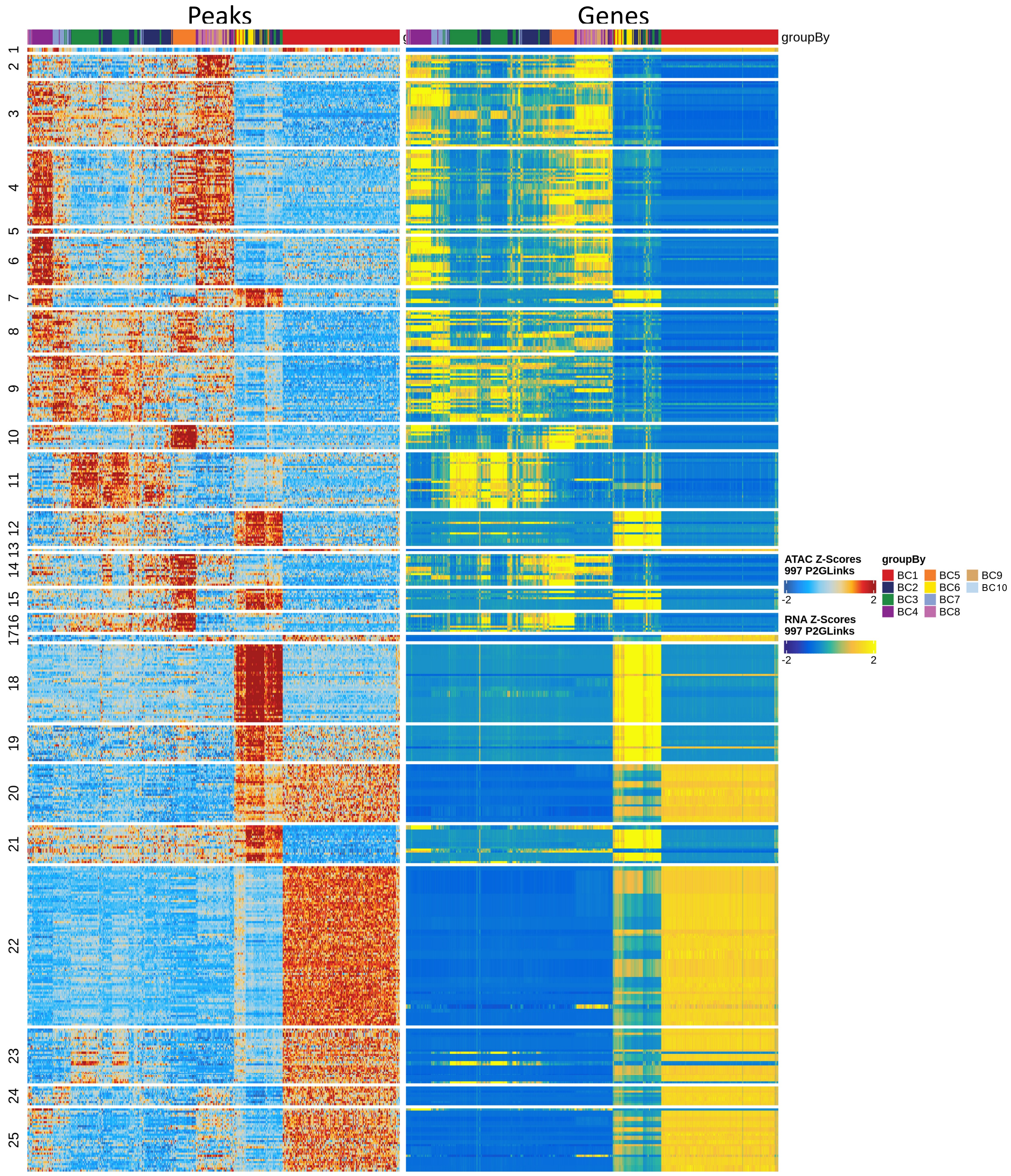

Fig. S5 Peak-gene links inferred from bipolar cells clustered by subtypes.

Each column denotes one cell, and each row denotes one gene-peak link. Left panel for peak profiles and right panel for gene expression profiles. The links are further clustered into 25 modules which show correspondence with one or more BC subtypes.

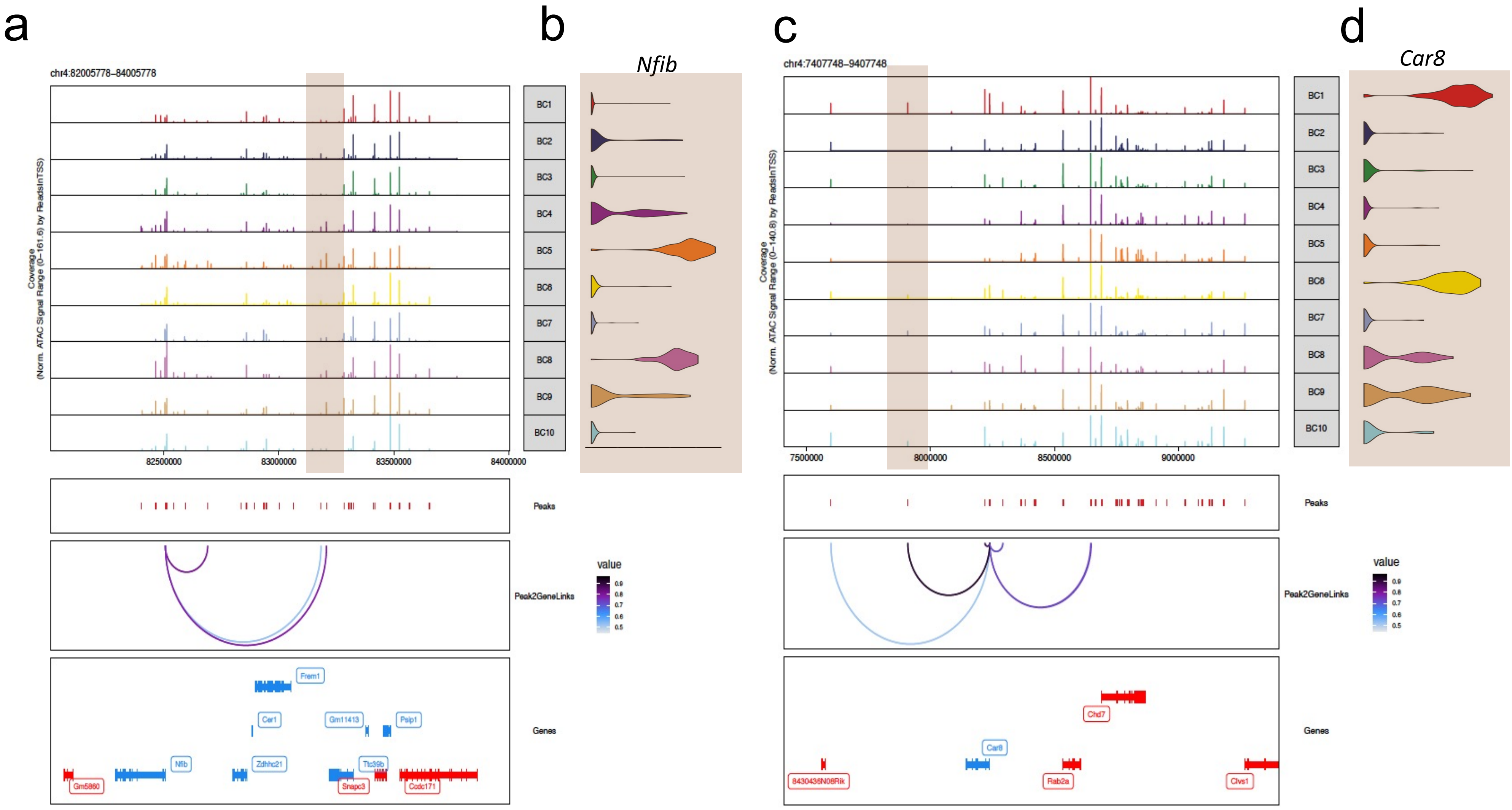

Fig. S6 Gene-peak visualization of BC cell type marker genes.
(**a-b**) Nfib genome track (a) (chr4:83,285,500-83,285,999) showing greater accessibility in BC4, BC5, BC8 and BC9, conforming with *Nfib* gene expression (b). Locations of peaks, genes, and links between them are shown in the bottom panels.
(**c-d**) (chr4:7,907,500-7,907,999) shows greater accessibility in BC1 (c), conforming with *Car8* gene expression (d).

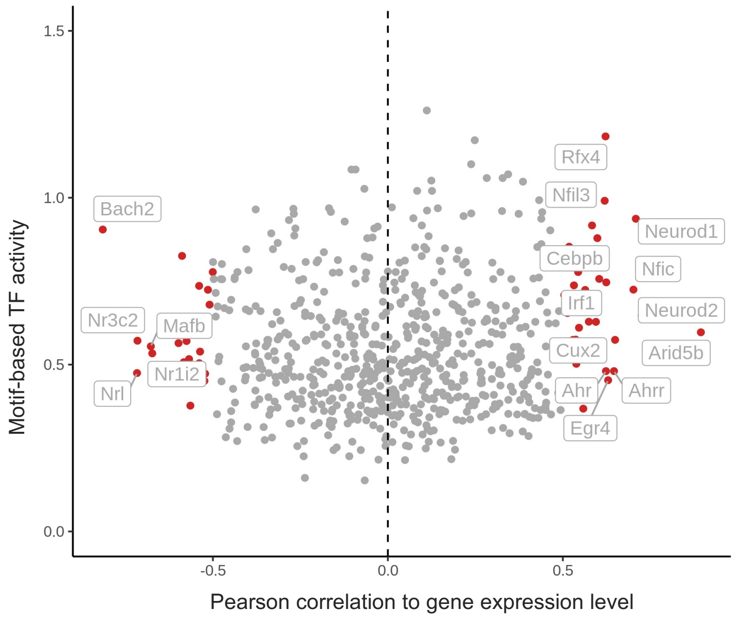

Fig. S7 Motif-based Transcription factors (TFs) analysis of bipolar cells (BCs) based on bindSC integration

Correlation of motif-based activity and gene expression level for transcription factors based on bindSC integration. The TFs with absolute correlation values higher than 0.5 are highlighted in red color. The TF activity is estimated from their motif activity in the ATAC profile using ChromVAR (**Methods**).

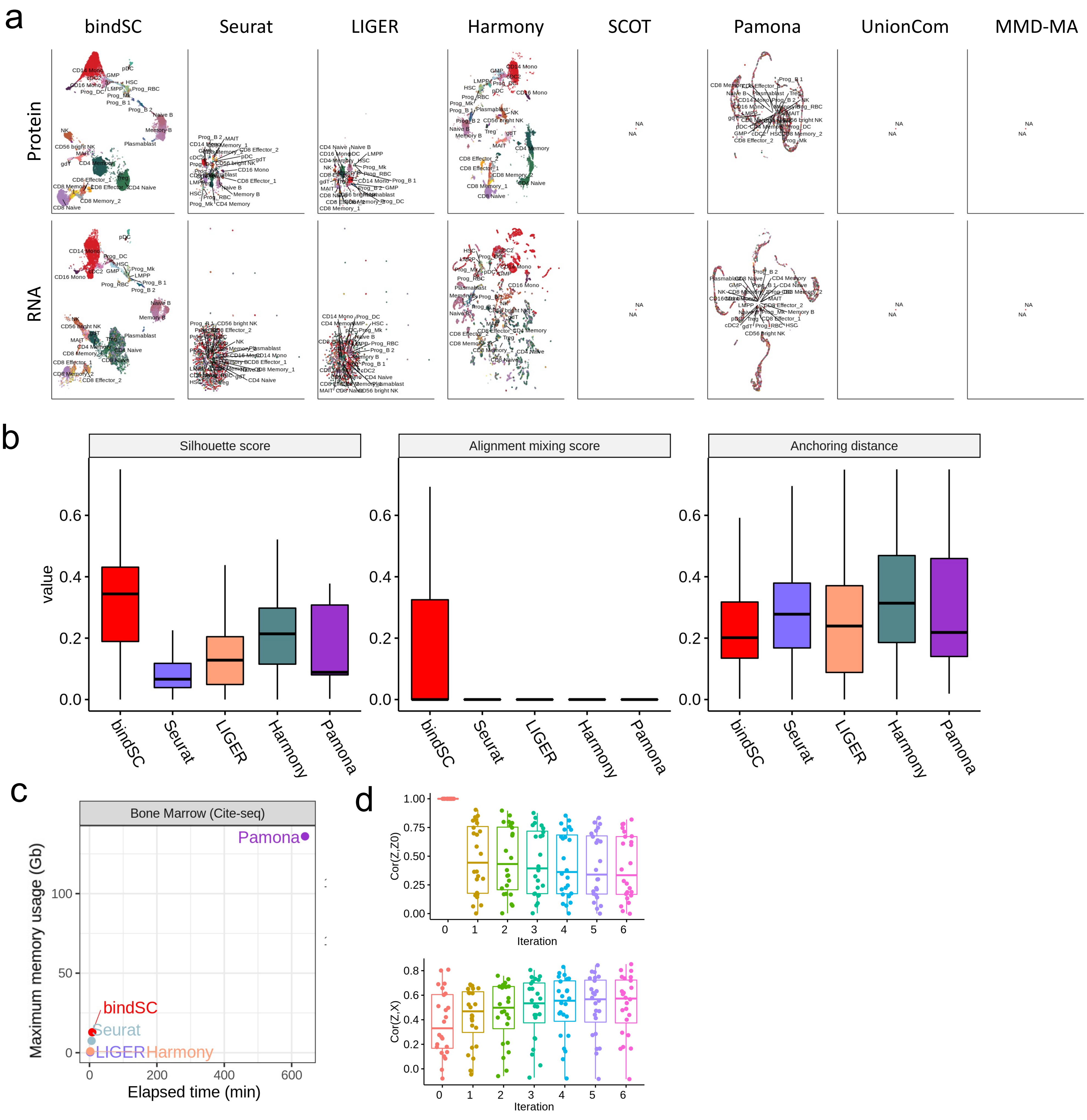

Fig. S8 Comparison of bindSC with other tools on the bone marrow data.

(a) UMAP of cells colored by cell types annotated based on scRNA-seq data. The top panel shows results for protein and bottom panel for RNA data.

(b) Comparison based on silhouette score (left), alignment mixing score (middle) and anchoring distance (right).

(c) Time and memory usage of compared methods on a 28-core Intel Skylake CPU@2.6GHz. MMD-MA, Pamona, and SCOT are run with multi-threading settings. SCOT and UnionCom were not able to complete in 24 hours. MMD-MA exceeded the memory limit of 360GB.

(d) Pearson correlation of protein levels imputed by integration and the initial gene scores (top panel) and the ground-true protein levels (bottom panel). The result at 0 iteration is the same as the traditional CCA method. Each dot represents a cell type.

e

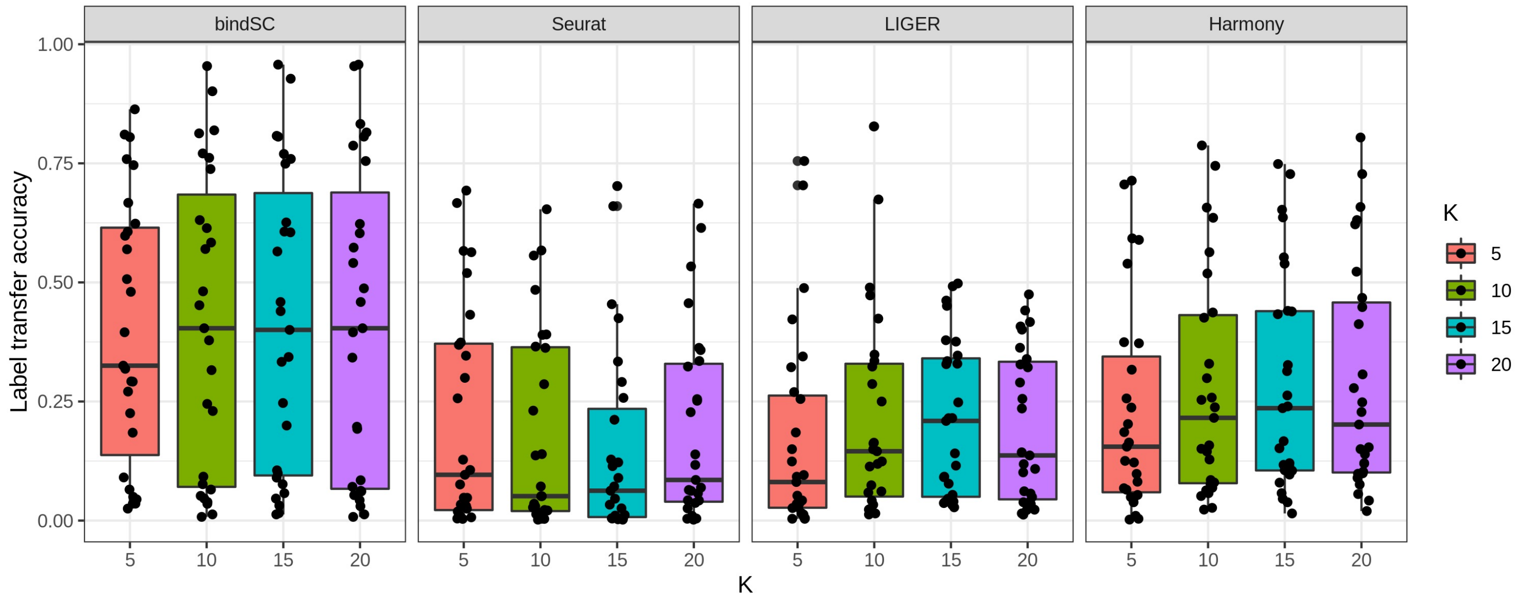

Fig. S8 Comparison of bindSC with other tools on the bone marrow data (continued).

(e) Label transfer accuracy for varying the latent space *k*. For human bone marrow data, there is only 25 protein markers available, thus we set *k* from 5 to 20. In each boxplot, each point denotes one cell type. When running bindSC, we set α=0.5 and λ=0.5.

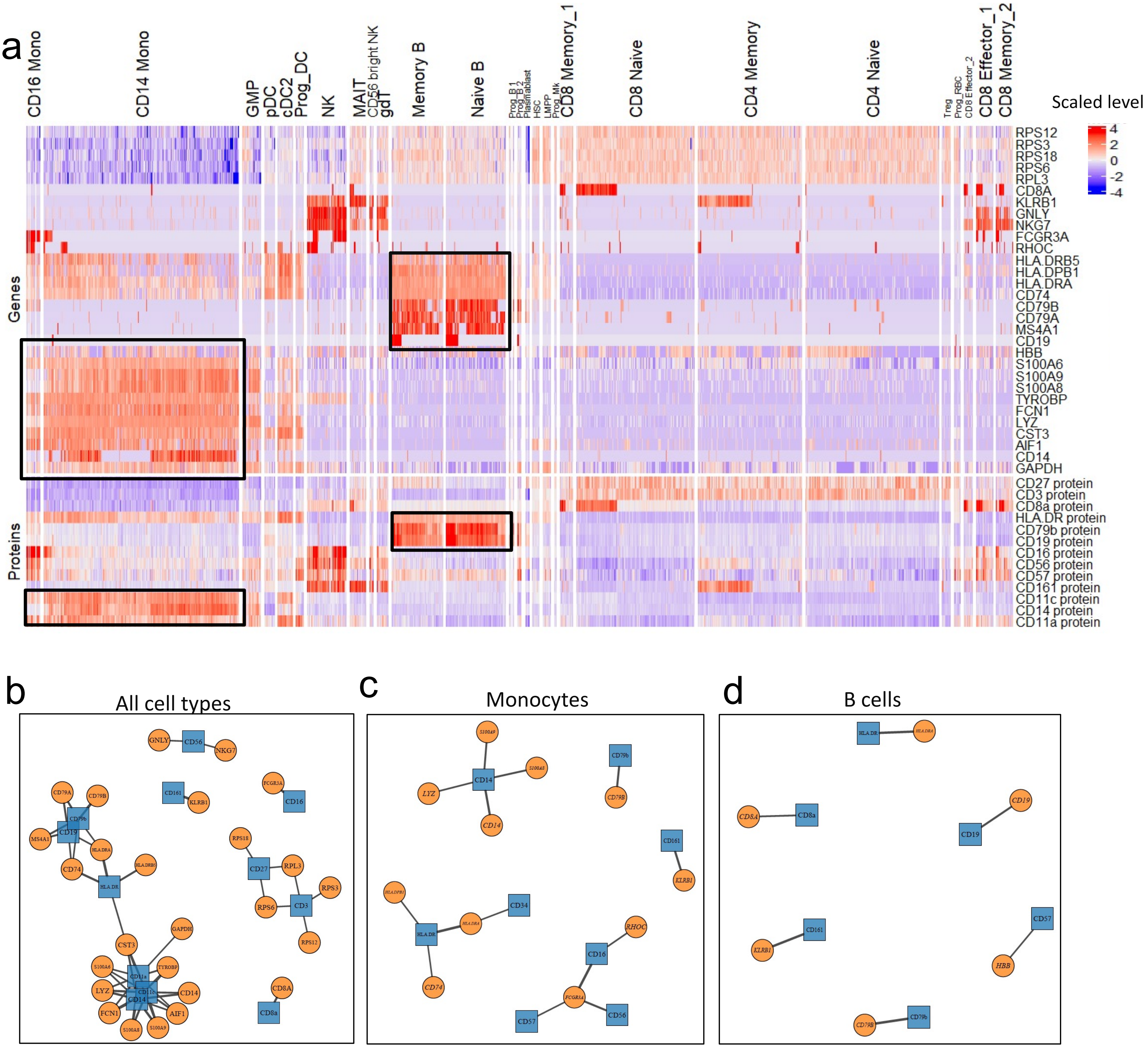

Fig. S9 Downstream analysis of CITE-seq data based on bindSC's integration

(**a**) Heatmap of relevant genes and proteins for the CITE-seq data. The monocyte/B cells related genes and proteins are highlighted in box. (**b**-**d**) Full gene-protein network inferred using all cell types (b), monocytes (c), and B cells (d). Width of edges are corresponding to the correlation. A threshold of 0.55 is used on Pearson correlation of genes and inferred protein levels. The network showing in **Fig. 3** is the largest connected component in (b).

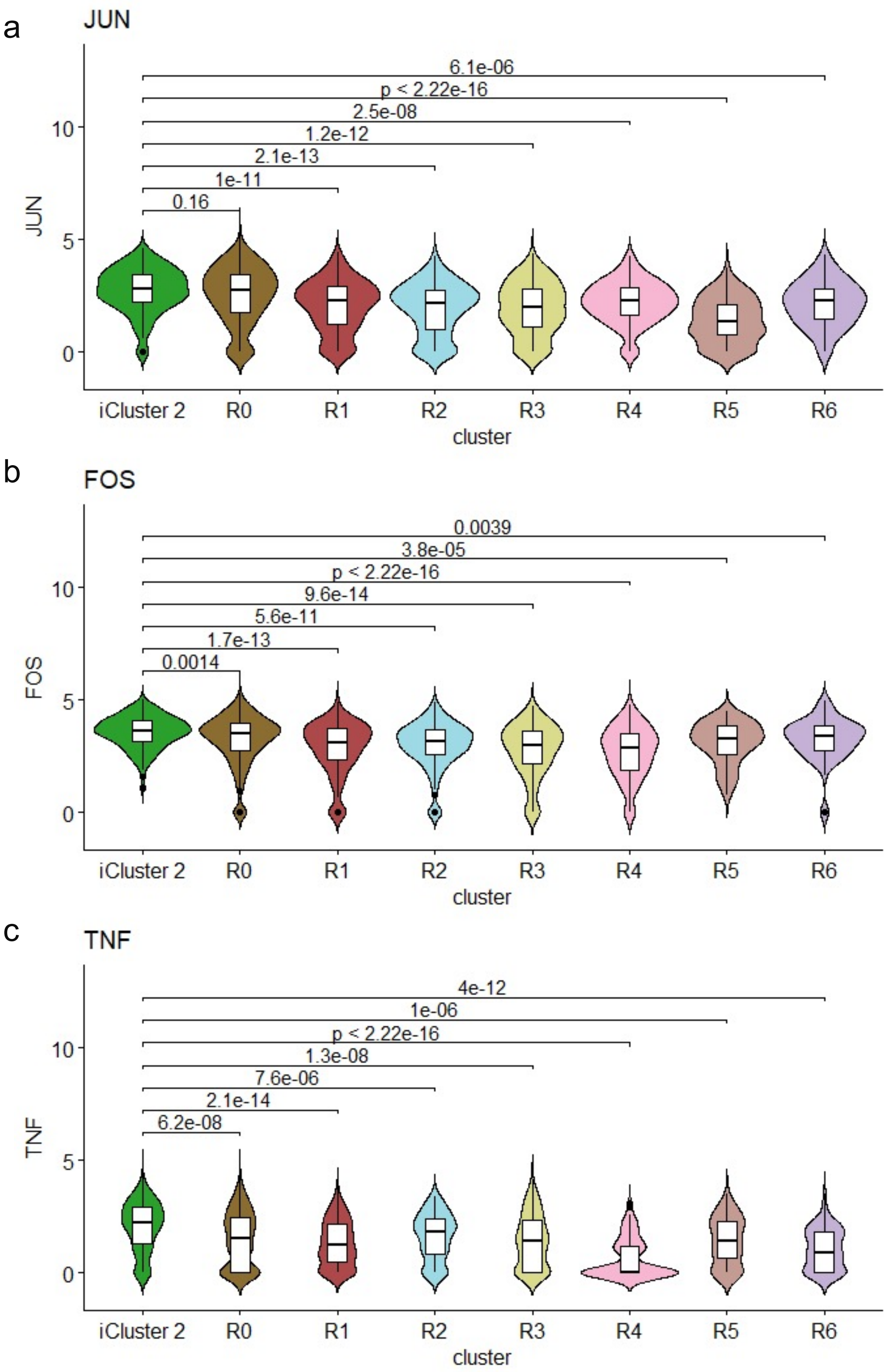

Fig. S10. Gene expression level of JUN, FOS, and TNF for integrated clusters from bindSC.
Gene expression levels of *JUN* (**a**), *FOS* (**b**), and *TNF* (**c**) in iCluster 2 compared with rClusters. P-values are from the Wilcoxon test.

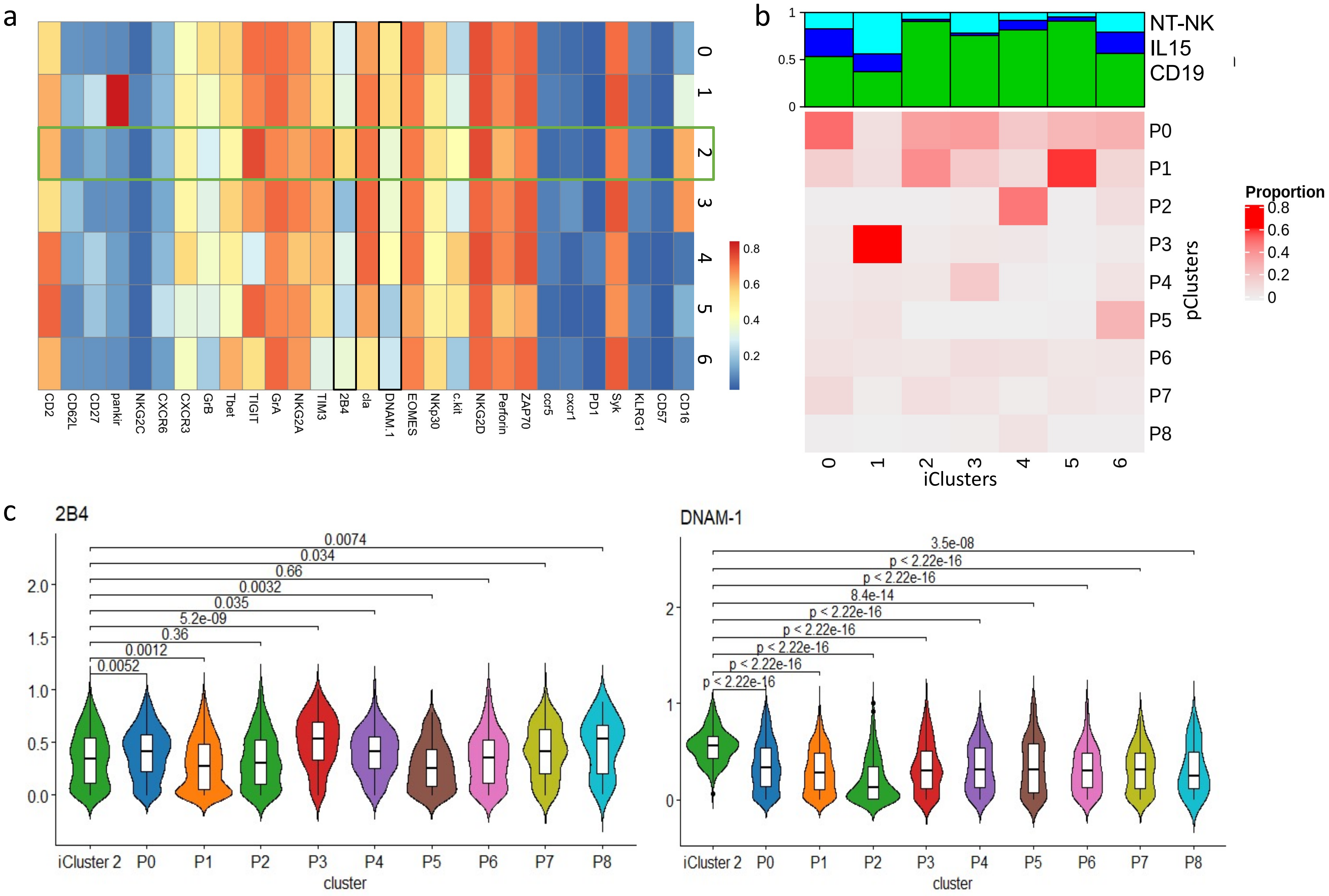

Fig. S11 Protein marker expression level for integrated clusters from bindSC.
(a) Protein marker expression in CyTOF data for iClusters. iCluster 2 is highlighted.
(b) Correspondence of iClusters, pClusters, and cell groups. The color denotes the proportion of iCluster in each protein cluster, normalized by each column. The top annotation shows frequencies of three cell groups (cyan: NT-NK, blue: IL15, green: CD19) in each iCluster.
(c-d) Protein expression levels of 2B4 and DNAM-1 expressions compare between iCluster 2 and pClusters. P-values are from the Wilcoxon test.

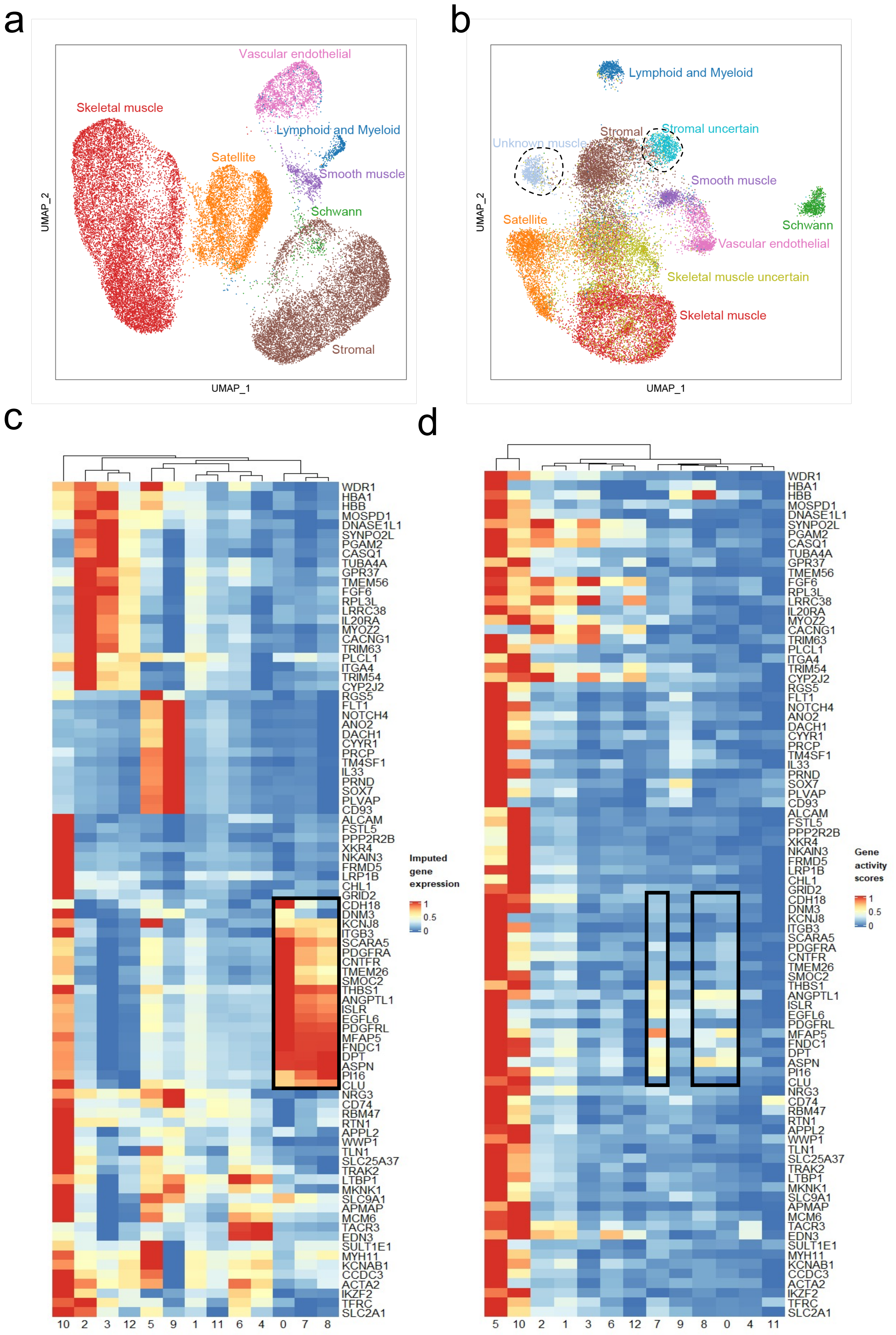

Fig. S12 Downstream analysis of human fetal atlas using bindSC.
(**a-b**) UMAPs of RNA (a) and ATAC (b) data obtained directly from the original publication. Clusters in question are circled out.
(**c-d**) Comparison of gene expression imputed by bindSC (a) and gene activity scores (b). Common activated genes of cluster 0, 7, and 8 are highlighted. The dendrograms on the top shows hierarchical clustering of cell clusters. BindSC improved the accuracy of imputed gene expression and lead to accurate identification of cluster 7 and 8.

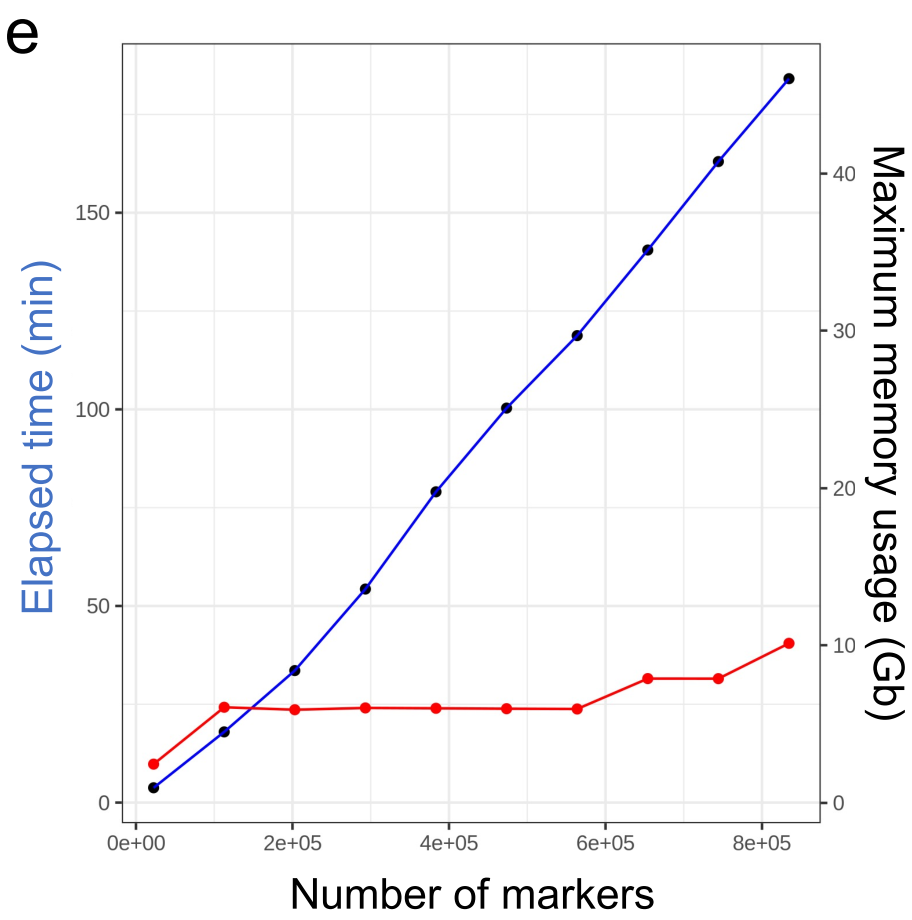

Fig. S12 (continued)

(**e**) The total running time (left) and maximum memory (right) required for bindSC on running each dataset is shown. We created ten benchmark datasets with cells number ranging from 22,552 to 834,424 by resampling cells from the fetal muscle atlas. The block size is set to 1,000 for bindSC in each dataset.

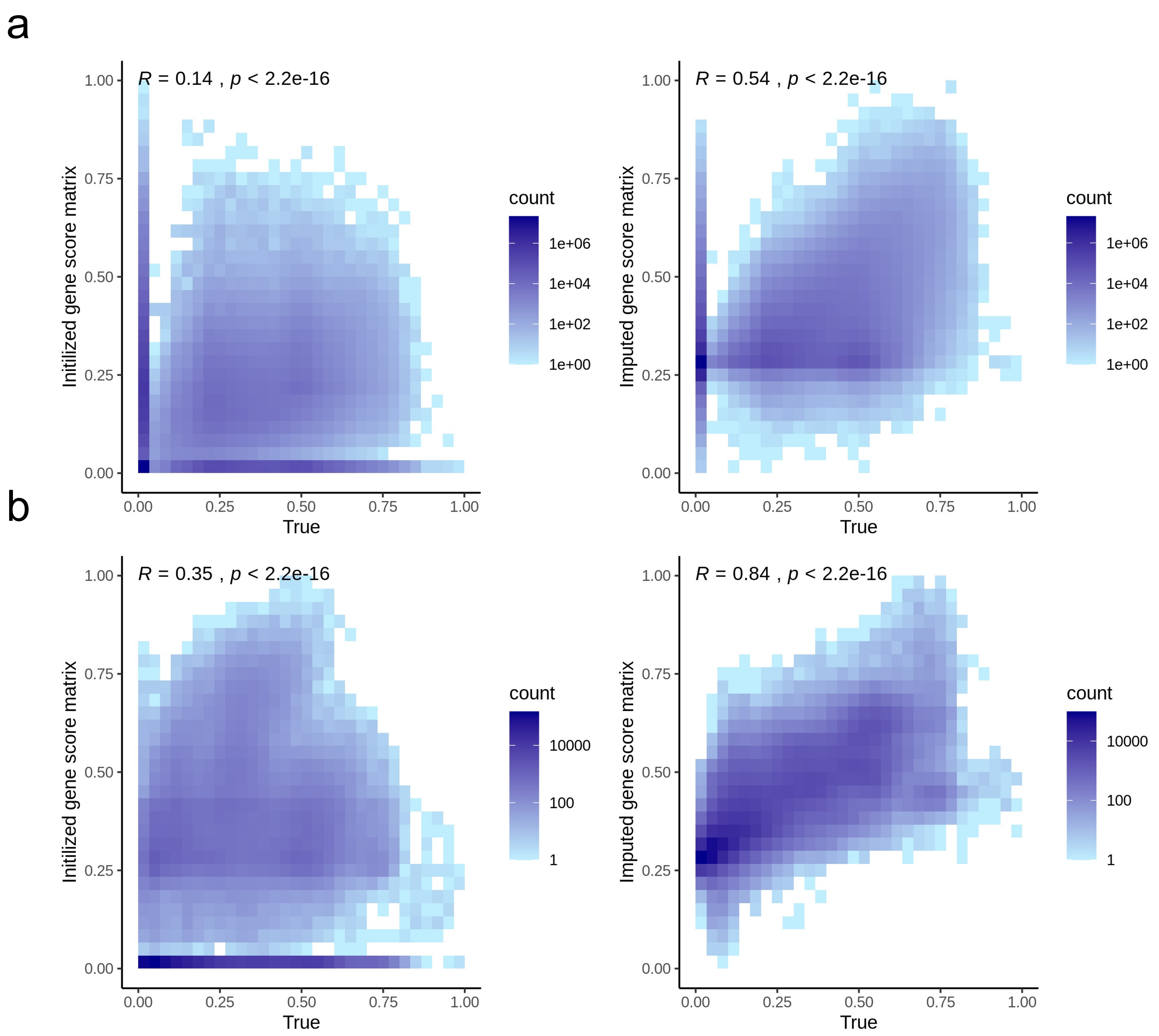

Fig. S13 Improvement of gene activity matrix Z after bindSC alignment. Results are shown for 2 benchmarking datasets that cell correspondence is known between two modalities. From top to bottom mouse retinal cell atlas (**a**), and bone marrow data (**b**). For each dataset, X-axes are the gene score matrix derived from co-assayed cell profile (which is served as the gold standard) and Y-axes are the initialized gene score (left panel) and imputed gene score (right panel). The overall concordance between X and Y is quantified using the R value.

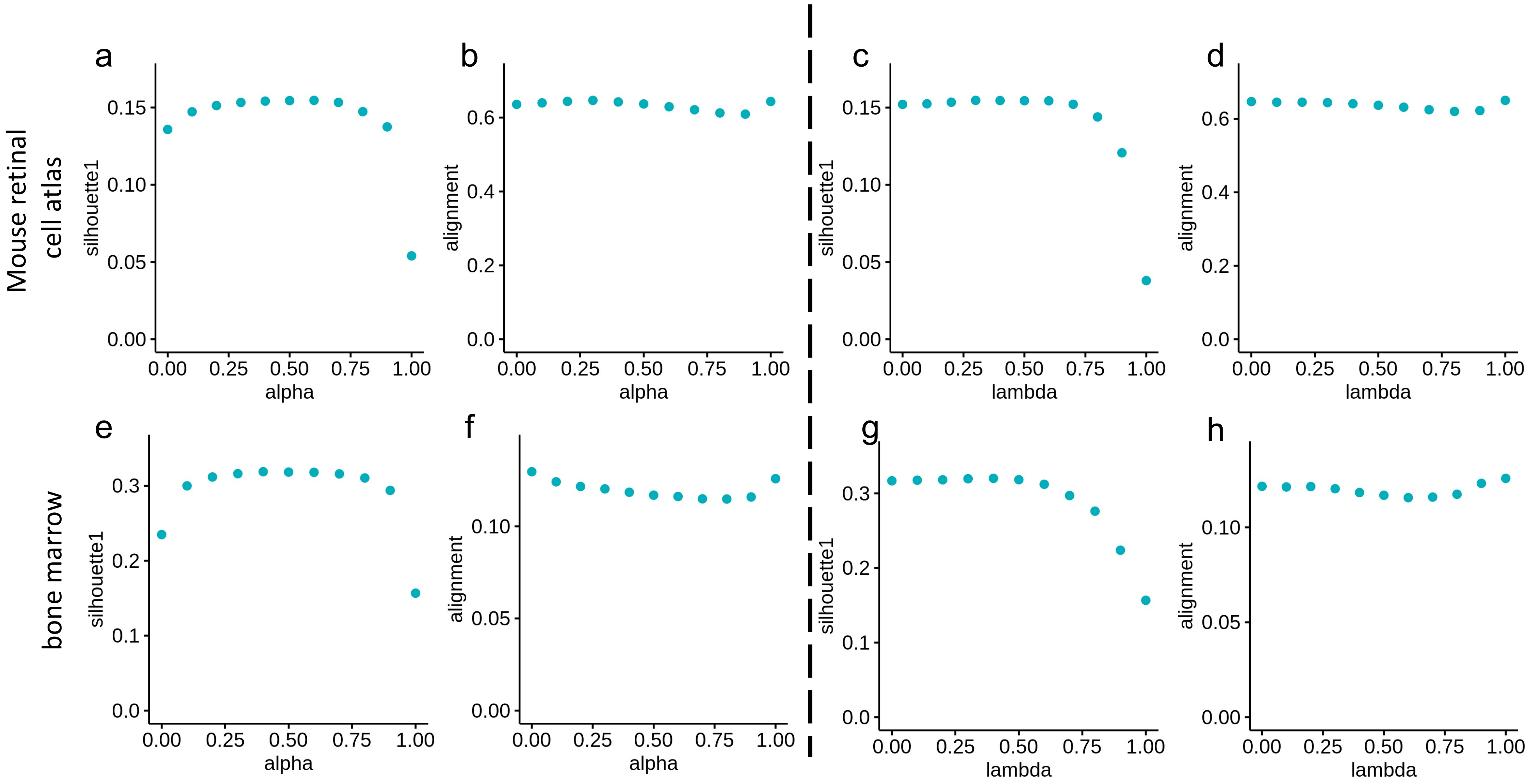

Fig. S14. Effect of bindSC parameter α and λ based on integration metrics. Results are shown for the two benchmarking datasets used. **a**,**b**,**e**,**f** shows the effect of α on integration metrices including the Silhouette score (left; measures the distortion of the RNA data) and the alignment mixing score (right; measures the degree of integration). Neither of them rely on labels and thus can be used for new unlabeled data to determine the parameters. λ was fix to 0.5, 0.5 based on those criteria, respectively, while α varies. **c**,**d**,**g**,**h** shows the effect of λ on the Silhouette score (left) and the alignment mixing score (right). Neither of them rely on labels and thus can be used for new unlabeled data to determine the parameters. α was fix to 0.5, 0.5 based on those criteria, respectively, while λ varies.

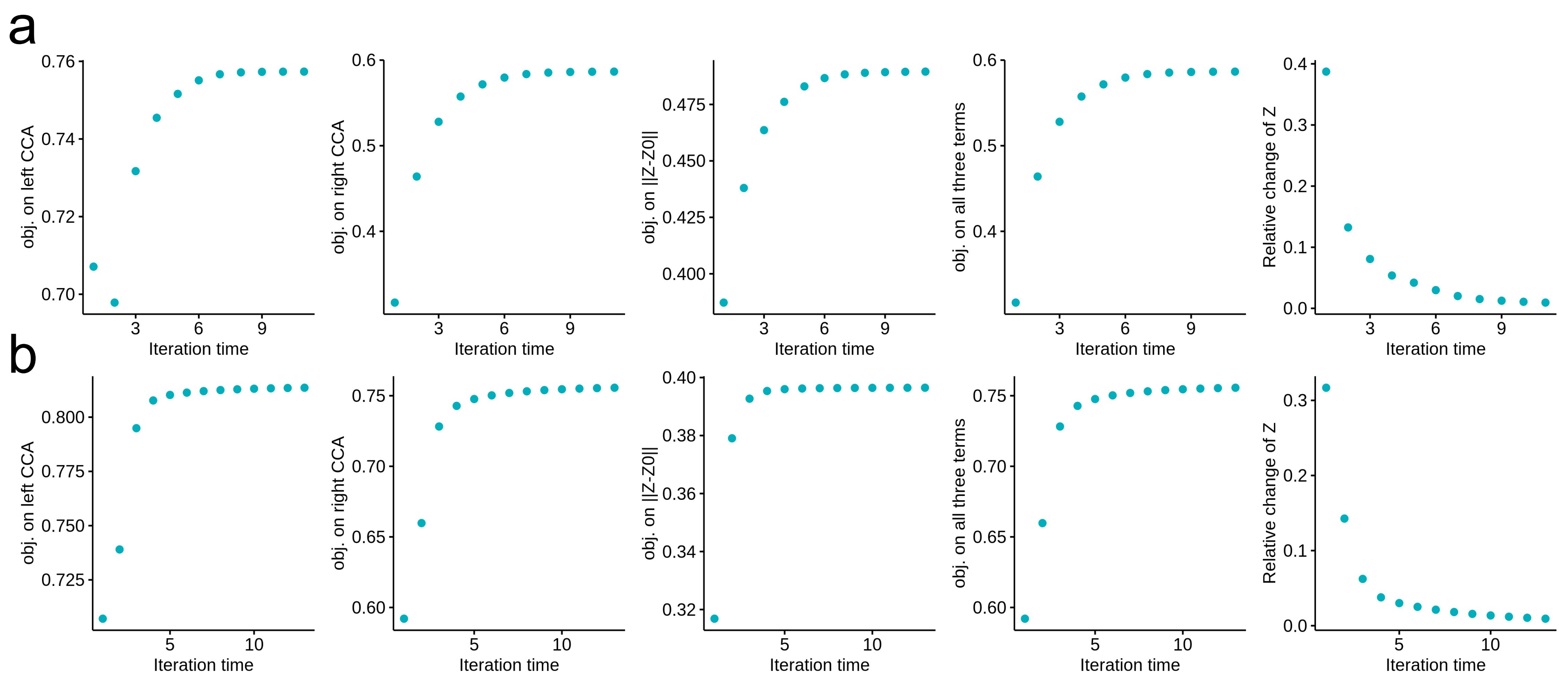

Fig. S15. Change of objective function cost over the iteration time. Results are shown for 2 benchmarking datasets used. From top to bottom is mouse retinal cell atlas (**a**) and bone marrow data (**b**). For each dataset, results are shown for cost of three terms separately, the total function cost, and the relative change of imputed gene score matrix Z over the iteration time.

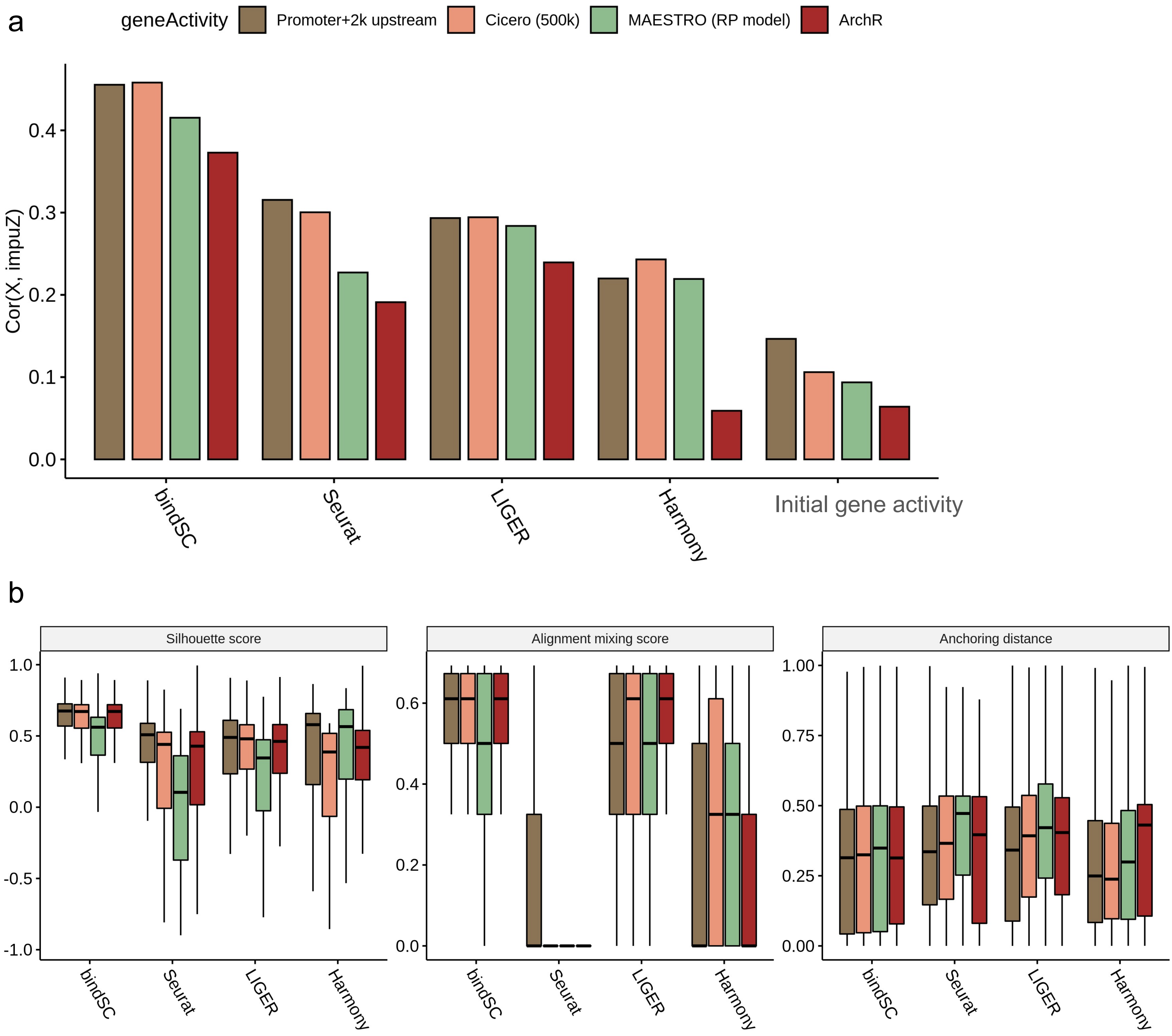

Fig. S16. Comparison of integration using initial modality fusion matrix $Z^{(0)}$ calculated by different models on retina data.

a) Pearson correlation (y-axis) between ground-truth co-assayed RNA levels and RNA levels imputed by bindSC, Seurat, LIGER and Harmony (x-axis) after integration, respectively. The initial gene activity matrix is also included as a baseline in the comparison. The correlation was calculated by treating the RNA profile matrices as vectors. Higher correlation denotes better estimation of gene expression information.

b) Integration metrics including silhouette score (left), alignment mixing score (middle), and anchoring distance (right) with different initial modality fusion matrix $Z^{(0)}$.
