# Supplementary material for: Bi-order multimodal integration of single-cell data
Source: Genome Biol. 2022 May 9;23:112. doi: 10.1186/s13059-022-02679-x (PMC9082907; doi:10.1186/s13059-022-02679-x)
Supplement: Supplementary file 2 — Additional file 2: Supplementary Note 1. Previous studies on multi-omics integration. Supplementary Note 2. Simulation Study. Supplementary Note 3. Effect of parameters on integration results. Supplementary Note 4. The iteration process of bindSC. Supplementary Note 5. Effect of initial fusion matrix on integration results. [file 13059_2022_2679_MOESM2_ESM.pdf]

Supplementary Notes for "Bi-order multimodal integration of single-cell data"

Jinzhuang Dou[1,2], jdou1@mdanderson.org
Shaoheng Liang[1,2], sliang3@mdanderson.org
Vakul Mohanty[1], vmohanty@mdanderson.org
Qi Miao[1], qmiao1@mdanderson.org
Yuefan Huang[1], yhuang15@mdanderson.org
Xuesen Cheng[3], xuesenc@bcm.edu
Sangbae Kim[3], Sangbae.Kim@bcm.edu
Jongsu Choi[3], Jongsu.Choi@bcm.edu
Yumei Li[3], yumeil@bcm.edu
Li Li[5], lli19@mdanderson.org
May Daher, MDaher@mdanderson.org
Rafet Basar[5], rbasar@mdanderson.org
Katayoun Rezvani[5], krezvani@mdanderson.org
Rui Chen[3,4], ruichen@bcm.edu
Ken Chen[1,6]. kchen3@mdanderson.org

[1]Department of Bioinformatics and Computational Biology, The University of Texas MD Anderson Cancer Center
[2] The authors contributed equally.
[3]Department of Molecular and Human Genetics, Baylor College of Medicine, Houston, TX, 77030, USA
[4]Verna and Marrs McLean Department of Biochemistry and Molecular Biology, Baylor College of Medicine, Houston, TX, 77030, USA
[5]Department of Stem Cell Transplantation and Cellular Therapy, The University of Texas MD Anderson Cancer Center, Houston, Texas
[6]Correspondence: kchen3@mdanderson.org

Supplementary Notes

Supplementary Note 1 Previous studies on multi-omics integration

A recent study [1] evaluated 14 single-cell batch-effect correction/integration methods, and recommended Harmony [2], LIGER [3], and Seurat3.0 [4]. In addition, we included four manifold-alignment based methods Panoma [5], SCOT [6], UnionCom [7] and MMD-MA [8] for comparison.

Harmony

Harmony [2] uses an iterative clustering approach to align cells from different batches. The algorithm first combines the batches and projects the data into a dimensionally reduced space using PCA. It then uses an iterative procedure to remove the multi-dataset specific effects. In our analysis, we ran Harmony within the Seurat3.0 based on the guide (http://htmlpreview.github.io/?https://github.com/immunogenomics/harmony/blob/master/docs/SeuratV3.html).

Seurat v3.0

Seurat [4] uses CCA to first compute the linear combinations of genes with the maximum correlation between batches and then adopts mutual nearest neighbor (MNN) to align the cells between datasets based on identified anchor cells. In our analysis, we used the Seurat package version 3.0 in the *R* language environment to perform multi-omics integration, adhering to the suggested integration workflow (https://satijalab.org/seurat/v3.2/atacseq_integration_vignette.html).

LIGER

LIGER [3] uses integrative non-negative matrix factorization (iNMF) to first learn a low-dimensional space where each gene is characterized by two sets of factors. The first set contains

dataset-specific factors, and the second contains shared factors. The shared factor space is then used to identify similar cell types across datasets by first constructing a shared factor neighborhood graph to connect cells with similar factor loading patterns. Joint clusters are then identified using the Louvain community detection algorithm. Thereafter, the factor loading quantiles are normalized using the largest data batch as a reference to achieve batch-correction. In our work, we followed the LIGER documentation (http://htmlpreview.github.io/?https://github.com/MacoskoLab/liger/blob/master/vignettes/Integrating_scRNA_and_scATAC_data.html). For preprocessing, we used the LIGER preprocessing functions, where we first selected genes with high variances. We then performed iNMF-based factorization using an alternating least squares algorithm, followed by data alignment using joint clustering and quantile alignment.

MMD-MA

MMD-MA performs multiomic data integration by optimizing the objective function with three components: 1) a maximum mean discrepancy term to make the differently measured points to have similar distributions in the latent space based on the kernel Gram matrices; 2) a distortion term to preserve the structure of the data between the input space and the latent space; and 3) a penalty term to ensure that distortion between the data in the original space and the data mapped to the low-dimensional space as small as possible. The transformation to the latent space is based on Gaussian kernel, and thus solely retains cell-cell distance information.

UnionCom

UnionCom first uses the cell-cell distance matrix of each dataset to represent its manifold. It then aligns the cells across single-cell multi-omics datasets by matching the distance matrices by an extended version of the unsupervised manifold alignment method GUMA. Finally, it projects the

distinct unmatched features across single-cell multi-omics datasets by matching the distance matrices via a matrix optimization method.

SCOT

Like UnionCom, SCOT (single-cell alignment using optimal transport) aims to preserve local geometry when aligning single-cell data. The algorithm achieves this by constructing a k-nearest neighbor graph for each dataset. SCOT uses Gromov-Wasserstein optimal transport to find a probabilistic coupling between the samples of each dataset. Finally. It uses the coupling matrix to project one single-cell dataset onto another through barycentric projection, thus aligning them.

Pamona

Pamona formulates the single cell multi-omics datasets as the partial manifold alignment problem and solves it under the partial Gromov-Wasserstein optimal transport framework. As the “partial” in its name suggested, it is more flexible in handling sample-specific cells than SCOT. The integration includes three steps: 1) constructs a weighted k-NN graph of cells of each dataset and computes the geodesic distance matrix of cells within each dataset; 2) calculates probabilistic coupling matrices of cells based on the partial Gromov-Wasserstein optimal transport; and 3) aligns cellular modalities with distinct unmatched features in a common low-dimensional space.

Supplementary Note 2 Simulation Study

Existing integration methods such as Seurat, LIGER, and Harmony rely on pre-aligning features across modalities, i.e., compressing cell-peak matrices obtained from scATAC-seq onto cell-gene-activity matrices based on reference genome annotations. BindSC further improves the integration by considering distal regulatory relations in a *de novo* fashion. . The ability of considering and refining intermodal feature interaction also allows bindSC to adapt more to the underlying biology and distinguish similar cell populations better than manifold alignment methods such as Matcher, MMD-MA, UnionCom, SCOT, and Pamona.

Under our formulation (**Methods**), **Z** has features (rows) aligned with **X** and cells (columns) aligned with **Y**. The introduction of **Z** enables bi-order alignment of the cells and the features, respectively.

We systematically simulated a series of datasets to assess how different combinations of proximal and distal regulatory elements affect the integration. In brief, 1,500 scATAC-seq peaks for 2,000 cells were simulated using Splatter [10] (Additional file 1: **Fig. S2a**). The cells were uniformly drawn into 3 types, resulting in $N = 668$, 701, and 631, respectively. Then, 500 gene expressions were calculated as the weighted mean of both proximal and distal peaks. Each gene is regulated by three proximal peaks and two distal peaks. We varied the relative strength of distal peaks, w, from 0.2 to 0.9, and the strength of proximal peaks is then $(1 - w)$. The gene activity matrix, initialized from peaks in gene bodies, was an unweighted sum among the proximal peaks (Additional file 1: **Fig. S2b**). Given the simulated scATAC-seq and scRNA-seq data, we used various methods to integrate the two datasets. A good approach should be able to generate co-

embeddings in which the cells from the two datasets are mixed according to their cell type identities. As shown in Additional file 1: **Fig. S2c**, bindSC successfully integrated the datasets under all the settings. Seurat maintained the overall population structure but was not able to mix the data well. Its performance further deteriorated as w increases. Similarly, Harmony was able to maintain the overall population structure but mis-assigned cells to incorrect clusters (mixing colors) as w increases. Most of the other methods failed to produce co-embeddings of the expected population structure. For example, UnionCom got confused when matching cell types of similar abundance and swapped cell types (blue and red in Additional file 1: **Fig. S2**c, $w = 0.7$).

We also found that the estimated matrix $\mathbf{Z}$ achieved a high correlation with the ground truth matrix within the first 5 iterations (Additional file 1: **Fig. S2d**). Notably, the correlation was initially worse for larger w (i.e., distal regulation dominant) but quickly improved as $\mathbf{Z}$ got updated. Although the real regulatory relations between ATAC peaks and gene expressions are considerably more complex and dynamic, this simple simulation experiment proves that bindSC has a clear edge over other methods in integrating modalities and inferring underlying regulatory relations across a range of conditions.

To further test the robustness of bindSC w.r.t. variable data dimensions, we varied the number of ATAC peaks from 1,000 to 10,000 while keeping the number of genes at 500. This simulated a range of two to 20-fold imbalance between the numbers of features in the two modalities. The results indicated the robustness of bindSC over the range of the parameters (Additional file1: **Fig. S2e**).

Supplementary Note 3 Effect of parameters $\boldsymbol{E}$, $\boldsymbol{\alpha}$ and $\boldsymbol{\lambda}$ on integration results

There are three hyperparameters in bindSC: 1) dimensionality E in the latent space, 2) the couple coefficient α representing the weight of initial modality fusion matrix $\boldsymbol{Z}^{(\mathbf{0})}$ and 3) the scale factor λ balancing the contribution of each modality. As a general suggestion, we recommend starting E with the minimal number of principle components (PCs) used in single modality clustering. In two benchmarking datasets, we tested a range of E (Additional file1: **Fig. S4i** on mouse retina data and Additional file 1: **Fig. S8e** on human bone marrow data). We noticed that performance of all methods is relatively robust to the choice of E when $E \geq 10$, except for LIGER on mouse retina data. As shown, bindSC outperformed all the other methods regardless of E.

We range α from 0 to 1 and λ from 0 to 1 with the step size 0.1. Selection of α and λ dependent on two integration metrics: 1) Silhouette score and 2) Alignment mixing score between two modalities. None of the metrics rely on labels and thus can be applied to new unlabeled data to determine the best value of the parameters. Additional file 1: **Fig. S14** show the value distribution of two integration metrics with different levels of α and λ on two benchmarking datasets used in this study. Overall, the silhouette and alignment scores are robust to α and λ, except for some specific cases with $\alpha = 1\ or\ 0$ and $\lambda = 1\ or\ 0$.

Supplementary Note 4 The iteration process of bindSC

With specified values of E, α and λ, bindSC repeats equations (4) to (7) in **Methods**, until $\|\boldsymbol{Z}_{\boldsymbol{i+1}} - \boldsymbol{Z}_{\boldsymbol{i}}\|_F^2 / \|\boldsymbol{Z}_{\boldsymbol{i}}\|_F^2 < \Delta$**,** where i is the iteration index and Δ is the termination condition specified by users (default = 0.01). Additional file1: **Fig. S15** shows the change of objective function cost over each step of the iteration. In our four benchmarking datasets, the iteration converged in less than 15 steps. In most cases, convergence plateaued within 5 iterations.

Supplementary Note 5 Effect of initial fusion matrix $Z^{(0)}$ on integration results

BindSC requires the initial modality fusion matrix $\boldsymbol{Z}^{(\mathbf{0})}$ as input to link $\boldsymbol{X}$ and $\boldsymbol{Y}$. It can be considered as the projection of $\boldsymbol{Y}$ to the feature space of $\boldsymbol{X}$. For integration of scRNA-seq and scATAC-seq data, the modality fusion matrix $\boldsymbol{Z}$ is usually derived from scATAC-seq profiles by summing reads in gene bodies plus upstream 2kb. We also considered other ways for initialization by1) aggregating reads in gene bodies plus co-accessible peaks within 500kb identified from Cicero [11], 2) aggregating reads with weighs inferred by the regulatory potential (RP) model from MAESTRO [12] and, 3) using the gene score model from ArchR [13]. To assess the performance of each gene score model, we run bindSC, Seurat, LIGER, and Harmony to integrate scATAC-seq and scRNA-seq data obtained from the same types and then evaluate the integration performance based on the correlation between ground truth and the imputed RNA profiles. Results for the mouse retina data are shown in Additional file1: **Fig. S16**. Note that including the gene score model from MAESTRO and that from ArchR did not improve the integration performance in our study.

Reference

1. Tran HTN, Ang KS, Chevrier M, Zhang X, Lee NYS, Goh M, Chen J: **A benchmark of batch-effect correction methods for single-cell RNA sequencing data.** *Genome biology* 2020, **21:**1-32.
2. Korsunsky I, Millard N, Fan J, Slowikowski K, Zhang F, Wei K, Baglaenko Y, Brenner M, Loh P-r, Raychaudhuri S: **Fast, sensitive and accurate integration of single-cell data with Harmony.** *Nature methods* 2019**:**1-8.
3. Rosenberg AB, Roco CM, Muscat RA, Kuchina A, Sample P, Yao Z, Graybuck LT, Peeler DJ, Mukherjee S, Chen W: **Single-cell profiling of the developing mouse brain and spinal cord with split-pool barcoding.** *Science* 2018, **360:**176-182.
4. Stuart T, Butler A, Hoffman P, Hafemeister C, Papalexi E, Mauck III WM, Hao Y, Stoeckius M, Smibert P, Satija R: **Comprehensive integration of single-cell data.** *Cell* 2019, **177:**1888-1902. e1821.
5. Cao K, Hong Y, Wan L: **Manifold alignment for heterogeneous single-cell multi-omics data integration using Pamona.** *bioRxiv* 2020.
6. Demetci P, Santorella R, Sandstede B, Noble WS, Singh R: **Gromov-Wasserstein optimal transport to align single-cell multi-omics data.** *BioRxiv* 2020.
7. Cao K, Bai X, Hong Y, Wan L: **Unsupervised Topological Alignment for Single-Cell Multi-Omics Integration.** *bioRxiv* 2020.
8. Liu J, Huang Y, Singh R, Vert J-P, Noble WS: **Jointly embedding multiple single-cell omics measurements.** *BioRxiv* 2019**:**644310.
9. Duren Z, Chen X, Zamanighomi M, Zeng W, Satpathy AT, Chang HY, Wang Y, Wong WH: **Integrative analysis of single-cell genomics data by coupled nonnegative matrix factorizations.** *Proceedings of the National Academy of Sciences* 2018, **115:**7723-7728.
10. Zappia L, Phipson B, Oshlack A: **Splatter: simulation of single-cell RNA sequencing data.** *Genome biology* 2017, **18:**1-15.
11. Pliner HA, Packer JS, McFaline-Figueroa JL, Cusanovich DA, Daza RM, Aghamirzaie D, Srivatsan S, Qiu X, Jackson D, Minkina A: **Cicero predicts cis-regulatory DNA interactions from single-cell chromatin accessibility data.** *Molecular cell* 2018, **71:**858-871. e858.
12. Wang C, Sun D, Huang X, Wan C, Li Z, Han Y, Qin Q, Fan J, Qiu X, Xie Y: **Integrative analyses of single-cell transcriptome and regulome using MAESTRO.** *Genome biology* 2020, **21:**1-28.
13. Granja JM, Corces MR, Pierce SE, Bagdatli ST, Choudhry H, Chang HY, Greenleaf WJ: **ArchR is a scalable software package for integrative single-cell chromatin accessibility analysis.** *Nature genetics* 2021, **53:**403-411.
