# Supplementary material for: Bi-order multimodal integration of single-cell data
Source: Genome Biol. 2022 May 9;23:112. doi: 10.1186/s13059-022-02679-x (PMC9082907; doi:10.1186/s13059-022-02679-x)
Supplement: Supplementary file 4 — Additional file 4. Review history. [file 13059_2022_2679_MOESM4_ESM.docx]

Review History

**First round of review**

**Reviewer 1**

**Were you able to assess all statistics in the manuscript, including the appropriateness of statistical tests used?**

No

**Comments to author:**

In this work, Dou and colleagues introduce bindSC, a canonical correlation analysis (CCA) framework for integrating unpaired single cell data sampled from the same population but from disparate modalities such as scRNA-seq and scATAC-seq. bindCA introduces a projection matrix Z that acts as a projection of one of the modalities into the space of the other (ie a representation of every cell of modality B in the feature space of A). It further introduces cell and feature level projection matrices down to an E-dimensional reduced embedding, with an overall loss function that attempts to maximize the correlation between the embeddings of each data modality. The novelty is attempting to maximize this agreement both with respect to the embedding of the cells and the features simultaneously with Z that links the feature space of one domain to another.

To demonstrate the efficacy of their method, the authors generate scRNA-seq and scATAC-seq from the mouse retina that provides paired measurements, giving a sense of a ground truth. On this dataset, they show their method outperforms existing approaches (Seurat, Liger, Harmony). They further apply their method to a diverse array of additional data including Cite-seq and CyTOF+scRNA-seq where they find a subset of CAR NK cells.

Overall, the method is promising and the empirical results good. However, I have three main concerns that warrant further investigation: (i) missing comparisons to similar methods in the field, (ii) unexpectedly poor performance of existing methods, and (iii) no benchmarking in the case of imbalanced cellular populations. Detailed comments below.

\# Major

There are two methods that are missing from the algorithms benchmarked against: coupled NMF [1] and MAESTRO [2], commonly used for the integrative analysis of scRNA + scATAC. Similarly, can the authors confirm whether they use "Signac" [3] for the integration aspect of Seurat?
[1] https://www.pnas.org/content/115/30/7723
[2] https://genomebiology.biomedcentral.com/articles/10.1186/s13059-020-02116-x
[3] https://satijalab.org/signac/index.html

When benchmarking against other algorithms, the suggestion is that Liger and Harmony, and to a lesser extent Seurat, do not work at all for this integration task (they typically collapse many clusters in one modality into a single cluster in the other). However, this contradicts earlier work that shows these methods may be run successfully. Can the authors check their implementations for correctness and if no issues found attempt to explain why the other methods fail so badly on this dataset?

bindSC has several hyperparameters that undergo optimization (section "Parameter optimization"). While I think it is fantastic to have a principled manner to tune hyperparameters, it appears the other methods benchmarked against are run with default parameter settings, which goes against the guidelines in this journal for benchmarking computational biology software [1]. Can the authors demonstrate the benchmarking results when the other algorithms are subject to similar tuning?

[1] https://genomebiology.biomedcentral.com/articles/10.1186/s13059-019-1738-8

The main benchmarking portion of the paper (Figure 2) relies on paired modality measurement as a ground truth (which makes sense). However, a natural consequence of this is that the number of cells in each modality is perfectly balanced (and beyond this, the distribution of cells across clusters is matched). While this is an assumption of bindSC, it likely doesn't reflect real unpaired data due to sampling and technical limitations. Can the authors create imbalanced datasets in silico (ie by down sampling clusters to different proportions in each modality) and demonstrate the performance of their method (and others)? Similarly, can the authors demonstrate that their benchmarking results are robust to multiple clusterings of the data, since there is more than one way to cluster a given dataset?

Fig 2C is presented as evidence that existing methods that reduce to gene activity do not learn meaningful representations of the cells by eyeballing the UMAP and saying "the cell types became even harder to delineate". This is not a quantitative description. Can the authors be more quantitative here and e.g. calculate ARI wrt scRNA-seq cell types? This issue comes up again in Figure 3b "CD8+ and CD4+ T cells were partly blended together in the RNA data but separated clearly in the protein data."

Similarly, in several places the authors criticize existing approaches for pre-setting the gene<->region relationship between integrations, e.g.
- "Existing integration methods such as Seurat, LIGER, and Harmony completely rely on require pre-aligning features across modalities, i.e., compressing cell-peak matrices obtained from scATAC-seq onto cell-gene-activity matrices based on reference genome annotations. BindSC overcomes that restriction"
- "proximity to nearest genes, an ad hoc that most current programs perform to produce so-called gene activity matrices, the cell types became even harder to delineate"
However, their own method essentially relies on these approaches to initialize the feature mapping matrix Z. Not only this, but the loss has a term that explicitly penalizes Z moving too far from its initialization via |Z-Z_0|. I would ask that the authors:
1. Tone down the language critiquing the other methods when their own method similarly relies on this
2. Quantify how far Z moves from its initialization Z_0 through correlating the two or similar to in turn find out how much "free alignment amongst features to be established from data." as is claimed there really is. The fact that |Z-Z_0| is penalized suggests this won't be the case.

The authors write (about integrating protein expression and scRNA-seq) "Notice that this task cannot be achieved by any of the existing tools because the mRNA and protein expression levels derived from the same genes are not well correlated, due to complex post-transcriptional modifications and technological limitations". However, this is immediately contradicted via "Unsupervised clustering of the RNA profiles revealed cell types largely consistent with those in the protein profiles". Can the authors apply the other algorithms to this dataset, since it seems it is indeed feasible?

\# Minor

Does E need to be the same for both features and cells?

The authors write "Thus, they tend to match cell types of similar abundances instead of similar biology and suffer misalignment for cell types of similar abundance, especially rare cell types." -> does the second part of this sentence not contradict the first?

In the methods the authors start referring to the feature vs cell parts of the loss as "modality". This doesn't seem like a good choice of words here given the previous use as e.g. scRNA vs scATAC.

**Reviewer 2**

**Were you able to assess all statistics in the manuscript, including the appropriateness of statistical tests used?**

Yes

**Comments to author:**

In this manuscript Jinzhuang Dou et al. extended regular Canonical Correlation Analysis (CCA) to introduce bi-CCA which could simultaneously and interactively align both the rows and columns (features and cells in single cell) data matrix. They validated their method with with ground truth multiome data which measures two single cell modality from the same cell and showed that it is more accurate than existing approaches in generating multimodal co-embedding. Given the rise of large single cell multimodal data sets generated from the same sample this is an important work where we can use the integrated co-embedding to discover new biology. However, I would request further clarification on some issues before I can recommend it for publication


- Using gene score by summing reads in gene bodies is a crude measure of actual gene expression. In the single cell ATAC-seq analysis tool ArchR (Nat Genet 53, 403-411 (2021)), authors have introduced a more robust model to calculate the gene score. If this is used for bi-CCA how the performance is improved? Also, if this model is used in the other methods in Seurat 3 or Harmony do these methods still perform worse than bindSC?

- It was not very obvious to me (apologies if I missed it) from the formulation ow alpha can be adjusted to take into account the uncertainty in the gene score model, i.e. for different gene score models where the accuracy varies significantly how this can be addressed using the alpha hyper parameter?

- If we want to integrate two modalities where the features are not directly shared how bindSC would perform? For eg. in case of integrating snATAC-seq and Spatial transcriptomics data, how this can be achieved?

- How bindSC scales to large number of cells. The authors of this paper used 27,181 metal muscle cells from human metal cell atlas to demonstrate applicability of their method. But how bindSC performs if applied to all cells (nearly 800,000)? As the datasets would only be growing in near future it is important that the authors benchmark their method with large datasets.

- Also can bindSC implementation work on data structures that supports out of core computation? like HDF5 or zarr?


- It was not immediately clear to me how the multi-view in joint embedding would be interpreted. In the multi-view we can project the gene expression (or gene score for snATAC-seq) but we cannot project peak opening for the snRNA-seq datasets. So how an user would use and interpret the multi-view in the joint embedding?

**Authors Response**

**Point-by-point responses to the reviewers’ comments:**

Reviewer #1: In this work, Dou and colleagues introduce bindSC, a canonical correlation analysis (CCA) framework for integrating unpaired single cell data sampled from the same population but from disparate modalities such as scRNA-seq and scATAC-seq. bindCA introduces a projection matrix Z that acts as a projection of one of the modalities into the space of the other (ie a representation of every cell of modality B in the feature space of A). It further introduces cell and feature level projection matrices down to an E-dimensional reduced embedding, with an overall loss function that attempts to maximize the correlation between the embeddings of each data modality. The novelty is attempting to maximize this agreement both with respect to the embedding of the cells and the features simultaneously with Z that links the feature space of one domain to another.

To demonstrate the efficacy of their method, the authors generate scRNA-seq and scATAC-seq from the mouse retina that provides paired measurements, giving a sense of a ground truth. On this dataset, they show their method outperforms existing approaches (Seurat, Liger, Harmony). They further apply their method to a diverse array of additional data including Cite-seq and CyTOF+scRNA-seq where they find a subset of CAR NK cells.

Overall, the method is promising and the empirical results good. However, I have three main concerns that warrant further investigation: (i) missing comparisons to similar methods in the field, (ii) unexpectedly poor performance of existing methods, and (iii) no benchmarking in the case of imbalanced cellular populations. Detailed comments below.

\# Major

**1.1** There are two methods that are missing from the algorithms benchmarked against: coupled NMF [1] and MAESTRO [2], commonly used for the integrative analysis of scRNA + scATAC. Similarly, can the authors confirm whether they use "Signac" [3] for the integration aspect of Seurat?
[1] [https://urldefense.com/v3/__https://www.pnas.org/content/115/30/7723__;!!PfbeBCCAmug!zqj_aT5nUArIrqHk_gHPqOLp-ZjjT53be2RfREHwGnLpeTGBsOZVUOx8_Aa8_fyNRQ$](https://urldefense.com/v3/__https:/www.pnas.org/content/115/30/7723__;!!PfbeBCCAmug!zqj_aT5nUArIrqHk_gHPqOLp-ZjjT53be2RfREHwGnLpeTGBsOZVUOx8_Aa8_fyNRQ$)
[2] [https://urldefense.com/v3/__https://genomebiology.biomedcentral.com/articles/10.1186/s13059-020-02116-x__;!!PfbeBCCAmug!zqj_aT5nUArIrqHk_gHPqOLp-ZjjT53be2RfREHwGnLpeTGBsOZVUOx8_AanDfC7fg$](https://urldefense.com/v3/__https:/genomebiology.biomedcentral.com/articles/10.1186/s13059-020-02116-x__;!!PfbeBCCAmug!zqj_aT5nUArIrqHk_gHPqOLp-ZjjT53be2RfREHwGnLpeTGBsOZVUOx8_AanDfC7fg$)
[3] [https://urldefense.com/v3/__https://satijalab.org/signac/index.html__;!!PfbeBCCAmug!zqj_aT5nUArIrqHk_gHPqOLp-ZjjT53be2RfREHwGnLpeTGBsOZVUOx8_AYV45M1iA$](https://urldefense.com/v3/__https:/satijalab.org/signac/index.html__;!!PfbeBCCAmug!zqj_aT5nUArIrqHk_gHPqOLp-ZjjT53be2RfREHwGnLpeTGBsOZVUOx8_AYV45M1iA$)

***Response:***

*MAESTRO provides a regulatory potential (PR) model to calculate gene activity scores and uses CCA model to integrate scRNA-seq and scATAC-seq. Our supplementary material included integration results produced by bindSC, Seurat, LIGER, and Harmony using gene activity scores provided by MAESTRO (see new Additional file1:* ***Fig. S16****. and Additional file2:* ***Supplementary Note 5****). It shows that using MAESTRO scores did not improve integration results, compared with using Seurat gene activity scores. This highlighted the challenge of performing de novo gene regulation network inference from scATAC-seq data. We have now added the following statement on page 24 (section* ***Discussion****)* *to highlight this point.*

*“However, gene activity scores generated by them did not improve integration results in our benchmarking experiments (Additional file2:* ***Supplementary Note 5*** *and Additional file1:* ***Fig. S16****). This highlights the challenge of performing de novo gene regulation network inference from scATAC-seq data.”*

*We also ran coupled NMF with pre-calculated peak-gene interaction matrix (i.e.,* ***Z****_0_) in the same manner as we did for bindSC and other methods on mouse retina data. Coupled NMF only outputs joint clustering IDs and does not provide coordinates of cells in its co-embeddings (https://github.com/SUwonglab/CoupledNMF). Thus, it was not feasible for us to show integration results directly on a UMAP. Instead, we calculated label transfer accuracy based on clustering consistency on mouse retina data. The overall label transfer accuracy was only 0.25 (****Fig. R1****), worse than those of bindSC (0.75) and the other three methods (Seurat: 0.51, LIGER: 0.26, and Harmony: 0.31;* ***Fig. 2****). Given CoupledNMF could not provide coordinates of co-embeddings required for downstream evaluation, we plan not to include the CoupledNMF results in our manuscript.*

***Fig. R1*** ***Consistency between cell types inferred computationally from ATAC profiles by coupled NMF and the ground truth.*** *Darkness of the dots corresponds to degree of consistency with the ground truth, while size of the dots the fraction of cells per row over the columns. The overall accuracy is 0.25.*

*Yes, we used "Signac" for the integration aspect of Seurat. Specifically, we performed integration following the Vignette at* [*https://satijalab.org/seurat/archive/v3.2/atacseq_integration_vignette.html*](https://satijalab.org/seurat/archive/v3.2/atacseq_integration_vignette.html)*. Seurat v3 and Signac share the same backends on constructing gene activity matrix (CreateGeneActivityMatrix in Seurat vs. GeneActivity in Signac) and aligning two modalities using CCA (FindTransferAnchors + TransferData in Seurat vs. runCCA in Signac).*

**1.2** When benchmarking against other algorithms, the suggestion is that Liger and Harmony, and to a lesser extent Seurat, do not work at all for this integration task (they typically collapse many clusters in one modality into a single cluster in the other). However, this contradicts earlier work that shows these methods may be run successfully. Can the authors check their implementations for correctness and if no issues found attempt to explain why the other methods fail so badly on this dataset?

***Response:***

*We have double-checked our analyses, which was performed by calling functions implemented by these user-friendly packages in a straightforward way. We did not find errors in our analyses. We provided the scripts for running four methods on these two benchmarking experiments from community scrutiny at* [*https://htmlpreview.github.io/?https://github.com/KChen-lab/bindSC/blob/master/vignettes/method_eval/method_eval.retina.html*](https://htmlpreview.github.io/?https://github.com/KChen-lab/bindSC/blob/master/vignettes/method_eval/method_eval.retina.html) *(mouse retina data) and*

[*https://htmlpreview.github.io/?https://github.com/KChen-lab/bindSC/blob/master/vignettes/method_eval/method_eval.citeseq.html*](https://htmlpreview.github.io/?https://github.com/KChen-lab/bindSC/blob/master/vignettes/method_eval/method_eval.citeseq.html) *(human bone marrow data).*

*The lack of good performance of Seurat, LIGER, and Harmony on the data can be well explained by the substantially reduced molecular diversity in the feature-reduced data matrices, as compared with the original data matrices, before they were analyzed by these tools. The design of bindSC enables us to incorporate original features so as not to throw away a substantial part of data.*

*We provided following explanation in the revised manuscript for the mouse BC data on page 11 (section* ***Results; Fig. 2b vs 2c****):*

*“We then examined the ATAC profiles and found that cells in the same cell-types were largely clustered together (ARI = 0.71) although were not as distinctive. When we reduced the ATAC data to gene resolution based on proximity to nearest genes, the cell types became harder to delineate (ARI = 0.23;* ***Fig. 2c****), indicating that the gene activity transformation loses information.”*

*and for the human bone marrow data on page 16 (section* ***Results; Fig. 3b vs 3c****):*

*“In contrast, the gene expression levels of the 25 RNAs encoding the 25 proteins lacked delineating power and could not yield meaningful classification (ARI = 0.09;* ***Fig. 3c****).”*

**1.3** bindSC has several hyperparameters that undergo optimization (section "Parameter optimization"). While I think it is fantastic to have a principled manner to tune hyperparameters, it appears the other methods benchmarked against are run with default parameter settings, which goes against the guidelines in this journal for benchmarking computational biology software [1]. Can the authors demonstrate the benchmarking results when the other algorithms are subject to similar tuning?

[1] [https://urldefense.com/v3/__https://genomebiology.biomedcentral.com/articles/10.1186/s13059-019-1738-8__;!!PfbeBCCAmug!zqj_aT5nUArIrqHk_gHPqOLp-ZjjT53be2RfREHwGnLpeTGBsOZVUOx8_AZT9cs-Sw$](https://urldefense.com/v3/__https:/genomebiology.biomedcentral.com/articles/10.1186/s13059-019-1738-8__;!!PfbeBCCAmug!zqj_aT5nUArIrqHk_gHPqOLp-ZjjT53be2RfREHwGnLpeTGBsOZVUOx8_AZT9cs-Sw$)

***Response:***

*In the integration function in Seurat (runCCA), the only adjustable parameter is “num.cc” (number of canonical vectors to calculate. In LIGER, there are two key parameters: 1) the number of matrix factor* $k$ *and dataset-specific factor* $\lambda$ *(Liu et al., 2020). The dataset-specific factor is set to 5 based on the author’s suggestion when aligning two different modalities. In Harmony, there is an option “dim.use” (dimension of PCA used for integration). Thus, the dimension of the latent space* $k$ *is the most important factor influencing the integration.*

*In our current manuscript, we chose the same* $k$ *for all the methods (*$k$ *= 15 for mouse retina data and* $k$ *= 10 for human bone marrow data;* ***Supplementary Table 1****) to ensure fairness of comparison.*

*To further explore parameter tuning, we tested a range of* $k$ *on two benchmarking datasets (new* *Additional file1:* ***Fig. S4i*** *on mouse retina data;* *new* *Additional file1:* ***Fig. S8e*** *on human bone marrow data). We noticed that performance of all methods is relatively robust to the choice of* $k$ *when* $k$ $\geq10$*, except for LIGER on mouse retina data. As shown, bindSC outperformed all the other methods regardless of* $k.$ *We have added these results in Additional file2:* ***Supplementary Note 3*** *and new* ***Fig. S4i*** *and* ***Fig. S8e****.*

*Reference*

*Liu, Jialin, et al. "Jointly defining cell types from multiple single-cell datasets using LIGER." Nature protocols 15.11 (2020): 3632-3662.*

**1.4** The main benchmarking portion of the paper (Figure 2) relies on paired modality measurement as a ground truth (which makes sense). However, a natural consequence of this is that the number of cells in each modality is perfectly balanced (and beyond this, the distribution of cells across clusters is matched). While this is an assumption of bindSC, it likely doesn't reflect real unpaired data due to sampling and technical limitations. Can the authors create imbalanced datasets in silico (ie by down sampling clusters to different proportions in each modality) and demonstrate the performance of their method (and others)? Similarly, can the authors demonstrate that their benchmarking results are robust to multiple clusterings of the data, since there is more than one way to cluster a given dataset?

***Response:***

*This is a good idea. We have simulated two common scenarios of imbalanced distributions using the mouse retina data: 1) removing 50% of the cells in the five major cell types (i.e., BC1, BC2, BC3, BC4, BC5) in the snATAC data while keeping the snRNA data intact, and 2) removing all the cells in the five minor cell types (i.e., BC6, BC7, BC8, BC9, BC10) in the snATAC data while keeping the snRNA data intact (new Additional file1:* ***Fig. S4d****).*

*In scenario 1, there were little difference in the results produced by all of the methods, compared with using the full datasets (new Additional file1:* ***Fig. S4e-f*** *vs* *original* ***Fig. 2f****)****.*** *In scenario 2, we still observed good alignment of BC1-5 in the UMAPs generated by the bindSC co-embedding, although some smearing started to appear between the major and the minor cell types. Other programs (particularly LIGER) also had worsened alignment in the UMAPs generated from the co-embeddings (Additional file1:* ***Fig. S4g)****. Nonetheless, bindSC achieved the best label transfer accuracy (Additional file1:* ***Fig. S4f,h****) among all the methods in both scenarios, indicating its robustness to imbalanced datasets. We have added following statements on page 12 (section* ***Results****).*

*“To further examine bindSC’s performance on scenarios where cell populations have imbalanced abundance between two modalities, we generated two datasets: 1) removing 50% of cells in the top five major cell types (i.e., BC1, BC2, BC3, BC4, BC5) in the snATAC data while keeping the snRNA data intact, and 2) removing the top five minor cell types (i.e., BC6, BC7, BC8, BC9, BC10) in the snATAC data while keeping the snRNA-seq data intact (Additional File 1:* ***Fig. S4d****). The label transfer accuracy of bindSC was similar with that from the full paired profiles, indicating bindSC alignment is robust on imbalanced datasets. Again, bindSC had the best performance among all methods in these two scenarios (Additional File 1:* ***Fig. S4e-h****).”*

*We also have added these in new Additional file1:* ***Fig. S4e-h****.*

*The benchmarking metrics here (including label transferring, Silhouette score, Alignment mixing score, Anchoring distance) do not depend on clustering results, as they were derived based on barcoded cell correspondence available in these co-assayed data.*

**1.5** Fig 2C is presented as evidence that existing methods that reduce to gene activity do not learn meaningful representations of the cells by eyeballing the UMAP and saying "the cell types became even harder to delineate". This is not a quantitative description. Can the authors be more quantitative here and e.g. calculate ARI wrt scRNA-seq cell types? This issue comes up again in Figure 3b "CD8+ and CD4+ T cells were partly blended together in the RNA data but separated clearly in the protein data."

***Response:***

*Thanks for the suggestions, we have added ARI metrics on the top right corners of the revised* ***Fig. 2b-c*** *and* ***Fig. 3a-c****. We have also added the ARI information on page 11 (section* ***Results****):*

*“We then examined the ATAC profiles and found that cells in the same cell-types were largely clustered together (ARI = 0.71) although were not as distinctive. When we reduced the ATAC data to gene resolution based on proximity to nearest genes, the cell types became harder to delineate (ARI = 0.23;* ***Fig. 2c****), indicating that the gene activity transformation loses information.”*

*and on page 16 (section R****esults****):*

*“CD8+ and CD4+ T cells were partly blended together in the RNA data (ARI = 0.43) but separated clearly in the protein data (ARI = 0.82). On the other hand, conventional dendritic cells (cDC2) were separated from other clusters in the RNA profiles but were intermixed with other cell types in the protein profile. In contrast, the gene expression levels of the 25 RNAs encoding the 25 proteins lacked delineating power and could not yield meaningful classification (ARI = 0.09;* ***Fig. 3c****).”*

**1.6** Similarly, in several places the authors criticize existing approaches for pre-setting the gene<->region relationship between integrations, e.g.

- "Existing integration methods such as Seurat, LIGER, and Harmony completely rely on require pre-aligning features across modalities, i.e., compressing cell-peak matrices obtained from scATAC-seq onto cell-gene-activity matrices based on reference genome annotations. BindSC overcomes that restriction"
- "proximity to nearest genes, an ad hoc that most current programs perform to produce so-called gene activity matrices, the cell types became even harder to delineate"

However, their own method essentially relies on these approaches to initialize the feature mapping matrix Z. Not only this, but the loss has a term that explicitly penalizes Z moving too far from its initialization via |Z-Z_0|.

I would ask that the authors:

    1. Tone down the language critiquing the other methods when their own method similarly relies on this
    2. Quantify how far Z moves from its initialization Z_0 through correlating the two or similar to in turn find out how much "free alignment amongst features to be established from data." as is claimed there really is. The fact that |Z-Z_0| is penalized suggests this won't be the case.

***Response:***

*Thanks for the suggestions. We have toned down our critiques of other tools on page 11 (section* ***Results****) in the main text and updated as:*

*“When we reduced the ATAC data to gene resolution based on proximity to nearest genes, the cell types became harder to delineate (ARI = 0.23;* ***Fig. 2c****), indicating that the gene activity transformation loses information.”*

*We have also updated statements on page 5 in Additional file 2:* ***supplementary Note*** *2 as:*

*“Existing integration methods such as Seurat, LIGER, and Harmony rely on pre-aligning features across modalities, i.e., compressing cell-peak matrices obtained from scATAC-seq onto cell-gene-activity matrices based on reference genome annotations. BindSC further improves the integration by considering distal regulatory relations in a de novo fashion.”*

*In our original manuscript, we have quantified the difference between updated* ***Z*** *and the gold standard (measured based on matrix correlation) during the iteration for mouse retina data (****Fig. 2g****) and human bone marrow data (****Fig. S8d****), respectively. In the revised version, as suggested, we also quantified the difference between updated* ***Z*** *and* $\boldsymbol{Z}^{\boldsymbol{(0)}}$*(the upper panel in new* ***Fig. 2g*** *and* *new* ***Fig. S8d****). It shows that the updated* ***Z*** *became closer to the gold-standard and farther away from* $\boldsymbol{Z}^{\boldsymbol{(0)}}$ *after performing bi-CCA iterations, even when the penalized term was included. Although the penalty term discourages fluctuation of* ***Z*** *due to noise, when enough evidence in the data suggests against* $\boldsymbol{Z}^{\boldsymbol{(0)}}$*, bindSC will make proper adjustments. We have included the following statements in the revised manuscript on page 12 (section* ***Results****):*

*“After 3 iterations, the R value increased to 0.5, meanwhile the value between imputed and the initial profile decreased to as low as 0.2, indicating the power of associating full peak profiles to genes in a de novo fashion, rather than utilizing reduced profiles (****Fig. 2g****).”*

*and on page 17 (section* ***Results****) as:*

*“The imputed profile has high correlation with the true protein levels (Pearson’s R = 0.6) and low correlation with the initial gene scores (Pearson’s R < 0.3) (Additional file 1:* ***Fig. S8d****), again indicating the power of associating two modalities de novo.”*

**1.7** The authors write (about integrating protein expression and scRNA-seq) "Notice that this task cannot be achieved by any of the existing tools because the mRNA and protein expression levels derived from the same genes are not well correlated, due to complex post-transcriptional modifications and technological limitations". However, this is immediately contradicted via "Unsupervised clustering of the RNA profiles revealed cell types largely consistent with those in the protein profiles". Can the authors apply the other algorithms to this dataset, since it seems it is indeed feasible?

***Response:***

*Our previous description on page 14 may have caused some misunderstanding. We were referring to 3 sets of clustering results deriving from 3 different sets of genes and proteins, as shown in* ***Fig. 3a, b and c.*** *What we meant is that the UMAPs in Fig. 3a, deriving from protein and 3b, deriving from 3,000 mRNAs were consistent. However, the UMAP in Fig. 3c, deriving from the 25 mRNAs that directly code the 25 proteins, was substantially inconsistent because the levels of these 25 mRNAs are not well correlated with those of the proteins, as exemplified in Fig.* ***3h****.*

*To avoid confusions, we have modified related descriptions on pages 14-17 (section* ***Results:*** *Integration of single-cell RNA and epitope expression data).*

*We have already applied other methods in this dataset (see Results in* ***Fig. 3f****).*

\# Minor

Does E need to be the same for both features and cells?

***Response:***

*It is not necessary.*

The authors write "Thus, they tend to match cell types of similar abundances instead of similar biology and suffer misalignment for cell types of similar abundance, especially rare cell types." -> does the second part of this sentence not contradict the first?

***Response:***

*We intended to express that because the matching is based on the (ordering of) abundance, when some cell types are of similar abundance, they are indistinguishable by those methods. We have modified our statement as following on page 5 (section* ***Background****) to avoid confusions:*

*“Thus, they may misalign cell types of similar abundance instead of similar biology, especially rare cell types.”*

In the methods the authors start referring to the feature vs cell parts of the loss as "modality". This doesn't seem like a good choice of words here given the previous use as e.g. scRNA vs scATAC.

***Response:***

*Our approach is designed to align two modalities, which may include scRNA vs scATAC, scRNA vs protein, or scRNA vs spatial transcriptomics. To highlight the general use of bi-CCA, we use “modality” rather than specific molecular profile here.*

Reviewer #2: In this manuscript Jinzhuang Dou et al. extended regular Canonical Correlation Analysis (CCA) to introduce bi-CCA which could simultaneously and interactively align both the rows and columns (features and cells in single cell) data matrix. They validated their method with ground truth multiome data which measures two single cell modality from the same cell and showed that it is more accurate than existing approaches in generating multimodal co-embedding. Given the rise of large single cell multimodal data sets generated from the same sample this is an important work where we can use the integrated co-embedding to discover new biology. However, I would request further clarification on some issues before I can recommend it for publication

**2.1** - Using gene score by summing reads in gene bodies is a crude measure of actual gene expression. In the single cell ATAC-seq analysis tool ArchR (Nat Genet 53, 403-411 (2021)), authors have introduced a more robust model to calculate the gene score. If this is used for bi-CCA how the performance is improved? Also, if this model is used in the other methods in Seurat 3 or Harmony do these methods still perform worse than bindSC?

***Response:***

*In the revised manuscript, we have included new results using gene score matrixes initialized by ArchR (new Additional file1:* ***Fig. S16****; Additional file2:* ***Supplementary Note 5****). In the original manuscript, we included results using gene score matrixes initialized by MAESTRO and Cicero. As shown in our evaluation, gene score models from ArchR and MAESTRO did not improve the integration results for any of the methods in our benchmarking experiments. This highlights the challenge of performing de novo gene regulation network inference from scATAC-seq data. In all the cases we examined, bindSC outperformed other methods with clear margins. We have added these updates in Additional file2:* ***Supplementary Note 5****.*

**2.2**- It was not very obvious to me (apologies if I missed it) from the formulation ow alpha can be adjusted to take into account the uncertainty in the gene score model, i.e. for different gene score models where the accuracy varies significantly how this can be addressed using the alpha hyper parameter?

***Response:***

*We have a minor modification on equations to make them clearer. As shown in Equation (6), our approach uses* $\alpha$ *to adjust the contribution of initial modality fusion matrix* $\boldsymbol{Z}^{\boldsymbol{(0)}}$*:*

$\left( \boldsymbol{Z} \right):=(1-\alpha)\left\{ \frac{1-\lambda}{n_{l}}\boldsymbol{XU}\boldsymbol{S}^{'}+ \frac{\lambda}{n_{\boldsymbol{r}}}\boldsymbol{T}\boldsymbol{V}^{'}\boldsymbol{Y} \right\}+ {\alpha\boldsymbol{Z}}^{(\boldsymbol{0})}$*. (Equation 6)*

*When* $\alpha$ *is larger,* $\boldsymbol{Z}^{\boldsymbol{(0)}}$ *has a larger weight. Thus,* $\alpha$ *should be set higher, when* $\boldsymbol{Z}^{\boldsymbol{(0)}}$ *is more accurate. To help choose an* $\alpha$*, we designed two metrics (silhouette score and alignment mixing score, see Parameter optimization section in* ***Methods****) to assess the integration performance in an unsupervised manner. They do not rely on cell type labels or cell-cell correspondence and thus can be applied to any new unlabeled data. One can try a range of* $\alpha\in[0,1]$ *to find the best choice.*

*Additional file2:* ***Supplementary Note 3*** *and Additional file1:* ***Fig. S14*** *illustrate how* $\alpha$ *can be adjusted on the two benchmarking datasets (mouse retina data and human bone marrow). It is worth noting that the performance of bindSC is relatively robust to the selection of* $\alpha$ *on these two datasets.*

*To further illustrate this selection procedure for* $\boldsymbol{Z}^{\boldsymbol{(0)}}$ *of different accuracy, we added Gaussian noise to the original* $\boldsymbol{Z}^{\boldsymbol{(0)}}$*, creating two scenarios representing moderate and high noise levels.* ***Fig. R2*** *shows the relationship between* $\alpha$ *and silhouette/alignment mixing score under these scenarios. When there is moderate noise (green line), users can select* $\alpha=0.1\sim0.2$*. When the noise level is very high (blue line),* $\alpha\to0$ *achieves the best result. This selection procedure has been implemented in current bindSC package named BiCCA_para_opt. (Details can be seen in the Stage3: Parameter optimization in*

[*https://htmlpreview.github.io/?https://github.com/KChen-lab/bindSC/blob/master/vignettes/mouse_retina/retina.html*](https://htmlpreview.github.io/?https://github.com/KChen-lab/bindSC/blob/master/vignettes/mouse_retina/retina.html)*). We recommend users to use it to determine* $\alpha$*. We have added on page 37 (section* ***Methods****) the following statement:*

*“On any dataset, the optimal alpha can be determined using the BiCCA_para_opt functions in BindSC”.*

***Fig. R2.*** $\boldsymbol{\alpha}$ ***reduces the contribution of initial fusion matrix*** $\boldsymbol{Z}^{\boldsymbol{(0)}}$ ***when*** $\boldsymbol{Z}^{\boldsymbol{(0)}}$ ***is of low accuracy.*** *To model uncertainty level of the initial fusion matrix, we generated* $\boldsymbol{Z}_{0}^{noise}$ *on mouse retina data by adding Gaussian noise of a moderate level (green line) and a high level (blue line) to the originalfusion matrix (red line). We then ran bindSC with* $\alpha$ *ranging from 0 to 1 (x-axis) and reported the silhouette score (y-axis) and alignment score in each scenario. A higher score indicates better performance.*

**2.3**- If we want to integrate two modalities where the features are not directly shared how bindSC would perform? For eg. in case of integrating snATAC-seq and Spatial transcriptomics data, how this can be achieved?

***Response:***

*bindSC utilizes the fusion matrix* $\boldsymbol{Z}^{\boldsymbol{(0)}}$ *to link features between the two modalities. In most of application scenarios, there are straightforward prior knowledge to initialize the fusion matrix. For example, in the case of integrating protein and gene expression profiles/chromatin profiles, using direct coding-gene to protein correspondence as anchors to initialize fusion matrix is a feasible way (as shown in original* ***Fig. 3****).*

*The task of integrating snATAC-seq and spatial transcriptomics data, as pointed out by the reviewer, is indeed more challenging. However, similar to integrating scRNA-seq and snATAC-seq, the* ***X*** *matrix can be set as the gene expression matrix from the spatial transcriptomics data and* ***Y*** *as the peak profiles from snATAC-seq data. The fusion matrix* $\boldsymbol{Z}^{\boldsymbol{(0)}}$ *can then be initialized using one of the gene scoring approaches (e.g., summing reads over gene body), similar to scRNA-seq and snATAC-seq integration.*

**2.4**- How bindSC scales to large number of cells. The authors of this paper used 27,181 metal muscle cells from human metal cell atlas to demonstrate applicability of their method. But how bindSC performs if applied to all cells (nearly 800,000)? As the datasets would only be growing in near future it is important that the authors benchmark their method with large datasets.

***Response:***

*This is an important question. bindSC took two measures to handle scalability issue: 1) utilize reduced feature dimension rather than full feature (peak) matrix; 2) utilize the “divide-and-conquer” SVD implementation (original Additional file1:* ***Fig. S1b****). We expect that bindSC runtime is linear to the number of cells and the peak memory usage independent of cell numbers.*

*Because cells collected from different organs (*[*https://descartes.brotmanbaty.org/bbi/human-chromatin-during-development/*](https://descartes.brotmanbaty.org/bbi/human-chromatin-during-development/)*) may have organ specific properties, we perform data integration in each organ independently. To examine scalability, we created ten benchmark datasets of 22,552 to 834,424 cells by resampling cells in the fetal muscle atlas. The block size was set to 1,000 for bindSC in each dataset. We obtained the elapsed run time and maximum memory for all benchmarks using one thread (with a 28-core Intel Skylake CPU@2.6GHz). As expected, bindSC runtime appeared linear to the number of cells, ranging from 4 minutes on 23,000 cells to 184 minutes on 800,000 cells. The peak memory usage was <10GB in all the datasets, regardless of the cell numbers.*

*We have added following results on page 23 (section* ***Results****) and new Additional file1:* ***Fig. S12e****.*

*“To examine bindSC’s scalability in large-scale datasets, we created ten benchmark datasets with cells number ranging from 22,552 to 834,424 by resampling cells in the fetal muscle atlas (****Additional file 1: Fig. S12e****). The block size was set to 1,000 for bindSC in each dataset. We obtained the elapsed run time and maximum memory for all the benchmarks using one thread (with a 28-core Intel Skylake CPU@2.6GHz). As expected, bindSC runtime appeared linear to the number of cells, ranging from 4 minutes for analyzing 23,000 cells to 184 minutes for 800,000 cells. The maximum memory usage was <10GB in all the datasets, regardless of cell numbers.”*

**2.5**- Also can bindSC implementation work on data structures that supports out of core computation? like HDF5 or zarr?

***Response:***

*In the current implementation, bindSC supports sparse matrix format only. As described above, bindSC consistently uses less than 10GB memory for up to 800,000 cells, which may be sufficient for most ongoing studies. We do plan to add support to HDF5/zarr in future improvements, as suggested by the reviewer.*

**2.6**- It was not immediately clear to me how the multi-view in joint embedding would be interpreted. In the multi-view we can project the gene expression (or gene score for snATAC-seq) but we cannot project peak opening for the snRNA-seq datasets. So how an user would use and interpret the multi-view in the joint embedding?

***Response:***

*The reviewer is correct that one cannot directly “project” a peak opening state onto a RNA state via the multi-view of the bindSC co-embedding. The issue is out of the scope of our study. BindSC aims to establish an optimal (in linear correlation sense) association (rather than a projection) between two multiomic states. This is already useful, as it can discover novel biological relations across modalities. Based on the co-embedding, we can create the joint multiomic profiles at pseudo-cell or cluster resolution, enabling multiomic characterization of a cell-type/state. Furthermore, a unique, precise projection between an ATAC and an RNA state may not be biological meaningful, as RNA transcription is a dynamic process that conditions on but is not uniquely determined by a chromatin state. Therefore, it may not be biologically meaningful to define/identify such a projection.*

**Second round of review**

**Reviewer 1**

The authors have satisfactorily responded to my review. My one suggestion for publication is that the analysis of imbalanced integration (what is supplementary figure 4) is moved to a main figure rather than supplement as it is an important analysis that is often uner-discussed in the field.

**Reviewer 2**

The authors have addressed all my comments nicely. I am happy to accept the paper for publication.

**Authors Response**

**Point-by-point responses to the reviewers’ comments:**

Reviewer #1: The authors have satisfactorily responded to my review. My one suggestion for publication is that the analysis of imbalanced integration (what is supplementary figure 4) is moved to a main figure rather than supplement as it is an important analysis that is often under-discussed in the field.

***Response:***

*As suggested, we have now included* ***Fig. S4 d, f,*** ***h*** *as* ***Fig. 2 h, i and j*** *and modified the legends and labels accordingly.*
